# PhyloSim - Monte Carlo simulation of sequence evolution in the R statistical computing environment

**DOI:** 10.1186/1471-2105-12-104

**Published:** 2011-04-19

**Authors:** Botond Sipos, Tim Massingham, Gregory E Jordan, Nick Goldman

**Affiliations:** 1 EMBL-European Bioinformatics Institute, Hinxton, UK; 2 Laboratory of Molecular Biodiversity, Institute of Genetics, Biological Research Center, Szeged, Hungary

## Abstract

**Background:**

The Monte Carlo simulation of sequence evolution is routinely used to assess the performance of phylogenetic inference methods and sequence alignment algorithms. Progress in the field of molecular evolution fuels the need for more realistic and hence more complex simulations, adapted to particular situations, yet current software makes unreasonable assumptions such as homogeneous substitution dynamics or a uniform distribution of indels across the simulated sequences. This calls for an extensible simulation framework written in a high-level functional language, offering new functionality and making it easy to incorporate further complexity.

**Results:**

PhyloSim is an extensible framework for the Monte Carlo simulation of sequence evolution, written in R, using the Gillespie algorithm to integrate the actions of many concurrent processes such as substitutions, insertions and deletions. Uniquely among sequence simulation tools, PhyloSim can simulate arbitrarily complex patterns of rate variation and multiple indel processes, and allows for the incorporation of selective constraints on indel events. User-defined complex patterns of mutation and selection can be easily integrated into simulations, allowing PhyloSim to be adapted to specific needs.

**Conclusions:**

Close integration with R and the wide range of features implemented offer unmatched flexibility, making it possible to simulate sequence evolution under a wide range of realistic settings. We believe that PhyloSim will be useful to future studies involving simulated alignments.

## Background

Monte Carlo simulation of sequence evolution is routinely used in assessing the performance of phylogenetic inference methods (e.g. [1]), multiple sequence alignment algorithms (e.g. [2]) and ancestral reconstruction (e.g. [3]). Monte Carlo simulation of sequence evolution is also crucially important in the testing of competing evolutionary hypotheses [4,5], yet the effect of insertions and deletions (indels) is often ignored since the necessary tools were not available.

Several software packages for simulating basic sequence evolution under popular substitution models have been published in the last decade, for example SDSE[6], Seq-Gen[7] and the evolver program from the PAML package [8]. More recently published software goes beyond the limitations of earlier simulation tools, allowing, for example, the simulation of indel events, sequence regions evolving under different models/parameters, the use of non-homogeneous models allowing for different parameters on different evolutionary lineages (e.g. Dawg[9]; SIMPROT[10]; MySSP[11]; INDELible[12]) and the flexible simulation of genomic features [13].

The R language [14] is the leading open-source environment for statistical computing and graphics, extensively used in bioinformatics data analysis. Its use for the analysis of phylogenetic and evolutionary data is aided by the "Analysis of Phylogenetics and Evolution" (APE) package [15] and a small ecosystem of packages extending its capabilities [16]. The simulation of the evolution of continuous characters is possible using APE and discrete characters can be evolved along a tree according to an arbitrary rate matrix using the phangorn[17] and geiger[18] packages. However, there is no R package currently supporting the simulation of indel events and sequence evolution with site-specific rates, nonsynonymous/synonymous rate ratios or other advanced features available in other phylogenetics software.

Allowing for heterogeneous evolution is a fundamental part of virtually all modern phylogenetic analyses [19] and realistic simulation of indel events is indispensable when benchmarking the performance of multiple alignment methods. Previous software does not handle indels realistically, posing potential problems for the downstream analyses. Most programs assume a uniform distribution of indel events across the simulated biological sequences, despite the fact that those are likely to have regions evolving under different selective constraints [20,21]. Some tools try to address this problem by allowing for partitions evolving under different models/parameters. However, the deletions are often not allowed to cross partition boundaries, which creates an unrealistic "edge effect". The correlation between the selective constraints on indels and substitution [22] is another aspect of sequence evolution which cannot be handled properly just by defining partitions.

Here we present PhyloSim, an object-oriented framework enabling the realistic Monte Carlo simulation of sequence evolution. PhyloSim significantly extends the range of realistic evolutionary patterns that can be simulated, and is freely extensible within the R environment.

## Implementation

The PhyloSim framework - written in pure R - builds on the APE package and aims to complement it. It also uses the R.oo package [23], which provides class-object-oriented facilities with references on top of the default function-object-oriented framework, and depends on the compoisson and ggplot2 packages. The released packages are freely available under the GNU General Public Licence version 3 from CRAN [24] and the package download page [25]. The package sources are also available from the PhyloSim GitHub repository [26].

## Results and Discussion

**Figure 1:**
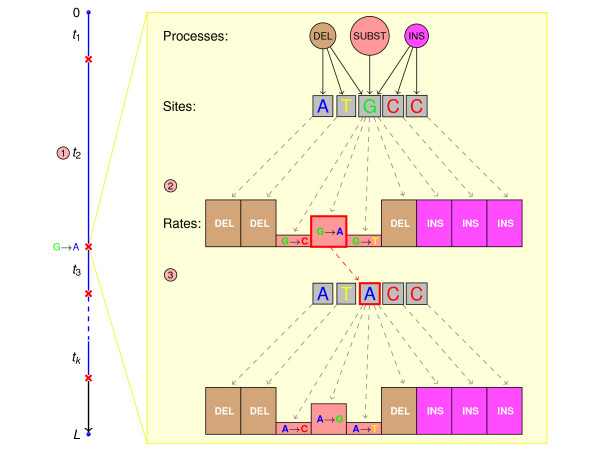
**Illustration of the Gillespie algorithm**. ① The rate at which the next event occurs is equal to the sum of the rates of all possible events; the time, *t_k_*, until event *k *occurs is randomly chosen and the simulation ends if the event would have occurred after the end of the branch (*L*). ② The actual event that occurs is randomly selected, each event having probability proportional to its rate. The figure highlights the event *k *= 2, a G→A substitution at the third site of the evolving sequence. ③ The selected event is applied to the sequence, the set of possible events and their rates are updated and the next inter-event time (*t*_3_) drawn.

PhyloSim uses the Gillespie algorithm [27] as a unified framework for simulating substitutions and other events such as insertions and deletions (Figure 1; see also [12]). Sequence evolution along a branch is simulated in two steps, iterated repeatedly: sampling the time of occurrence of the next event and then modifying the sequence object according to a randomly selected event. The rate of occurrence of the next event is equal to the sum of all possible event rates, while the event to be performed is selected with a probability proportional to its rate. After performing the event, the set of possible events is updated. These steps are repeated until the available time (the length of the branch) is exhausted. As in the case of previous software [9,12], time is defined in terms of expected substitutions per site and the neutral rates of all other processes are specified relative to that.

Selective constraints on different types of events (e.g. deletions) can be incorporated in a natural way in the framework described above by accepting/rejecting the selected event with a probability determined by some of its characteristics (e.g. rejecting deletions based on properties of the affected sites).

The key features offered by PhyloSim are the following:

I. Simulation of the evolution of a set of discrete characters with arbitrary states evolving by a continuous-time Markov process with an arbitrary rate matrix.

II. Explicit implementations of the most popular nucleotide, amino acid and codon substitution models.

III. The possibility to simulate evolution by a combination of substitution processes with arbitrary rate matrices acting on the same site.

IV. Simulation under the popular models of among-sites rate variation, such as the gamma (+Γ) and invariant sites plus gamma (+I+Γ) models.

V. The possibility to simulate with arbitrarily complex patterns of among-sites rate variation by setting the site-specific rates according to any R expression.

VI. Simulation with one or more separate insertion and deletion processes acting on the sequences, each sampling indel lengths from an arbitrary discrete distribution or an R expression (so all probability distributions implemented in R are readily available for this purpose).

VII. All the rate variation features listed above (IV, V) can be readily applied to modify the rates whereby insertion and deletion processes initiate events at given sites.

VIII. Simulation of the effects of spatially variable functional constraints by site- and process-specific insertion and deletion tolerance parameters, which determine the rejection probability of a proposed insertion or deletion ("field deletion and insertion" models; see below); rescaled deletion processes speed up simulation when deletions are strongly selected against ("fast field deletion" model).

Field indel models allow for the fine-grained control of selective constraints on indels and, unlike the partition approach, do not suffer from "edge effect" artifacts.

IX. The possibility of having different processes and site- and process-specific parameters for every site, which allows for an arbitrary number of partitions in the simulated data.

X. Simulation of heterotachy and other cases of time-non-homogeneous evolution by allowing the user to set "node hook" functions altering sites' properties at internal nodes of the phylogeny.

XI. Full control over the properties of the inserted sequences, which makes it possible to easily extend PhyloSim with new kinds of insertion processes, (e.g. duplications; see example 3.3 in the package vignette, included as additional file 1).

The validity of the framework has been tested by simulating the evolution of nucleotide, amino acid and codon sequences of increasing length and estimating the value of model parameters and branch lengths from the resulting alignments using the PAML package [8]. The results are summarized in Appendix A (additional file 2) along with the computing time needed for simulation and estimation. Implementation using R naturally affects the amount of computing time and memory needed for the simulations, but we believe that this is balanced out by the unparalleled versatility offered by the framework.

PhyloSim is provided with extensive documentation. In addition, a package 'vignette' (additional file 1) gives a series of examples illustrating the simulation of successively more complex evolutionary scenarios, from very simple and familiar models through to complicated heterogeneous evolutionary dynamics not available with other software.

### Further details of the field deletion models

A natural way to incorporate deletions into the Gillespie framework is to assign an individual rate to every possible deletion event. Modelling in this manner is extremely general but requires a lot of specification: not only individual sites' tolerance to deletion but also of how they interact with neighbouring sites. Instead we propose a more restricted "field model" of deletion that generalises previous simple approaches to allow the rate at which deletions occur to vary across the sequence but only requires one parameter per site - its deletion tolerance - to be specified. Under this model, deletions are proposed in same manner as other events, specifying a rate of occurrence and a distribution of lengths, and then accepted or rejected based on sites they propose to remove.

Firstly consider only single-site deletions and let each site, *i*, in the sequence have an associated deletion tolerance parameter, *d_i _*∈ [0, 1], representing the probability that it is actually deleted given that a deletion is proposed. Sites where *d_i _*= 1 are deleted at the background rate, sites with *d_i _*< 1 are deleted more slowly, and sites with *d_i _*= 0 are never deleted. For proposed deletions that span multiple sites, ℐ, each site is considered independently and the proposed deletion is accepted if and only if every site accepts it: the total probability of acceptance is therefore Π_*i*∈ℐ _*d_i_*. This scheme allows functionally important "undeletable" sites and regions to be modelled, as well as the phenomenon of deletion hotspots.

It is natural to think of the background rate of deletion as a neutral rate but this is not necessary and can lead to the Gillespie algorithm becoming inefficient: for example, an extremely deletion intolerant sequence will reject almost all deletions proposed and so waste many steps. Instead we can rescale the process and the deletion tolerances ("fast field deletion model") so that deletions are proposed at a rate equal to what would occur if the entire sequence had a deletion tolerance equal to its most tolerant site.

### An example: annotating a simulated alignment using PRANK

**Figure 2:**
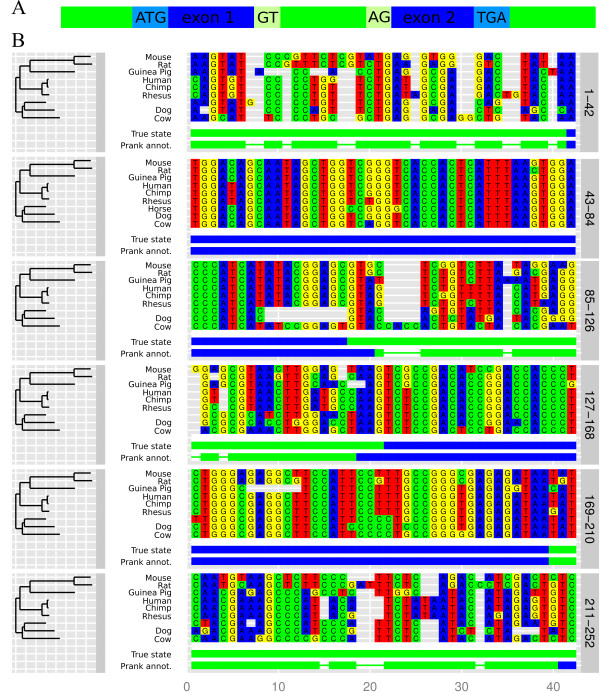
**Annotation of a simulated alignment by using **PRANK**'s genomic structure model**. A. A schematic representation of the structure of the genomic region used in the simulation. Noncoding regions, evolving by a K80 (Kimura two parameters) substitution process [32], are shown in green. Coding regions are shown in blue, and evolve by a GY94 (Goldman-Yang) codon model [33,34]. The other features included in the simulation, the fixed start codon and splicing sites and the stop codon evolving by a special substitution process, are shown in lighter shades. B. A "true" multiple sequence alignment resulting from the simulation of the genomic region along the phylogenetic tree shown to the left. The tracks under the sites represent the true intron-exon structure ("True state") and the annotation of the alignment inferred by PRANK alignment tool transferred to the human sequence ("Prank annot."). The thin portions of the PRANK annotation track indicate positions that have no annotation available as they have gaps in the human sequence in the true simulated alignment.

Simulating sequence evolution is crucial when benchmarking any method which relies on the heterogeneity of the evolutionary signal in multiple alignments (e.g. gene prediction tools). As an example of a potential use of the PhyloSim package, we simulated the evolution of a genomic region containing a small gene with two exons (Figure 2A), which could be a practical way to assess the sensitivity of the genomic structure model [28] implemented in the PRANK phylogeny-aware multiple alignment tool [29].

We simulated the evolution of the genomic region along a phylogenetic tree of nine mammal species (Figure 2B, left). For added realism, we included in the simulation features like fixed start codons and splice sites, and a substitution process acting on the three functionally equivalent stop codons (see the legend of Figure 2A for more details). The R script used for the simulation (example_A1.R) can be found in the examples directory of the package source repository [26].

We used the webPRANK server [30] to align the simulated sequences, and regarded an alignment position to be annotated as coding if the reported posterior probability of any of the three coding states was greater than 0.5. We transferred back the annotation to the "true" simulated multiple alignment through the human sequence and compared it to the true structure of the simulated region (Figure 2B). We found that the exons inferred by PRANK show a good overlap with the true simulated exons.

## Conclusions

With the features listed above, PhyloSim permits simulations encompassing a wide range of complexity (Table 1), from those involving simple indel models similar to TKF91 [31] to realistic simulations of protein sequences containing domains with distinct characteristics as well as of whole genomic regions harbouring coding sequences with intron-exon structures (see examples 3.1, 3.2 and 3.4 in the package vignette). Extensibility is the most prominent feature of the framework, its design making very simple the implementation of new processes embodying novel events (see example 3.3 in the package vignette for an inverted duplication process) and the adaptation of the simulator to whatever is required.

**Table 1:** Comparison of some advanced alignment simulation tools

Key	Feature*	Dawg v1.1.2	MySSP v1.0	Indel-Seq-Gen v1.0.3	SIMPROT v1.01	INDELible v1.0	PhyloSim v0.12
II	GTR	•	•			•	•
II	UNREST					•	•
II	Empirical amino acid models			3	3	15	11
II	User defined amino acid models					•	•
II	Codon models					•	•
III	Combinations of substitution processes						•
IV	Discrete gamma					•	•
IV	Continuous gamma	•	•		•	•	•
IV	Proportion of invariant sites	•		•		•	•
V	Complex rate variation						•
VI	Multiple indel processes						•
VII	Rate variation with indel processes						•
VIII	Selective constraints on indels						•
IX	Partitions		•	•	•	•	•
X	Non-homogeneous evolution		•			•	•
XI	Full control over inserts						•

Availability of complex evolutionary processes in different simulation software. Additional details for less advanced software and simpler models are given by [[12], Table 1].

*See text for details.

## Availability and Requirements

• **Project name: **PhyloSim

• **Project home page: **http://www.ebi.ac.uk/goldman-srv/phylosim

• **Project source repository: **http://github.com/sbotond/phylosim

• **Operating system(s): **OS Independent (Written in an interpreted language)

• **Programming language: **R

• **Required **R** packages: **R.oo (≥ 1.4.6), ape (≥ 2.3), compoisson (≥ 0.3), ggplot2 (≥ 0.8.8)

• **License: **GNU General Public License Version 3

• **Any restrictions to use by non-academics: **none

## Authors' contributions

BS, TM and NG designed the framework. BS implemented the framework and obtained the test results. GJ contributed the alignment and tree plotting methods. BS drafted the manuscript, which was reviewed and approved by all authors.

## Supplementary Material

Additional file 1 **The **PhyloSim** package vignette**. Click here for file

Additional file 2 **Appendix A**. Click here for file
