# Supplementary material for: PhyloSim - Monte Carlo simulation of sequence evolution in the R statistical computing environment
Source: BMC Bioinformatics. 2011 Apr 19;12:104. doi: 10.1186/1471-2105-12-104 (PMC3102636; doi:10.1186/1471-2105-12-104)
Supplement: Additional file 1 — The PhyloSim package vignette. [file 1471-2105-12-104-S1.PDF]

The `PhyloSim` package

Botond Sipos and Tim Massingham

February 2, 2011

Contents

1 Getting help **2**

2 Basic examples **2**

2.1 Simulating substitutions under the JC69 model 2

2.1.1 Compact example 2

2.1.2 "Unrolled" example 3

2.2 Simulating substitutions under the HKY model 5

2.3 Simulating among-sites rate variation 7

2.3.1 Simulating under the discrete gamma (GTR+dΓ) model 7

2.3.2 Simulating under the invariants and discrete gamma (GTR+I+dΓ) model 8

2.4 Simulating indels 9

2.4.1 Insertion and deletions having the same length distribution 9

2.4.2 Insertion and deletions having different length distributions 10

2.4.3 Simulating indels under selective constraints 11

2.5 Simulating partitions 13

2.6 Simulating heterotachy 14

2.7 Simulating many replications 16

3 Advanced examples **17**

3.1 Simulating "domains" and heterogeneous evolution 17

3.2 Evolving codon sequences 24

3.3 Implementing a new process 29

3.4 Evolving a genomic region containing a "gene" 31

4 Details of the fast field deletion model **36**

1 Getting help

- `PhyloSim` is extensively documented; the documentation of the `PhyloSim` class (as invoked by `help("PhyloSim")`) is probably a good entry point for beginners and also contains some more basic examples.
- Serious users might consider reading all class documentations which provide more focused examples.
- Additional example scripts can be found at http://github.com/sbotond/phylosim/tree/master/examples
- The `ll()` method list the methods and virtual field implemented in the immediate class of an object which is useful as "proto-documentation".

2 Basic examples

The following examples illustrate how to set up simple simulations.

First load the package:

```
> library(phylosim)
```

Create the tree files used in the basic examples:

```
> cat("((t1:0.3,t2:0.3):0.2,t3:0.1);", file = "3taxa.nwk")
> cat("((t1:0.3,t2:0.3):0.2,(t3:0.1,t4:0.1):0.4);", file = "4taxa.nwk")
```

2.1 Simulating substitutions under the JC69 model

We will simulate the evolution of nucleotide sequences of length 50 along a tree with 3 tips under the JC69 model. The following two examples are essentially equivalent.

2.1.1 Compact example

```
> Simulate(PhyloSim(root.seq = sampleStates(NucleotideSequence(len = 50,
+     proc = list(list(JC69())))), phylo = read.tree("3taxa.nwk")))$alignment
```

```
Simulating edge 1 of 4
Simulating edge 2 of 4
Simulating edge 3 of 4
Simulating edge 4 of 4
            1   2   3   4   5   6   7   8   9   10  11  12  13  14  15  16  17  18  19
Root node 4 "C" "G" "A" "A" "A" "A" "T" "A" "C" "G" "T" "G" "T" "T" "A" "C" "A" "T" "T"
t3          "C" "G" "A" "A" "T" "A" "T" "A" "C" "G" "T" "T" "T" "T" "A" "C" "A" "T" "T"
Node 5      "C" "G" "A" "A" "A" "A" "T" "A" "C" "A" "T" "C" "T" "G" "A" "C" "A" "T" "T"
t2          "C" "G" "A" "A" "A" "T" "T" "A" "G" "T" "T" "C" "T" "G" "T" "C" "A" "C" "T"
t1          "C" "T" "A" "A" "A" "A" "T" "A" "C" "A" "T" "A" "T" "G" "A" "T" "A" "T" "T"
            20  21  22  23  24  25  26  27  28  29  30  31  32  33  34  35  36  37  38
Root node 4 "C" "C" "G" "C" "G" "G" "C" "T" "T" "C" "A" "A" "A" "G" "G" "T" "T" "A" "C"
t3          "C" "C" "C" "C" "G" "G" "C" "T" "T" "G" "A" "A" "A" "G" "G" "T" "T" "A" "C"
Node 5      "C" "C" "G" "C" "G" "G" "T" "A" "T" "A" "A" "A" "A" "G" "G" "T" "T" "A" "C"
t2          "C" "C" "G" "G" "G" "G" "T" "T" "G" "A" "A" "G" "A" "C" "G" "T" "T" "A" "C"
t1          "C" "C" "G" "C" "G" "G" "C" "A" "T" "C" "A" "A" "A" "G" "G" "T" "T" "A" "C"
```

```
             39  40  41  42  43  44  45  46  47  48  49  50
Root node 4 "T" "C" "G" "T" "A" "A" "C" "T" "T" "G" "G" "A"
t3          "T" "C" "G" "T" "A" "A" "C" "T" "T" "G" "G" "A"
Node 5      "T" "C" "A" "T" "T" "T" "C" "T" "T" "G" "G" "T"
t2          "T" "G" "A" "T" "T" "T" "C" "C" "T" "C" "A" "T"
t1          "T" "C" "C" "T" "T" "T" "C" "T" "T" "T" "G" "T"
```

2.1.2 "Unrolled" example

Construct the root sequence object:

```
> root.seq <- NucleotideSequence(length = 50)
```

Print out the root sequence:

```
> print(root.seq)
```

```
[1] "??????????????????????????????????????????????????"
```

Note, that all states are still undefined.

Construct a JC69 substitution process object:

```
> p <- JC69()
```

Attach the substitution process to root sequence:

```
> attachProcess(root.seq, p)
```

Sample the site states from the equilibrium distribution of the JC69 substitution process:

```
> sampleStates(root.seq)
```

Print out the root sequence, now with the sampled states:

```
> print(root.seq)
```

```
[1] "CTAGAAATGTTCGCTCTCTAACATACGGTACTTAAACACCTCCCGCACAC"
```

Read in tree from file using APE:

```
> tree <- read.tree("3taxa.nwk")
```

Plot the tree:

```
> plot(tree)
```

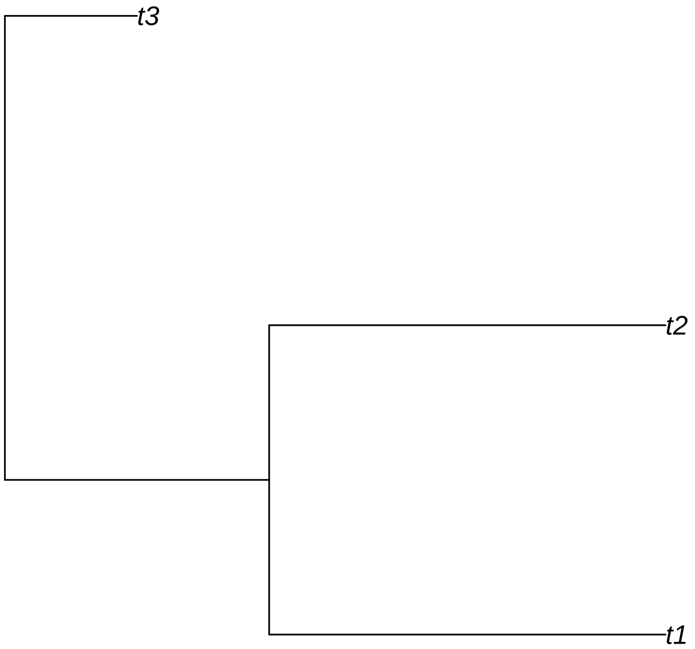

Construct a `PhyloSim` object:

```
> sim <- PhyloSim()
```

Set the phylo object:

```
> sim$phylo <- tree
```

Set the root sequence:

```
> sim$rootSeq <- root.seq
```

Run the simulation:

```
> Simulate(sim)
```

```
Simulating edge 1 of 4
Simulating edge 2 of 4
Simulating edge 3 of 4
Simulating edge 4 of 4
```

Display the resulting alignment matrix:

```
> sim$alignment
```

```
            1   2   3   4   5   6   7   8   9   10  11  12  13  14  15  16  17  18  19 
Root node 4 "C" "T" "A" "G" "A" "A" "A" "T" "G" "T" "T" "C" "G" "C" "T" "C" "T" "C" "T"
t3          "C" "T" "A" "G" "A" "A" "A" "T" "G" "G" "T" "C" "G" "C" "T" "C" "T" "C" "C"
Node 5      "C" "T" "A" "A" "A" "A" "A" "T" "G" "T" "T" "C" "G" "C" "T" "G" "T" "C" "T"
t2          "C" "T" "G" "G" "A" "A" "A" "T" "C" "T" "T" "C" "G" "C" "A" "G" "T" "A" "T"
t1          "A" "T" "A" "A" "A" "A" "A" "T" "C" "A" "T" "C" "G" "C" "T" "G" "T" "C" "G"
            20  21  22  23  24  25  26  27  28  29  30  31  32  33  34  35  36  37  38 
Root node 4 "A" "A" "C" "A" "T" "A" "C" "G" "G" "T" "A" "C" "T" "T" "A" "A" "A" "C" "A"
t3          "A" "A" "C" "A" "T" "A" "C" "G" "G" "T" "A" "C" "T" "T" "A" "A" "A" "C" "A"
Node 5      "A" "A" "A" "A" "T" "A" "C" "G" "T" "T" "A" "C" "T" "T" "G" "A" "A" "T" "A"
t2          "A" "G" "A" "A" "T" "C" "C" "G" "T" "T" "A" "C" "T" "T" "G" "A" "A" "T" "A"
t1          "C" "A" "A" "A" "T" "A" "C" "G" "G" "T" "A" "T" "C" "T" "A" "A" "A" "T" "G"
            39  40  41  42  43  44  45  46  47  48  49  50 
Root node 4 "C" "C" "T" "C" "C" "C" "G" "C" "A" "C" "A" "C"
t3          "C" "C" "C" "C" "C" "C" "G" "C" "A" "C" "G" "G"
Node 5      "C" "A" "T" "C" "C" "G" "G" "T" "A" "A" "G" "C"
t2          "C" "A" "T" "C" "A" "G" "G" "T" "A" "T" "T" "T"
t1          "A" "A" "C" "C" "C" "G" "G" "T" "A" "C" "T" "C"
```

2.2 Simulating substitutions under the HKY model

Construct an HKY substitution process object:

```
> p <- HKY(rate.params = list(Alpha = 10, Beta = 2), base.freqs = c(4,
+     3, 2, 1)/10)
```

Get a plot of the instantaneous substitution matrix and equilibrium distribution of the `p` process (bubble plot):

```
> plot(p, scale = 0.5)
```

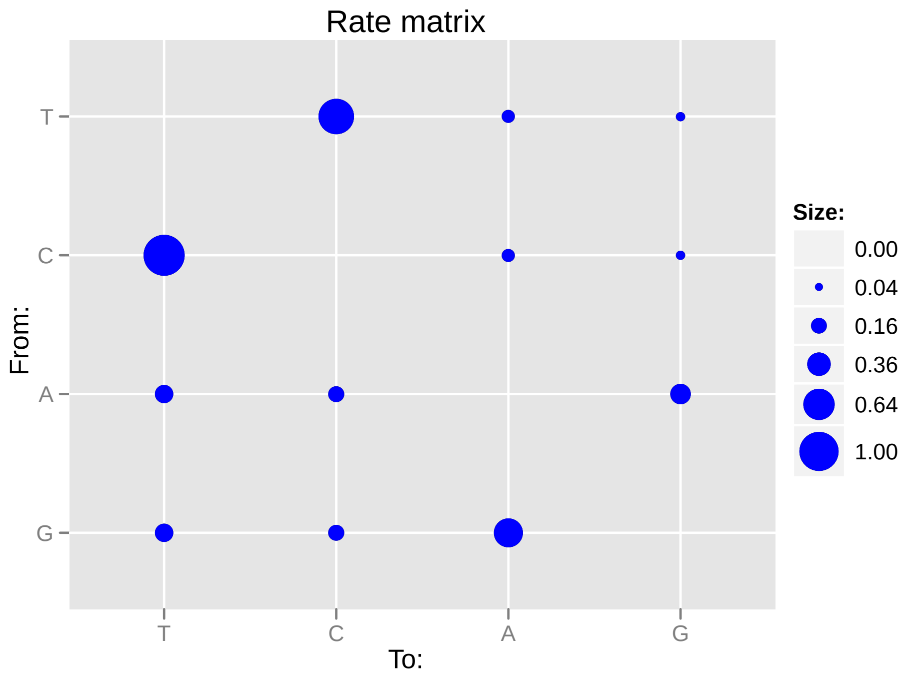

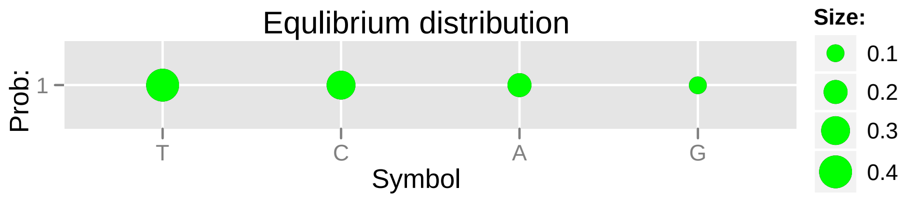

Construct the root sequence, attach the substitution process to root sequence via the constructor and sample states:

```
> root.len50.seq <- NucleotideSequence(length = 50, processes = list(list(p)))
> sampleStates(root.len50.seq)
```

Print out root sequence:

```
> print(root.len50.seq)
```

```
[1] "AAACCTATTCGTATTCTTGTTTTTTTCTCCCAGATTTTTTCCTTATTTAC"
```

Construct a `PhyloSim` object, set the phylo object and the root sequence:

```
> sim <- PhyloSim(root.seq = root.len50.seq, phylo = read.tree("3taxa.nwk"))
```

Run simulation:

```
> Simulate(sim)
```

```
Simulating edge 1 of 4
Simulating edge 2 of 4
Simulating edge 3 of 4
Simulating edge 4 of 4
```

Save the resulting alignment (fasta format):

```
> saveAlignment(sim, file = "HKY_sim.fas")
```

Plot the alignment alongside the tree (including sequences at ancestral nodes):

```
> plot(sim, num.pages = 1)
```

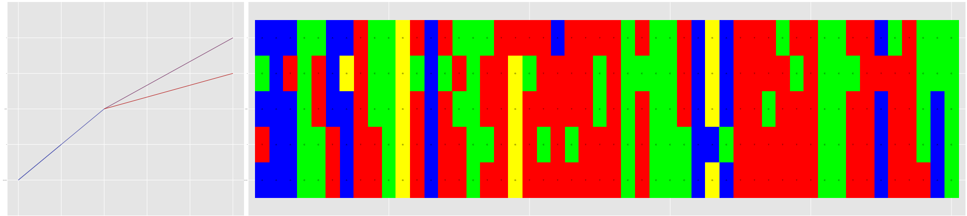

2.3 Simulating among-sites rate variation

2.3.1 Simulating under the discrete gamma (GTR+dΓ) model

Construct a GTR subsection process object:

```
> p <- GTR(rate.params = list(a = 1, b = 2, c = 3, d = 1, e = 2, f = 3),
+     base.freqs = c(2, 2, 1, 1)/6)
```

Summary of object p:

```
> summary(p)
```

```
Name: Anonymous
Id: GTR:Anonymous:99008104
Alphabet:
  Type: Nucleotide
  Symbols: T C A G
Rate parameters: a = 1, b = 2, c = 3, d = 1, e = 2, f = 3
Unscaled rate matrix:
                   T          C          A          G
        T -1.1666667  0.3333333  0.3333333  0.5000000
        C  0.3333333 -0.8333333  0.1666667  0.3333333
        A  0.6666667  0.3333333 -1.5000000  0.5000000
        G  1.0000000  0.6666667  0.5000000 -2.1666667

Equilibrium distribution:
                      T         C         A         G
        Prob: 0.3333333 0.3333333 0.1666667 0.1666667

Site specific parameters (1):
  Id: rate.multiplier
  Name: Rate multiplier
  Type: numeric
  Default value: 1
```

Construct the root sequence, attach substitution process:

```
> root.seq <- NucleotideSequence(length = 50, processes = list(list(p)))
```

Sample rate multipliers from a discrete gamma distribution with 4 categories and shape parameter 0.5:

```
> plusGamma(root.seq, p, 0.5)
```

Get the sampled rate multipliers:

```
> getRateMultipliers(root.seq, p)
```

```
 [1] 0.25191592 0.03338775 0.25191592 2.89442785 0.25191592 0.82026848 0.82026848
 [8] 0.03338775 0.82026848 0.25191592 2.89442785 0.82026848 0.03338775 2.89442785
[15] 0.03338775 0.82026848 0.82026848 0.25191592 0.25191592 0.82026848 0.25191592
[22] 2.89442785 0.82026848 2.89442785 0.03338775 0.03338775 0.82026848 0.25191592
[29] 0.25191592 0.25191592 0.82026848 2.89442785 0.03338775 0.82026848 0.82026848
[36] 0.82026848 0.82026848 0.25191592 2.89442785 0.25191592 0.25191592 0.82026848
[43] 0.82026848 0.82026848 0.25191592 0.03338775 2.89442785 2.89442785 0.25191592
[50] 2.89442785
```

Construct the `PhyloSim` object, sample states and set the phylo object:

```
> sim <- PhyloSim(root.seq = sampleStates(root.seq), phylo = read.tree("3taxa.nwk"))
```

Run the simulation:

```
> Simulate(sim)
```

```
Simulating edge 1 of 4
Simulating edge 2 of 4
Simulating edge 3 of 4
Simulating edge 4 of 4
```

Plot the alignment alongside the tree, skip sequences at ancestral nodes:

```
> plot(sim, num.pages = 1, plot.ancestors = FALSE)
```

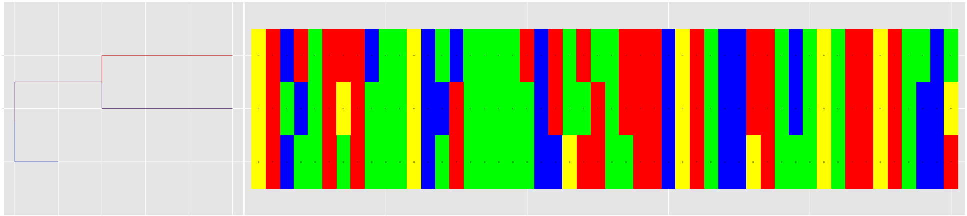

Save the resulting alignment, omitting sequences at internal nodes:

```
> saveAlignment(sim, file = "Gamma_sim.fas", skip.internal = TRUE)
```

2.3.2 Simulating under the invariants and discrete gamma (GTR+I+dΓ) model

We will reuse the root sequence object and GTR object from the previous example. The process is already attached, but we need to clear the states to have a new root sequence.

Clear the states of the root sequence object and sample a set of new states:

```
> clearStates(root.seq)
> sampleStates(root.seq)
```

Sample rate multipliers from a +I+dΓ model:

```
> plusInvGamma(root.seq, p, pinv = 0.8, shape = 0.5)
```

Deal with the rest of the simulation (note that for variety we use a random coalescent tree in this example):

```
> sim <- Simulate(PhyloSim(root.seq = root.seq, phylo = rcoal(3)))

Simulating edge 1 of 4
Simulating edge 2 of 4
Simulating edge 3 of 4
Simulating edge 4 of 4

> plot(sim, num.pages = 1, plot.ancestors = FALSE)
```

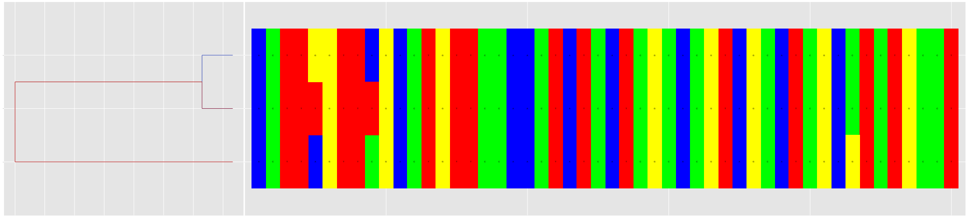

Save the resulting alignment, skip sequences from internal nodes:

```
> saveAlignment(sim, file = "InvGamma_sim.fas", skip.internal = TRUE)
```

2.4 Simulating indels

2.4.1 Insertion and deletions having the same length distribution

Set up the substitution process and the root sequence:

```
> p <- JC69()
> root.seq <- NucleotideSequence(len = 50, processes = list(list(p)))
> sampleStates(root.seq)
```

Construct a deletion process proposing deletions with rate 0.25 according to a discrete length distribution:

```
> d <- DiscreteDeletor(rate = 0.25, sizes = c(1, 2), probs = c(1/2, 1/2))
```

Construct an insertion process proposing insertions with rate 0.25 according to a discrete length distribution:

```
> i <- DiscreteInsertor(rate = 0.25, sizes = c(1, 2), probs = c(1/2, 1/2))
```

Set the template sequence for the insertion process:

```
> i$templateSeq <- NucleotideSequence(length = 2, processes = list(list(p)))
```

The states of the template sequence are undefined. The actual states will be sampled from the equilibrium distribution of the attached substitution process before performing the insertion.

Attaching the indel processes:

```
> attachProcess(root.seq, d)
> attachProcess(root.seq, i)
```

Construct the `PhyloSim` object, set the phylo object (random coalescent tree for three taxa) and run the simulation:

```
> sim <- Simulate(PhyloSim(root.seq = root.seq, phylo = rcoal(3)))
```

```
Simulating edge 1 of 4
Simulating edge 2 of 4
Simulating edge 3 of 4
Simulating edge 4 of 4
```

Plot the alignment alongside the tree, skip sequences at ancestral nodes:

```
> plot(sim, num.pages = 1, plot.ancestors = FALSE)
```

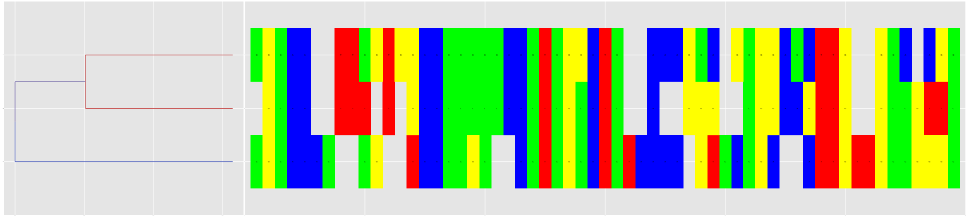

Save the resulting alignment, skip sequences from internal nodes:

```
> saveAlignment(sim, file = "indel1_sim.fas", skip.internal = TRUE)
```

2.4.2 Insertion and deletions having different length distributions

The following code reuses the objects constructed in the previous example to simulate deletions and insertions with different length distributions.

Construct a new insertion process proposing insertions of `C`s with rate 0.5 according to a discrete length distribution:

```
> i2 <- DiscreteInsertor(rate = 0.25, sizes = c(1, 2, 3, 4), probs = c(1,
+     2, 3, 4)/10, template.seq = NucleotideSequence(string = "C"))
```

Clear the states of the root sequence object:

```
> clearStates(root.seq)
```

Define a new set of processes (`p` - substitution, `d` - deletion, `i2` - the new insertion process) for the root sequence using the `processes` virtual field:

```
> root.seq$processes <- list(list(i2, d, p))
```

Construct the PhyloSim object, sample states, set the phylo object and run the simulation:

```
> sim <- Simulate(PhyloSim(root.seq = sampleStates(root.seq), phylo = read.tree("3taxa.nwk")))

Simulating edge 1 of 4
Simulating edge 2 of 4
Simulating edge 3 of 4
Simulating edge 4 of 4
```

Plot the alignment alongside the tree, skip sequences at ancestral nodes:

```
> plot(sim, num.pages = 1, plot.ancestors = FALSE)
```

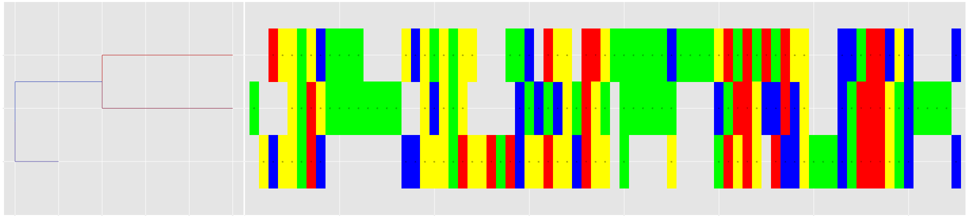

Save the resulting alignment, skip sequences from internal nodes:

```
> saveAlignment(sim, file = "indel2_sim.fas", skip.internal = TRUE)
```

2.4.3 Simulating indels under selective constraints

Set up the substitution process and the root sequence:

```
> p <- JC69()
> root.seq <- NucleotideSequence(len = 100, processes = list(list(p)))
> sampleStates(root.seq)
```

Construct a deletion process proposing deletions with rate 1 according to a discrete length distribution:

```
> d <- DiscreteDeletor(rate = 1, sizes = c(1, 2, 3), probs = c(3/6, 2/6,
+     1/6))
```

Construct an insertion process proposing insertions with rate 1 according to a discrete length distribution:

```
> i <- DiscreteInsertor(rate = 1, sizes = c(1, 2, 3), probs = c(3/6, 2/6,
+     1/6))
```

Set the template sequence for the insertion process:

```
> i$templateSeq <- NucleotideSequence(length = 2, processes = list(list(p)))
```

Attaching the indel processes:

```
> attachProcess(root.seq, d)
> attachProcess(root.seq, i)
```

Set deletion tolerance values:

```
> setDeletionTolerance(root.seq, d, 0.08 + c(1/2:51, 1/51:2))
```

Plot deletion tolerance values:

```
> plotParametersAtSites(root.seq, d, "deletion.tolerance")
```

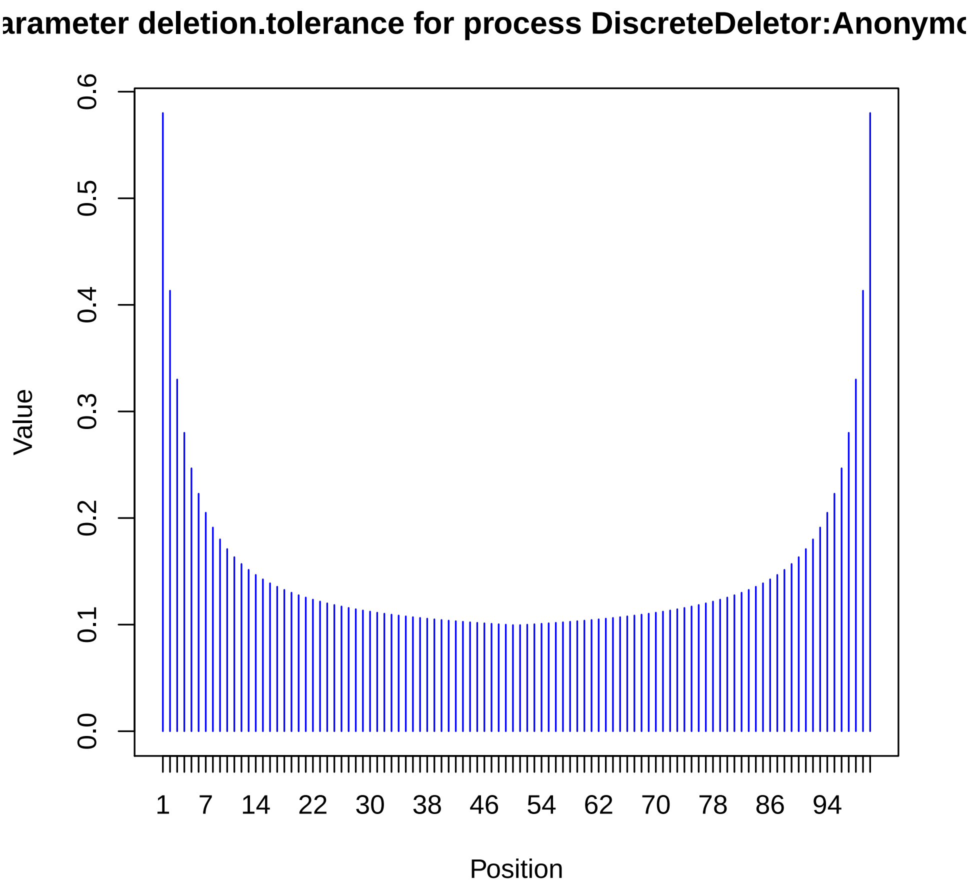

Set insertion tolerance values:

```
> setInsertionTolerance(root.seq, i, 0.08 + c(1/2:51, 1/51:2))
```

Construct the PhyloSim object, read phylo object from file:

```
> sim <- Simulate(PhyloSim(root.seq = root.seq, phylo = read.tree("4taxa.nwk")))
```

```
Simulating edge 1 of 6
Simulating edge 2 of 6
Simulating edge 3 of 6
Simulating edge 4 of 6
Simulating edge 5 of 6
Simulating edge 6 of 6
```

Plot the alignment alongside the tree, skip sequences at ancestral nodes:

```
> plot(sim, plot.ancestors = FALSE)
```

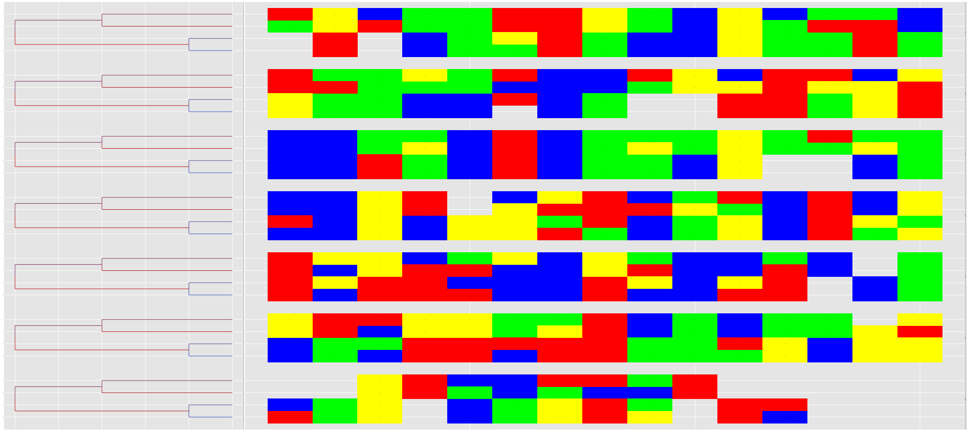

Save the resulting alignment, skip sequences from internal nodes:

```
> saveAlignment(sim, file = "indel3_sim.fas", skip.internal = TRUE)
```

2.5 Simulating partitions

The following example demonstrates how to use the processes and site- and process-specific parameters to simulate "partitions" with different properties.

We will simulate four partitions:

- Partition 1: sites in range `1:25` evolving by JC+dΓ with a shape parameter $\alpha = 1$
- Partition 2: sites in range `26:50` evolving by JC+dΓ with a shape parameter $\alpha = 0.5$
- Partition 3: sites in range `51:75` evolving by HKY+dΓ with a shape parameter $\alpha = 1$
- Partition 4: sites in range `76:100` evolving by HKY+dΓ with a shape parameter $\alpha = 0.5$

First construct two substitution process objects:

```
> jc69 <- JC69()
> hky <- HKY(rate.params = list(Alpha = 5, Beta = 2), base.freqs = c(4,
+     3, 2, 1)/10)
```

Construct a root sequence object of length 100:

```
> root.seq <- NucleotideSequence(length = 100)
```

Attach process `jc69` to range `1:50`:

```
> attachProcess(root.seq, jc69, 1:50)
```

Attach process `hky` to range `51:100`:

```
> attachProcess(root.seq, hky, 51:100)
```

Sample rate multipliers in the four partitions:

```
> plusGamma(root.seq, jc69, 1, 1:25)
> plusGamma(root.seq, jc69, 0.5, 26:50)
> plusGamma(root.seq, hky, 1, 51:75)
> plusGamma(root.seq, hky, 0.5, 76:100)
```

Construct the PhyloSim object, sample states, set root sequence, set the phylo object (random coalescent tree for three taxa) and run the simulation:

```
> sim <- Simulate(PhyloSim(root.seq = sampleStates(root.seq), phylo = rcoal(3)))

Simulating edge 1 of 4
Simulating edge 2 of 4
Simulating edge 3 of 4
Simulating edge 4 of 4
```

Plot the alignment alongside the tree, skip sequences at ancestral nodes:

```
> plot(sim, num.pages = 1, plot.ancestors = FALSE)
```

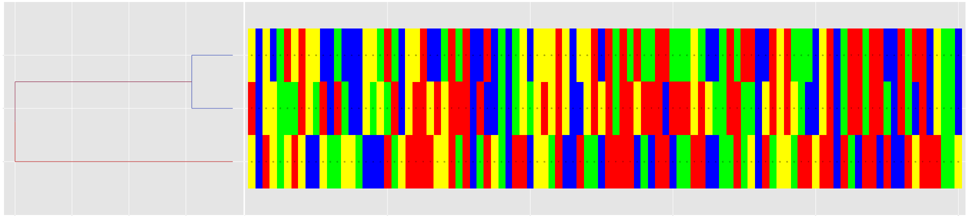

2.6 Simulating heterotachy

Set up the substitution process:

```
> p <- F84(base.freqs = c(1, 2, 3, 4))
> p$kappa <- 1
```

Set up the root sequence:

```
> root.seq <- NucleotideSequence(length = 50, processes = list(list(p)))
```

Sample the rate multipliers from a +dΓ model with shape parameter 1.0:

```
> plusGamma(root.seq, p, 1)
```

Read a tree from file:

```
> tree <- read.tree("4taxa.nwk")
```

Construct the PhyloSim object, sample states, set root sequence, and set the phylo object:

```
> sim <- PhyloSim(root.seq = sampleStates(root.seq), phylo = tree)
```

A "node hook" is a function which accepts a `Sequence` object through the named argument "seq" and returns a `Sequence` object. After simulating the branch leading to the node, the resulting `Sequence` object is passed to the node hook and the returned object is used to simulate the downstream branches.

Create a node hook function which will create heterotachy by resampling the site rates from the +dΓ model with shape parameter 1.0:

```
> node.hook <- function(seq) {
+     if (!isAttached(seq$sites[[1]], p)) {
+         return(seq)
+     }
+     cat("Resampling rate multipliers!\n")
+     plusGamma(seq, p, 1)
+     return(seq)
+ }
```

Plot the `PhyloSim` object:

```
> plot(sim)
```

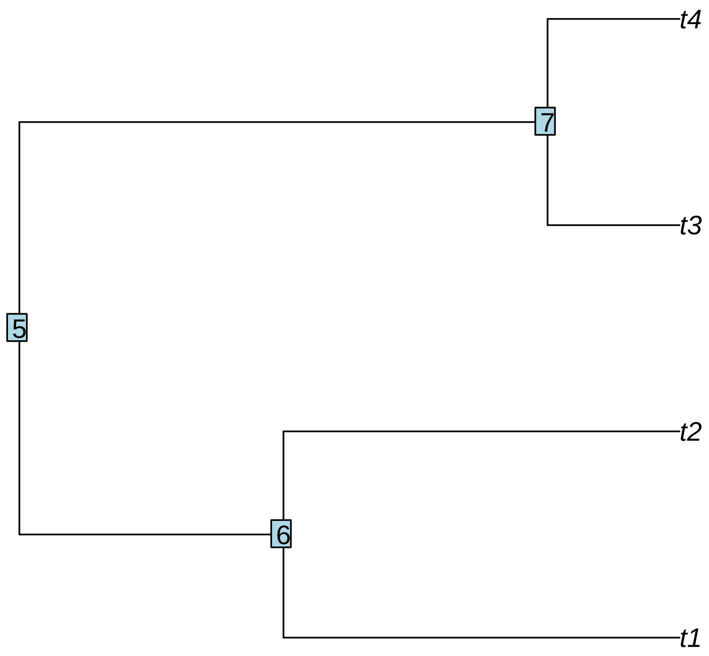

Notice that node 6 is the ancestor of taxa `t1` and `t2`.

Attach the hook to node 6:

```
> attachHookToNode(sim, node = 6, fun = node.hook)
```

Run the simulation:

```
> Simulate(sim)
```

```
Simulating edge 1 of 6
Resampling rate multipliers!
Simulating edge 2 of 6
Simulating edge 3 of 6
Simulating edge 4 of 6
Simulating edge 5 of 6
Simulating edge 6 of 6
```

Plot the alignment alongside the tree, skip sequences at ancestral nodes:

```
> plot(sim, num.pages = 1, plot.ancestors = FALSE)
```

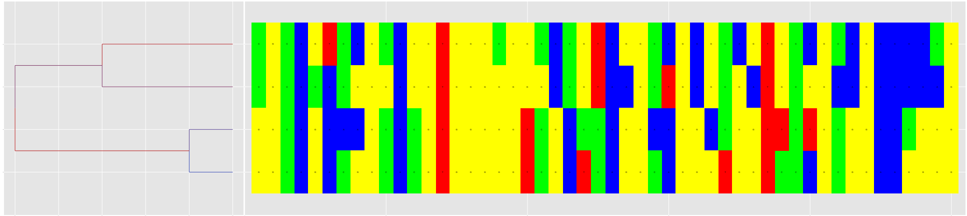

2.7 Simulating many replications

Constructing `Sequence` objects with a large number of sites is expensive, so it is a good idea to do that outside the cycle when simulating many replications with the same root sequence length.

Reusing the root sequence object is easy, but do not forget to do the modifications needed to get independent simulations (e.g. clearing the states of the root sequence, resampling the rate multipliers).

The following code illustrates how to simulate many replications under the JC69+dΓ model.

Construct the root sequence object and attach the substitution process:

```
> p <- JC69()
> root.seq <- NucleotideSequence(length = 10)
> attachProcess(root.seq, p)
```

Read the required phylogeny from file (this will remain fixed in the simulated replications):

```
> tree <- read.tree("3taxa.nwk")
```

Simulate three replications. Note that the states are cleared and resampled; the rate multipliers are resampled as well. The resulting aligments are stored in files aln_1.fas, aln_2.fas, aln_3.fas.

```
> for (i in 1:3) {
+     cat(paste("\n\nSimulating replication ", i, "\n\n", sep = ""))
+     clearStates(root.seq)
+     plusGamma(root.seq, p, 0.25)
```

```
+     sampleStates(root.seq)
+     sim <- Simulate(PhyloSim(root.seq = root.seq, phylo = tree))
+     saveAlignment(sim, file = paste("aln_", i, ".fas", sep = ""))
+ }
```

```
Simulating replication 1

Simulating edge 1 of 4
Simulating edge 2 of 4
Simulating edge 3 of 4
Simulating edge 4 of 4


Simulating replication 2

Simulating edge 1 of 4
Simulating edge 2 of 4
Simulating edge 3 of 4
Simulating edge 4 of 4


Simulating replication 3

Simulating edge 1 of 4
Simulating edge 2 of 4
Simulating edge 3 of 4
Simulating edge 4 of 4
```

3 Advanced examples

Creating the tree file used in the advanced examples:

```
> cat("(((t2:0.1231,t4:0.1231):0.2131,(t3:0.0284,t5:0.0284):0.3078):0.1698,t1:0.5060);",
+     file = "smalldemotree.nwk")
```

3.1 Simulating "domains" and heterogeneous evolution

The following code illustrates how to set up a more complicated simulation of amino acid sequences involving "domains" and heterogeneous evolution.

Use the `ll()` method to list the methods and virtual fields implemented in the `Site` class:

```
> ll(Site())
```

```
Class: Site
Inherits from: PSRoot Object
Fields (0):
Virtual fields (7):
  alphabet
  ancestral
  events
  processes
```

```
  sequence
  state
  totalRate
Methods implemented in Site (8):
  as.character
  attachProcess
  checkConsistency
  detachProcess
  flagTotalRate
  is
  isAttached
  summary
```

Enable the "fast & careless mode":

```
> PSIM_FAST <- TRUE
```

Construct substitution process objects:

```
> wag <- WAG()
> jtt <- JTT()
> lg <- LG()
> pam <- PAM()
```

Summary of the object wag:

```
> summary(wag)
```

```
Name: Anonymous
Id: WAG:Anonymous:89976504
Alphabet:
  Type: Amino acid
  Symbols: A R N D C Q E G H I L K M F P S T W Y V
PAML data file:: PAMLdat/wag.dat
Unscaled rate matrix: not shown
Equilibrium distribution:
                       A          R          N          D         C         Q         E
        Prob: 0.08662791 0.04397200 0.03908940 0.05704511 0.0193078 0.0367281 0.0580589
                       G          H          I        L         K         M         F          P
        Prob: 0.08325181 0.02443130 0.04846600 0.086209 0.0620286 0.0195027 0.0384319 0.04576310
                      S          T          W         Y          V
        Prob: 0.0695179 0.06101271 0.01438590 0.0352742 0.07089561

Site specific parameters (1):
  Id: rate.multiplier
  Name: Rate multiplier
  Type: numeric
  Default value: 1
```

Get a plot of the instantaneous substitution matrix and equilibrium distribution of the wag process (bubble plot):

```
> plot(wag, scale = 0.8)
```

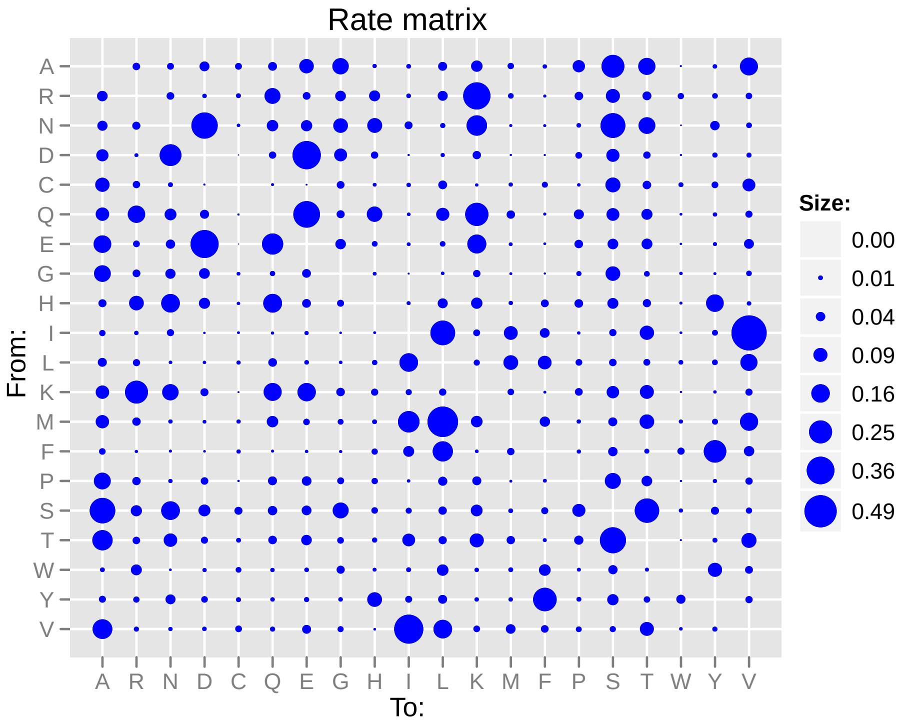

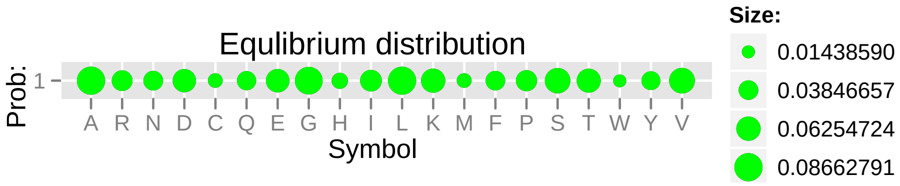

Construct a continuous deletor process:

```
> cont.del <- ContinuousDeletor(rate = 0.6, max.length = 10, dist = expression(rnorm(1,
+     mean = 5, sd = 3)))
```

Construct the template sequence for the `cont.ins.lg` insertion process:

```
> templ.seq.wag <- AminoAcidSequence(length = 10)
```

Clone the template sequence for the `cont.ins.wag` process:

```
> templ.seq.lg <- clone(templ.seq.wag)
```

Construct continuous insertor process object `cont.ins.wag`:

```
> cont.ins.wag <- ContinuousInsertor(rate = 0.6, max.length = 10, dist = expression(rnorm(1,
+     mean = 5, sd = 3)))
```

Construct continuous insertor process object `cont.ins.lg`:

```
> cont.ins.lg <- ContinuousInsertor(rate = 0.6, max.length = 10, dist = expression(rnorm(1,
+     mean = 5, sd = 3)))
```

Set up the template sequences for the insertion processes:

```
> processes.site.wag <- list(wag, cont.ins.wag, cont.del)
> processes.site.lg <- list(lg, cont.ins.lg, cont.del)
> templ.seq.wag$processes <- list(processes.site.wag)
> templ.seq.lg$processes <- list(processes.site.lg)
```

Now the `cont.ins.lg` process samples the states from the equilibrium distribution of the `LG` model and `cont.ins.wag` samples the states from the `WAG` model.

Disabling write protection for the insertion processes:

```
> cont.ins.wag$writeProtected <- FALSE
> cont.ins.lg$writeProtected <- FALSE
```

Set the template sequence for the insertion processes:

```
> cont.ins.wag$templateSeq <- templ.seq.wag
> cont.ins.lg$templateSeq <- templ.seq.lg
```

Setting up the insert hook for the insertion processes:

Insert hook functions are called just before inserting the sequence generated by the insertion process. This function allows for arbitrary modifications to be made to the inserted sequence object. In this case the insert hook functions sample the site-process specific rate multipliers of the substitution processes from an invariants plus discrete gamma (+I+dΓ) model:

```
> cont.ins.wag$insertHook <- function(seq, target.seq, event.pos, insert.pos) {
+     plusInvGamma(seq, process = wag, pinv = 0.1, shape = 1)
+     return(seq)
+ }
> cont.ins.lg$insertHook <- function(seq, target.seq, event.pos, insert.pos) {
+     plusInvGamma(seq, process = lg, pinv = 0.1, shape = 1)
+     return(seq)
+ }
```

Now the processes are in place, so it is time to set up the root sequence.

```
> aa.seq <- AminoAcidSequence(length = 60)
```

Now we will create a pattern of processes. The "left linker", "core" and "right linker" regions evolve by different sets of processes. The core region has no indel processes attached, so its length will remain constant:

```
> process.pattern <- c(rep(list(list(wag, cont.del, cont.ins.wag)), times = 20),
+     rep(list(list(jtt)), times = 20), rep(list(list(lg, cont.del, cont.ins.lg)),
+         times = 20))
```

Apply the process pattern to the root sequence:

```
> aa.seq$processes <- process.pattern
```

Set up site specific rates by iterating over sites and sampling rates from a substitution process specific distribution:

```
> for (i in 1:aa.seq$length) {
+     if (isAttached(aa.seq$sites[[i]], jtt)) {
+         setRateMultipliers(aa.seq, jtt, qnorm(runif(1, min = 0.5, max = 1),
+             mean = 0.001, sd = 0.01), index = i)
+     }
+     else if (isAttached(aa.seq$sites[[i]], wag)) {
+         plusInvGamma(aa.seq, process = wag, pinv = 0.1, shape = 1, index = i)
+     }
+     else if (isAttached(aa.seq$sites[[i]], lg)) {
+         plusInvGamma(aa.seq, process = lg, pinv = 0.1, shape = 1, index = i)
+     }
+ }
```

Sample the states of the root sequence from the attached substitution processes:

```
> sampleStates(aa.seq)
> print(aa.seq)
```

```
[1] "FTMASGEASLGVGQIGSGWLHLPEDRPTPFNLDQVETGETVNTCAVEFKKRGSSLLRIMW"
```

Plot the total rates of the sites:

```
> plot(aa.seq)
```

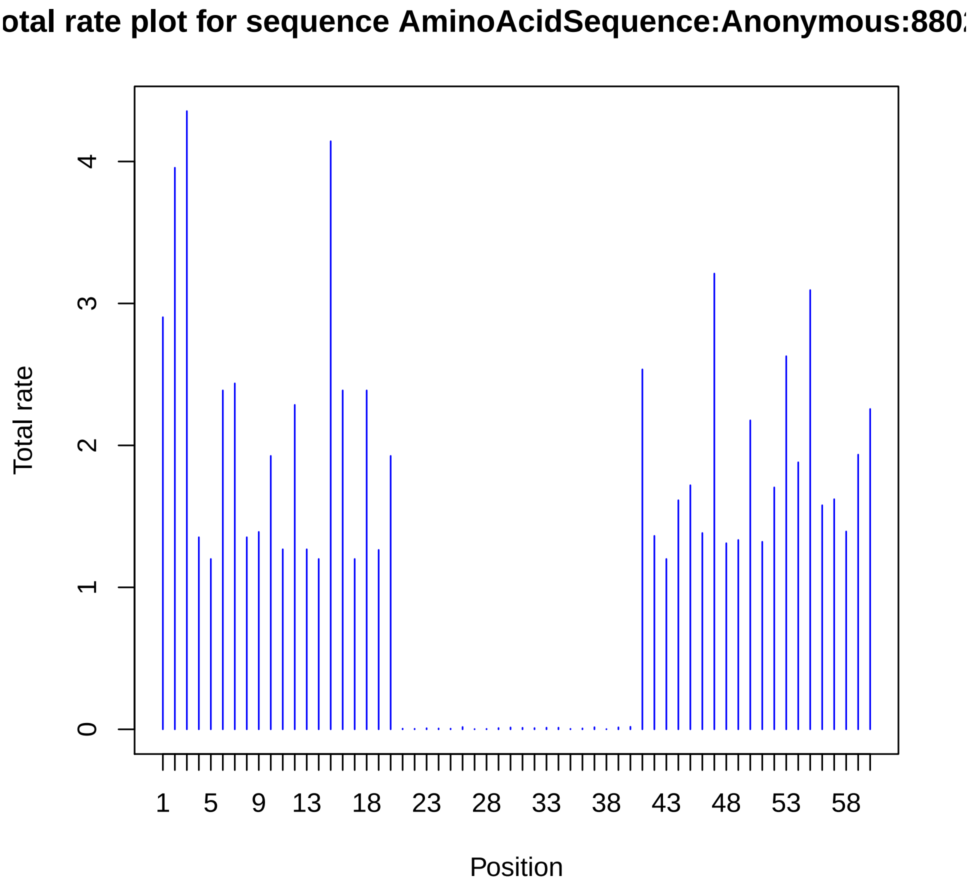

Read in a tree using the ape package:

```
> tree <- read.tree(file = "smalldemotree.nwk")
```

Construct the simulation object and get an object summary:

```
> sim <- PhyloSim(phylo = tree, root.seq = aa.seq)
> summary(sim)
```

```
Name: Anonymous
Id: PhyloSim:Anonymous:92384336
Root Sequence big rate: 80.0215840053019
Tree length: 1.4997
Phylo object details:
        Phylogenetic tree with 5 tips and 4 internal nodes.
        Tip labels:
        [1] "t2" "t4" "t3" "t5" "t1"
        Rooted; includes branch lengths.
Alignment: undefined
Log file: PhyloSim_Anonymous_92384336.log
Log level: -1
```

Plot the simulation object (tree with node labels):

```
> plot(sim)
```

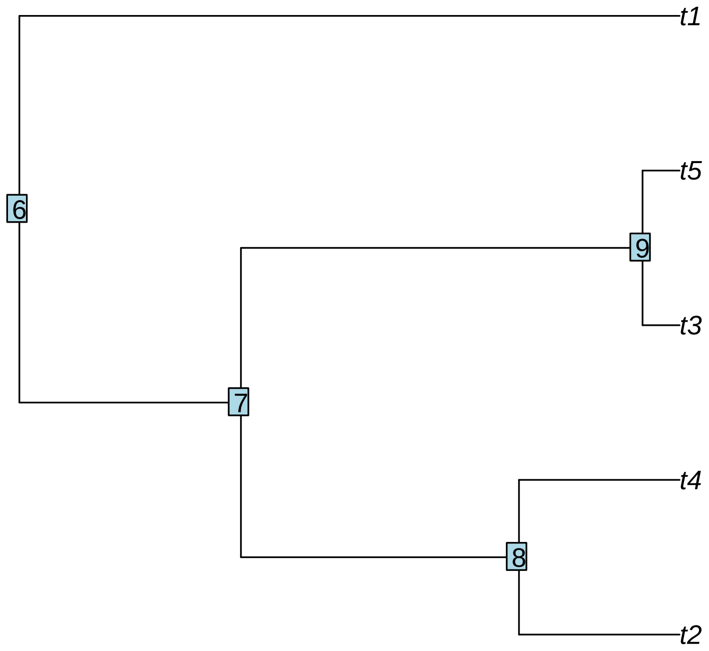

A “node hook” is a function which accepts a `Sequence` object through the named argument “seq” and returns a `Sequence` object. After simulating the branch leading to the node, the resulting `Sequence` object is passed to the node hook and the returned object is used to simulate the downstream branches.

Create a node hook function:

```
> node.hook <- function(seq) {
+     for (site in seq$sites) {
+         if (isAttached(site, jtt)) {
+             attachProcess(site, pam)
+         }
+     }
+     return(seq)
+ }
```

Attach the hook to node 8:

```
> attachHookToNode(sim, node = 8, fun = node.hook)
```

This `node.hook` function will attach the `pam` substitution process to all sites which have the `jtt` process attached (the “core” region). The affected sites will evolve with a doubled rate by a combination of substitution processes (`jtt` and `pam`) in the clade defined by the node - `(t4, t2)`.

Run the simulation:

```
> Simulate(sim)
```

```
!!!!!!!!!!!!!!!!!!!!!!!!!!!!!!!!!!!!!!!!!!!!!!!!!!!!!!!!!!!!!!!!!!!!!!!!!!!!!!!!!!
!! WARNING: fast & careless mode is on, most of the error checking is omitted!  !!
!!    Please note that this also disables the saving of branch statistics.      !!
!!       You can go back to normal mode by deleting the PSIM_FAST object.       !!
!!!!!!!!!!!!!!!!!!!!!!!!!!!!!!!!!!!!!!!!!!!!!!!!!!!!!!!!!!!!!!!!!!!!!!!!!!!!!!!!!!
Simulating edge 1 of 8
Simulating edge 2 of 8
Simulating edge 3 of 8
Simulating edge 4 of 8
Simulating edge 5 of 8
Simulating edge 6 of 8
Simulating edge 7 of 8
Simulating edge 8 of 8
```

Save the resulting alignment, omitting the internal nodes:

```
> saveAlignment(sim, file = "example_V3.1_aln.fas", skip.internal = TRUE)
```

Plot the resulting alignment alongside the tree (including sequences at internal nodes):

```
> plot(sim, num.pages = 1)
```

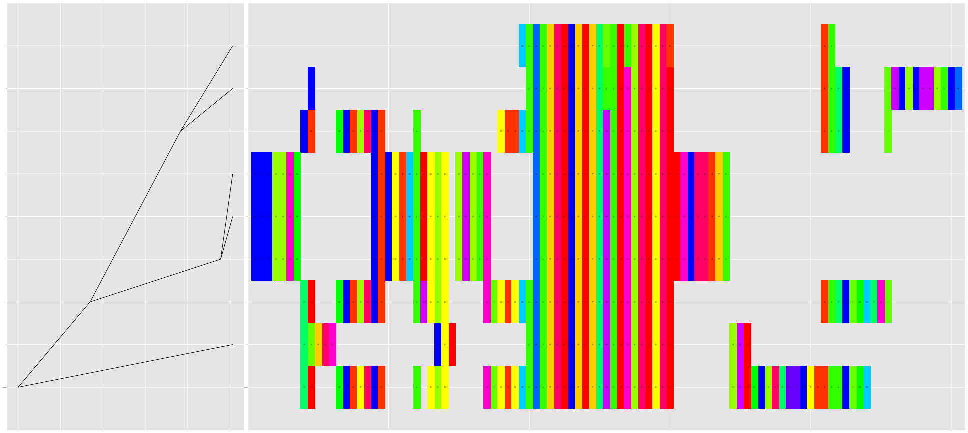

Disable fast mode:

```
> rm(PSIM_FAST)
```

3.2 Evolving codon sequences

Enable "fast & careless" mode:

```
> PSIM_FAST <- TRUE
```

Construct a GY94 codon substitution model:

```
> p <- GY94()
```

Set the transition/transversion rate ratio:

```
> p$kappa = 2
```

Sample codon frequencies from a normal distribution:

```
> codon.freqs <- abs(rnorm(61, mean = 10, sd = 3))
> codon.freqs <- codon.freqs/sum(codon.freqs)
> p$equDist <- codon.freqs
```

Get object summary for p:

```
> summary(p)
```

```
Name: Anonymous
Id: GY94:Anonymous:124141776
Kappa: 2
Genetic code table id: 1
Alphabet:
  Type: Standard
  Symbols: AAA AAC AAG AAT ACA ACC ACG ACT AGA AGC AGG AGT ATA ATC ATG ATT CAA CAC CAG CAT CCA CCC C[illegible]
Unscaled rate matrix: not shown
Equilibrium distribution:
                 AAA        AAC        AAG         AAT        ACA        ACC        ACG
        Prob: 0.01954852 0.01615549 0.01672801 0.006656342 0.01925286 0.01840825 0.01793072
                 ACT        AGA        AGC        AGG       AGT        ATA        ATC
        Prob: 0.01151069 0.01371567 0.02545449 0.01059881 0.0132686 0.01442035 0.02540088
                  ATG         ATT        CAA        CAC        CAG        CAT        CCA
        Prob: 0.007813825 0.008576484 0.01349687 0.01921833 0.02591199 0.01356071 0.01825548
                 CCC        CCG        CCT        CGA         CGC        CGG        CGT
        Prob: 0.01842626 0.01636962 0.01405416 0.01588395 0.009946565 0.01243065 0.01235542
                 CTA        CTC        CTG        CTT        GAA        GAC        GAG
        Prob: 0.02137131 0.02093444 0.01709401 0.02369031 0.01943562 0.01345660 0.01577984
                 GAT        GCA        GCC        GCG        GCT        GGA         GGC
        Prob: 0.01767371 0.01037235 0.02049328 0.02901719 0.01913906 0.01791923 0.006911534
                 GGG        GGT        GTA        GTC        GTG        GTT        TAC
        Prob: 0.01822488 0.01346546 0.01659514 0.01072481 0.01035895 0.02669698 0.01594463
                 TAT        TCA        TCC        TCG        TCT        TGC        TGG
        Prob: 0.01679651 0.01624917 0.01485264 0.02069693 0.02039377 0.01597828 0.01720902
                 TGT        TTA        TTC        TTG        TTT
        Prob: 0.01057747 0.02043037 0.02134118 0.01254363 0.01228169

Site specific parameters (2):
  Id: rate.multiplier
  Name: Rate multiplier
  Type: numeric
  Default value: 1

  Id: omega
  Name: Omega
  Type: numeric
  Default value: 1
```

Get a bubble plot of p:

```
> plot(p, scale = 0.5)
```

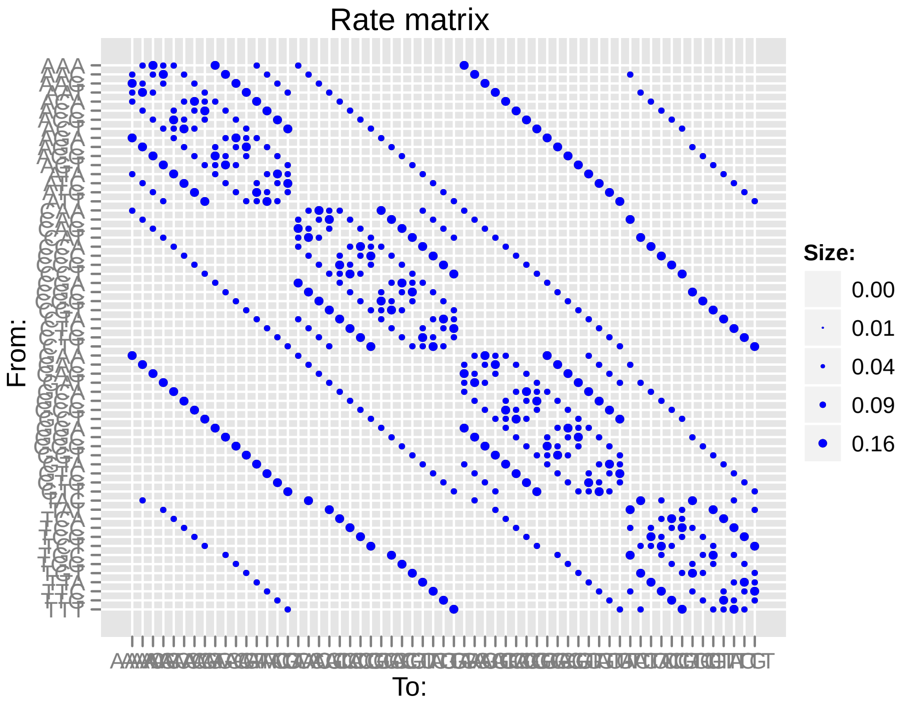

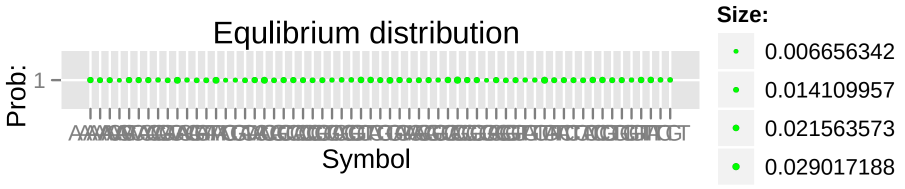

Construct a discrete deletor process:

```
> d <- DiscreteDeletor(rate = 1, sizes = 1:4, probs = c(4, 3, 2, 1)/10)
```

Construct a discrete insertor process inserting neutrally evolving sites:

```
> i <- DiscreteInsertor(rate = 1.5, sizes = 1:4, probs = c(4, 3, 2, 1)/10,
+     template.seq = CodonSequence(length = 4, processes = list(list(p))))
```

Construct root sequence and attach process p:

```
> s <- CodonSequence(length = 30, processes = list(list(p)))
```

Sample omegas from a discrete model:

```
> omegaVarM3(s, p, omegas = c(0, 0.5, 1), probs = c(2/4, 1/4, 1/4))
```

Plot the omega values across sites:

```
> plotParametersAtSites(s, p, "omega")
```

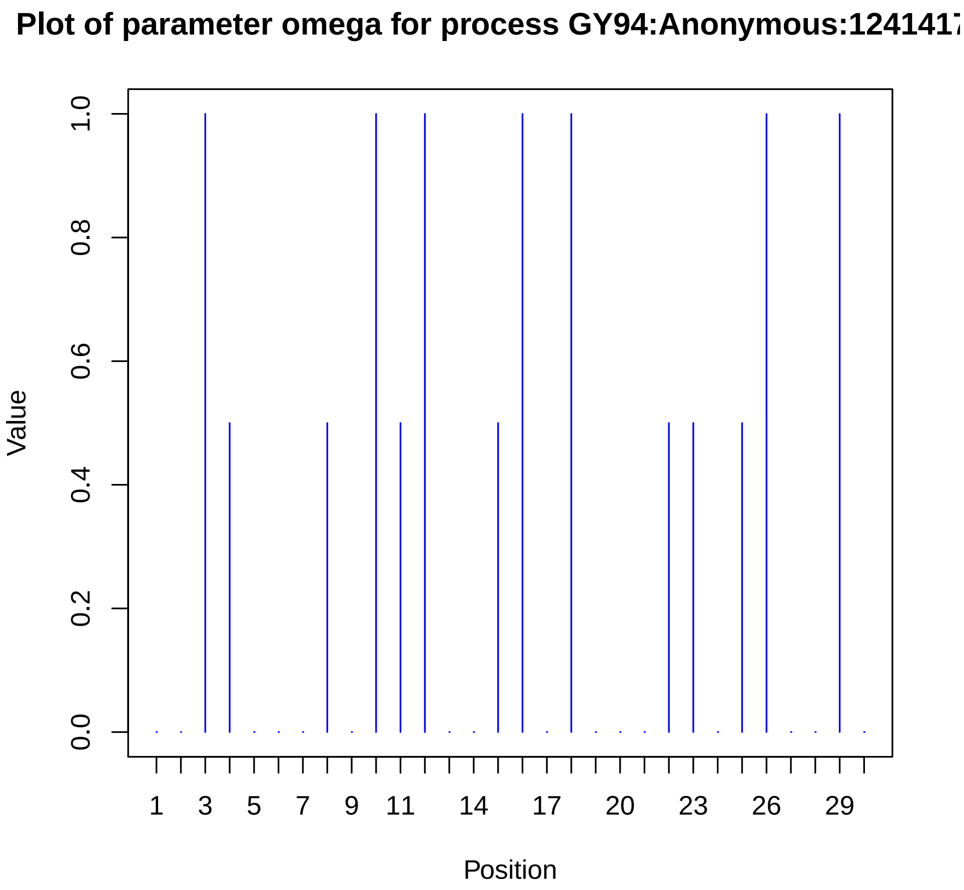

Sample states:

```
> sampleStates(s)
```

Construct the simulation object:

```
> sim <- PhyloSim(root.seq = s, phylo = read.tree("smalldemotree.nwk"))
```

Create a node hook function and attach to node 8:

```
> node.hook <- function(seq) {
+     setOmegas(seq, p, 1)
+     attachProcess(seq, d)
+     attachProcess(seq, i)
+     return(seq)
+ }
> attachHookToNode(sim, node = 8, fun = node.hook)
```

The `node.hook` function sets all omegas to 1 and attaches the insertion process `i` and deletion process `d`. Hence the sequences will evolve neutrally with indels in the clade defined by node 8 - `(t4, t2)`.

Disable fast mode just before simulation in order to preserve branch statistics:

```
> rm(PSIM_FAST)
```

Run the simulation:

```
> Simulate(sim)
```

```
Simulating edge 1 of 8
Simulating edge 2 of 8
Simulating edge 3 of 8
Simulating edge 4 of 8
Simulating edge 5 of 8
Simulating edge 6 of 8
Simulating edge 7 of 8
Simulating edge 8 of 8
```

Plot the resulting alignment alongside the tree:

```
> plot(sim, num.pages = 1)
```

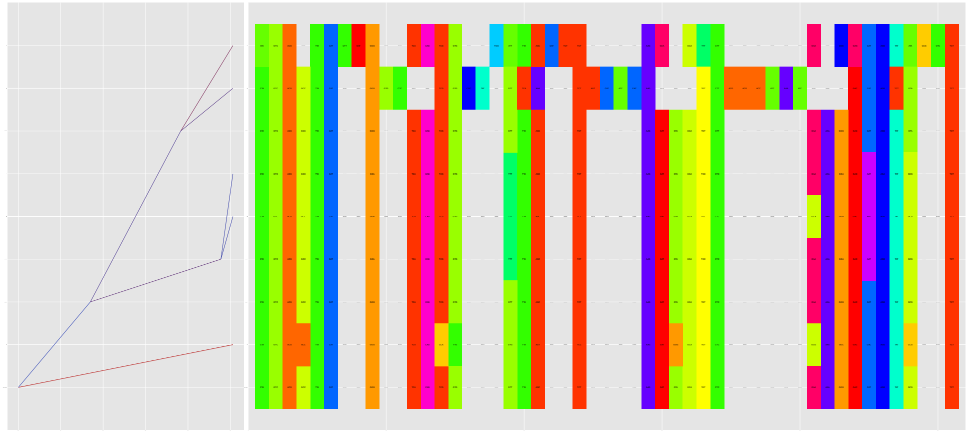

Export the nonsynonymous substitution counts as a phylo object:

```
> nsyn.subst <- exportStatTree(sim, "nr.nsyn.subst")
```

Plot the exported phylo object:

```
> plot(nsyn.subst)
> nodelabels()
```

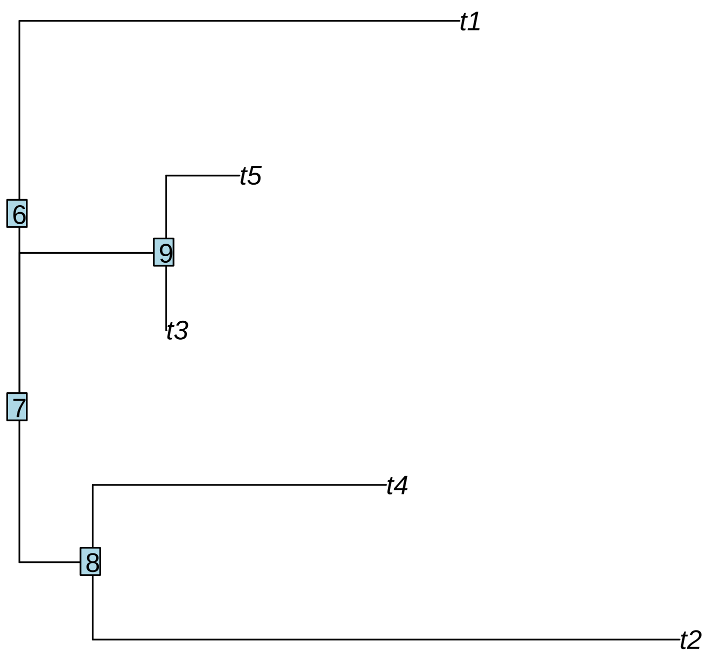

Save the resulting alignment:

```
> saveAlignment(sim, file = "example_V3.2_aln.fas", )
```

3.3 Implementing a new process

The following code demonstrates how to implement a process which performs inverted duplications. In this example we simply replace the function object stored in the `generateBy` virtual field of a `GeneralInsertor` object. Alternatively, we could have defined a new class and set the insert generating function in the constructor method.

Enable fast & careless mode:

```
> PSIM_FAST <- TRUE
```

Construct a DiscreteInsertor process:

```
> ivd <- DiscreteInsertor(rate = 0.06, sizes = c(4, 6), probs = c(2/3,
+     1/3))
```

Set template sequence just to make the process object happy:

```
> ivd$templateSeq <- NucleotideSequence(length = 1)
```

Replace the function object stored in the `generateBy` virtual field. See the documentation of the `GeneralInsertor` class:

```
> ivd$generateBy <- function(process = NA, length = NA, target.seq = NA,
+     event.pos = NA, insert.pos = NA) {
+     target.length <- target.seq$length
+     positions <- (insert.pos + 1):(insert.pos + length)
+     positions <- positions[positions > 0 & positions <= target.length]
+     insert <- copySubSequence(target.seq, positions, process)
+     revComp.NucleotideSequence(insert)
+     setRateMultipliers(insert, ivd, 0)
+     return(insert)
+ }
```

Now we have a process which performs inverted duplications.

Construct a JC69 process object:

```
> p <- JC69()
```

Construct root sequence object:

```
> s <- NucleotideSequence(length = 50)
```

Attach processes via virtual field:

```
> s$processes <- list(list(p, ivd))
```

Sample states from the equilibrium distribution of the attached processes:

```
> sampleStates(s)
```

Detach the substitution process:

```
> detachProcess(s, p)
```

Create among-sites rate variation for the inverted duplication process by sampling rate multipliers from an I+G model:

```
> plusInvGamma(s, ivd, pinv = 0.7, shape = 0.5)
```

Construct simulation object:

```
> sim <- PhyloSim(root.seq = s, phylo = read.tree("smalldemotree.nwk"))
```

Run simulation:

```
> Simulate(sim)
```

```
!!!!!!!!!!!!!!!!!!!!!!!!!!!!!!!!!!!!!!!!!!!!!!!!!!!!!!!!!!!!!!!!!!!!!!!!!!!!!!!
!! WARNING: fast & careless mode is on, most of the error checking is omitted!  !!
!!    Please note that this also disables the saving of branch statistics.      !!
```

```
!!       You can go back to normal mode by deleting the PSIM_FAST object.       !!
!!!!!!!!!!!!!!!!!!!!!!!!!!!!!!!!!!!!!!!!!!!!!!!!!!!!!!!!!!!!!!!!!!!!!!!!!!!!!!!!
Simulating edge 1 of 8
Simulating edge 2 of 8
Simulating edge 3 of 8
Simulating edge 4 of 8
Simulating edge 5 of 8
Simulating edge 6 of 8
Simulating edge 7 of 8
Simulating edge 8 of 8
```

Plot tree and alignment:

```
> plot(sim, num.pages = 1)
```

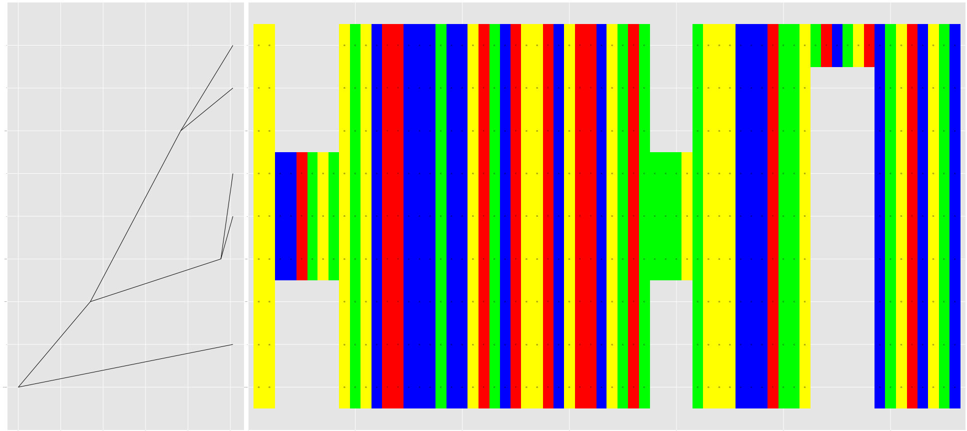

Save alignment:

```
> saveAlignment(sim, file = "example_V3.3.fas")
```

Disable fast & careless mode:

```
> rm(PSIM_FAST)
```

3.4 Evolving a genomic region containing a "gene"

The following code demonstrates how to simulate a genomic region containing a gene with introns and noncoding regions.

We will simulate the following features:

- NC1 (length: 1000 nucleotide sites) - noncoding region 1, evolving under a GTR substitution model with deletions and insertions (of Cs only).
- E1 (length: 400 codon sites) - exon 1, evolving under a nucleotide-scaled GY94 codon model with small indels.
- I1-5 (length: 200 nucleotide sites) - introns 1-5, evoling under a F84 substitution model with indels
- E2-5 (length: 200 codon sites) - exons 2-5, evolving under a nucleotide-scaled GY94 codon model.

- E6 (length: 400 codon sites) - exon 6, evolving under a nucleotide-scaled GY94 codon model with small indels.
- NC2 (length: 1400 nucleotide sites) - noncoding region 2, evolving under a K80 substitution model.
- Invariable start codon and splice sites.
- A special substitution process for stop codons.

The total length of the Sequence object is 5000. We will also simulate heterogeneity of nucleotide substitution rates, indel rates and omega ratios.

Enable fast & careless mode:

```
> PSIM_FAST <- TRUE
```

Create a list holding partition information and set partition properties:

```
> part <- list()
```

Partition NC1:

```
> part$nc1 <- list(type = "noncoding", len = 1000, subst = NA, ins = NA,
+     del = NA, gamma.shape = 0.5)
> part$nc1$subst <- GTR(rate.params = list(a = 1, b = 2, c = 3, d = 1,
+     e = 2, f = 3), base.freqs = c(1.5, 1, 1.5, 1)/5)
> part$nc1$ins <- DiscreteInsertor(rate = 0.025, sizes = 1:6, probs = 6:1/21,
+     template.seq = NucleotideSequence(string = "CCCCCC"))
> part$nc1$del <- DiscreteDeletor(rate = 0.025, sizes = 1:6, probs = 6:1/21)
```

Partition E1:

```
> part$e1 <- list(type = "first.exon", len = 400, subst = NA, ins = NA,
+     del = NA, omegas = c(0, 1, 1.1), omega.probs = c(4, 2, 1)/5)
```

Construct a "nucleotide-scaled" codon model which can be used in mixed sequences:

```
> part$e1$subst <- GY94(kappa = 2, scale.nuc = TRUE)
> part$e1$ins <- DiscreteInsertor(rate = 0.025, sizes = 1:2, probs = c(2/3,
+     1/3), template.seq = CodonSequence(length = 2, processes = list(list(part$e1$subst))))
> part$e1$del <- DiscreteDeletor(rate = 0.025, sizes = 1:2, probs = c(2/3,
+     1/3))
> f84 <- F84()
> f84$kappa <- 1.8
> gy94.e2_5 <- clone(part$e1$subst)
> gy94.e2_5$kappa <- 1.5
```

Partitions E2-5 and I1-4:

```
> del.introns <- DiscreteDeletor(rate = 0.025, sizes = 1:8, probs = 8:1/36)
> ins.introns <- DiscreteInsertor(rate = 0.025, sizes = 1:8, probs = 8:1/36,
+     template.seq = NucleotideSequence(length = 8, processes = list(list(f84))))
> for (i in 1:4) {
+     part[[paste("i", i, sep = "")]] <- list(type = "intron", len = 200,
```

```
+         subst = f84, ins = ins.introns, del = del.introns, gamma.shape = abs(rnorm(1,
+             mean = 1, sd = 0.2)))
+     part[[paste("e", i + 1, sep = "")]] <- list(type = "exon", len = 200,
+         subst = gy94.e2_5, ins = NA, del = NA, omegas = c(0, 0.5), omega.probs = c(3/4,
+             1/4))
+ }
```

Partition I5:

```
> part$i5 <- list(type = "intron", len = 200, subst = f84, ins = ins.introns,
+     del = del.introns, gamma.shape = abs(rnorm(1, mean = 1, sd = 0.1)))
> rm(f84, gy94.e2_5, del.introns, ins.introns)
```

Partition E6:

```
> part$e6 <- list(type = "last.exon", len = 400, subst = NA, ins = NA,
+     del = NA, omegas = c(0, 1, 1.1), omega.probs = c(4, 2, 1)/5)
> tmp <- clone(part$e1$subst)
> tmp$kappa <- 0.5
> part$e6$subst <- tmp
> part$e6$del <- DiscreteDeletor(rate = 0.025, sizes = 1:4, probs = c(4,
+     3, 2, 1)/10)
> part$e6$ins <- DiscreteInsertor(rate = 0.025, sizes = 1:2, probs = c(1/3,
+     2/3), template.seq = CodonSequence(length = 2, processes = list(list(part$e6$subst))))
```

Partition NC2:

```
> part$nc2 <- list(type = "noncoding", len = 1400, subst = K80(rate.params = list(Alpha = 2,
+     Beta = 1)), ins = NA, del = NA, gamma.shape = 0.5)
```

Construct root sequence object:

```
> s <- Sequence(length = 5000)
```

Construct Alphabet objects:

```
> nuc <- NucleotideAlphabet()
> cod <- CodonAlphabet()
```

Construct an Alphabet object containing the stop codons:

```
> stop.alphabet <- Alphabet(symbols = c("TAG", "TAA", "TGA"))
```

Construct a substitution process acting on stop codons only:

```
> stop.subst <- GeneralSubstitution(alphabet = stop.alphabet, rate.list = list(`TAG->TAA` = 1,
+     `TAG->TGA` = 2, `TAA->TAG` = 3, `TAA->TGA` = 1, `TGA->TAG` = 2,
+     `TGA->TAA` = 3))
```

Get a bubble plot of `stop.subst`:

```
> plot(stop.subst, scale = 0.5)
```

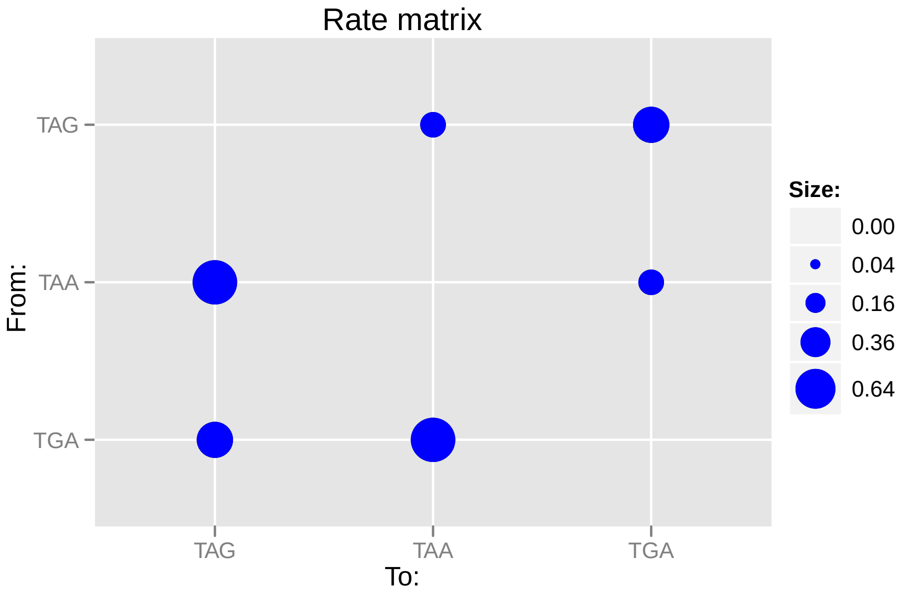

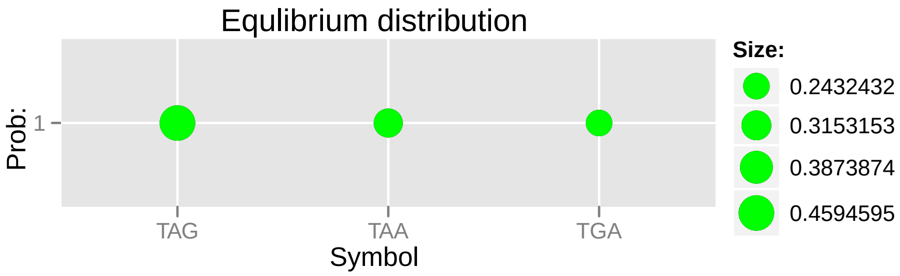

Iterate over partitions, set up processes and rate multipliers, fix start codon and splice sites, attach the `stop.subst` process to stop codon:

```
> pos <- 0
> for (i in part) {
+     beg <- pos + 1
+     end <- pos + i$len
+     range <- beg:end
+     if ((i$type == "noncoding") | (i$type == "intron")) {
+         setAlphabets(s, list(nuc), range)
+     }
+     else {
+         setAlphabets(s, list(cod), range)
+     }
+     if (!is.na(i$del)) {
+         attachProcess(s, i$del, range)
+     }
+     if (!is.na(i$ins)) {
+         attachProcess(s, i$ins, range)
+     }
+     if ((i$type == "noncoding") | (i$type == "intron")) {
+         attachProcess(s, i$subst, range)
+         plusInvGamma(this = s, process = i$subst, pinv = 0.6, shape = i$gamma.shape,
+             range)
+         if (i$type == "intron") {
```

```
+             setStates(s, c("G", "T", "A", "G"), c(beg, beg + 1, end -
+                 1, end))
+             setRateMultipliers(s, i$subst, 0, c(beg, beg + 1, end -
+                 1, end))
+             setInsertionTolerance(s, i$ins, 0, c(beg, beg + 1, end -
+                 1, end))
+             setDeletionTolerance(s, i$del, 0, c(beg, beg + 1, end -
+                 1, end))
+         }
+     }
+     else if (i$type == "exon") {
+         attachProcess(s, i$subst, range)
+         omegaVarM3.CodonSequence(s, i$subst, i$omegas, i$omega.probs,
+             range)
+     }
+     if (i$type == "first.exon") {
+         attachProcess(s, i$subst, range)
+         setStates(s, "ATG", beg)
+         setRateMultipliers(s, i$subst, 0, beg)
+         setInsertionTolerance(s, i$ins, 0, beg)
+         setDeletionTolerance(s, i$del, 0, beg)
+     }
+     if (i$type == "last.exon") {
+         attachProcess(s, i$subst, range)
+         detachProcess(s, i$subst, end)
+         setAlphabets(s, list(stop.alphabet), end)
+         attachProcess(s, stop.subst, end)
+         sampleStates(s, end)
+         setInsertionTolerance(s, i$ins, 0, end)
+         setDeletionTolerance(s, i$del, 0, end)
+     }
+     pos <- end
+ }
```

Sample remaining site states:

```
> sampleStates(s)
```

Generate a random coalescent tree with 3 leaves and construct simulation object:

```
> sim <- PhyloSim(root.seq = s, phylo = rcoal(3))
```

Scale tree length to 0.15:

```
> scaleTree(sim, 0.15/sim$treeLength)
```

Run simulation:

```
> Simulate(sim)
```

```
!!!!!!!!!!!!!!!!!!!!!!!!!!!!!!!!!!!!!!!!!!!!!!!!!!!!!!!!!!!!!!!!!!!!!!!!!!!!!!!
!! WARNING: fast & careless mode is on, most of the error checking is omitted!  !!
!!    Please note that this also disables the saving of branch statistics.      !!
```

```
!!       You can go back to normal mode by deleting the PSIM_FAST object.       !!
!!!!!!!!!!!!!!!!!!!!!!!!!!!!!!!!!!!!!!!!!!!!!!!!!!!!!!!!!!!!!!!!!!!!!!!!!!!!!!!!!!
Simulating edge 1 of 4
Simulating edge 2 of 4
Simulating edge 3 of 4
Simulating edge 4 of 4
```

Plot tree and alignment, omitting ancestral sequences:

```
> plot(sim, num.pages = "auto", plot.ancestors = FALSE)
```

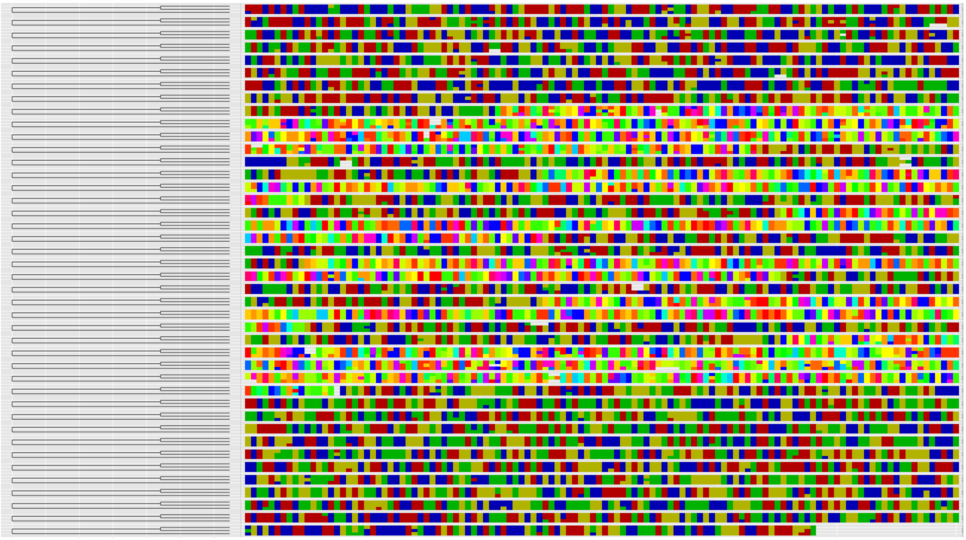

Save alignment:

```
> saveAlignment(sim, file = "example_V3.4.fas")
```

Disable fast & careless mode:

```
> rm(PSIM_FAST)
```

4 Details of the fast field deletion model

A natural way to incorporate deletions into the Gillespie framework is to assign an individual rate to every possible deletion event. Modelling in this manner is extremely general but requires a lot of specification: not only individual sites' tolerance to deletion but also of how they interact with neighbouring sites. Instead we propose a more restricted "field model" of deletion that generalises previous work to allow the rate at which deletions occur to vary across the sequence but only requires one parameter per site – its deletion tolerance – to be specified. Under this model, deletions are proposed in same manner as other events, specifying a rate of occurrence and a distribution of lengths and then assuming that the location and orientation of the deletion is chosen uniformly, but proposed deletions may then be rejected based on sites they propose to remove.

Firstly consider only single-site deletions and let each site, i, in the sequence have an associated deletion tolerance parameter, $d_i \in [0, 1]$, representing the probability that it is actually deleted given that a deletion is proposed. A proposal / acceptance step is equivalent to just proposing at a slower rate, so this is just a special case of the most general model but one that can be implemented efficiently as a single

Gillespie event (a deletion occurred somewhere) rather than a large number of slower events (treating every possible deletion separately). Sites where $d_i = 1$ are deleted at the background rate, sites with $d_i < 1$ are deleted more slowly, and sites with $d_i = 0$ are never deleted. For proposed deletions that span multiple sites, $\mathcal{I}$, each site is considered independently and the proposed deletion is accepted if and only if every site accepts it: the total probability of acceptance is therefore $\prod_{i \in \mathcal{I}} d_i$.

It is natural to think of the background rate of deletion as a neutral rate but this is not necessary and can lead to the Gillespie algorithm becoming inefficient: for example, an extremely deletion intolerant sequence will reject almost all deletions proposed and so waste many steps. Instead we can rescale the process ("fast field deletion model") so that deletions are proposed at a rate equal to what would occur if the entire sequence had a deletion tolerance equal to its most tolerant site (deletion tolerance d) and then accept a deletion spanning sites $\mathcal{I}$ with probability d_i/d. Table 1 gives the rate scaling factor and distribution of deletion lengths after scaling for a variety of distributions that could be used to model deletion length. In most cases, the rescaled distribution of deletion lengths is a member of the same family as the "neutral" process.

Distribution	Density	Scale factor	Rescaled distribution
Geometric, $\mathrm{Geom}(\lambda)$	$\lambda^{k-1}(1-\lambda)$	$d\frac{1-\lambda}{1-d\lambda}$	$\mathrm{Geom}(d\lambda)$
Poisson + 1, $\mathrm{Po}_{+1}(\lambda)$	$\frac{e^{-\lambda}\lambda^{k-1}}{\Gamma(k)}$	$de^{-\lambda(1-d)}$	$\mathrm{Po}_{+1}(d\lambda)$
Conway-Maxwell Poisson + 1, $\mathrm{CMP}_{+1}(\lambda,\nu)$	$\frac{\lambda^{k-1}}{\Gamma(k)^{\nu}\mathcal{Z}(\lambda,\nu)}$	$d\frac{\mathcal{Z}(\lambda,\nu)}{\mathcal{Z}(d\lambda,\nu)}$	$\mathrm{CMP}_{+1}(d\lambda,\nu)$
Negative Binomial + 1, $\mathrm{NB}_{+1}(\lambda,r)$	$\frac{\Gamma(r+k-1)}{\Gamma(k)\Gamma(r)}(1-\lambda)^r\lambda^{k-1}$	$d\frac{(1-\lambda)^r}{(1-d\lambda)^r}$	$\mathrm{NB}_{+1}(d\lambda,r)$

Table 1: The rate scaling factor and distribution of deletion lengths after scaling for a variety of distributions that could be used to model deletion length.
