# Supplementary material for: PhyloSim - Monte Carlo simulation of sequence evolution in the R statistical computing environment
Source: BMC Bioinformatics. 2011 Apr 19;12:104. doi: 10.1186/1471-2105-12-104 (PMC3102636; doi:10.1186/1471-2105-12-104)
Supplement: Additional file 2 — Appendix A. [file 1471-2105-12-104-S2.PDF]

PhyloSim – Monte Carlo simulation of sequence evolution in the R statistical computing environment

Supporting Appendix A

Botond Sipos

November 5, 2010

Contents

1 Overview **2**

2 Results **3**

2.1 Nucleotide sequences 3

2.2 Amino acid sequences 13

2.3 Codon sequences 22

3 Computing time required for the simulations **32**

1 Overview

The validity of the framework has been tested by simulating the evolution of nucleotide, amino acid and codon sequences of increasing length and estimating the value of some parameters and the branch lengths from the resulting alignments with the PAML package.

We simulated the evolution of nucleotide, amino acid, and codon sequences having the following lengths: 10, 50, 100, 500, 1000, 5000, 10000. We ran the simulations with the `PSIM_FAST` mode enabled. We performed 100 independent replications for every sequence length.

We evolved the sequences along the following tree having a total length of 0.8:

```
((t3:0.03981003266,t2:0.1461842029):0.08733799994,(t1:0.1526954196,
(t4:0.1141125426,t5:0.05465443676):0.1164810316):0.08872433394);
```

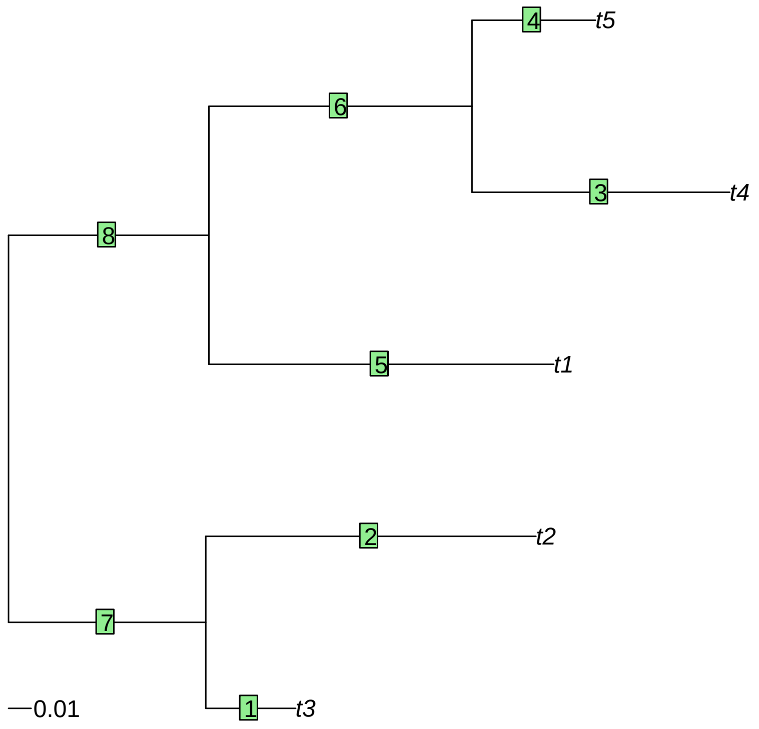

We compared the parameter values estimated from the simulated alignments to the true value of the parameters.

The sequence type specific details and the results are described in the next section. The full simulation code is available at http://github.com/sbotond/phylosim/tree/master/cons_test/.

2 Results

2.1 Nucleotide sequences

We simulated the evolution of nucleotide sequences under the F84+dΓ model (discrete gamma, 8 categories), with a transition/transversion rate ratio parameter $\kappa_{F84} = 1$ and gamma shape parameter $\alpha = 0.25$. We set the base frequencies to: **T** = 0.272 **C** = 0.227 **A** = 0.272 **G** = 0.227.

We estimated the parameters of the F84+dΓ model and the branch lengths from the simulated alignments by using the `baseml` program from the `PAML` package (version 4.4c). See the control file (baseml.ctl) for details.

The following plots summarize the results of the simulations. The x axis represents the sequence length , while the logarithmically transformed y axis shows the estimated value of the parameters (κ_{F84}, α and the branch lengths). The x axis values are jittered for better visiblity. The horizontal red line represents the true value of the parameter.

The overlayed box-and-whisker plots indicate the smallest value, the lower quartile (Q1), the median (Q2), the upper quartile (Q3) and the maximum value.

Kappa (F84)

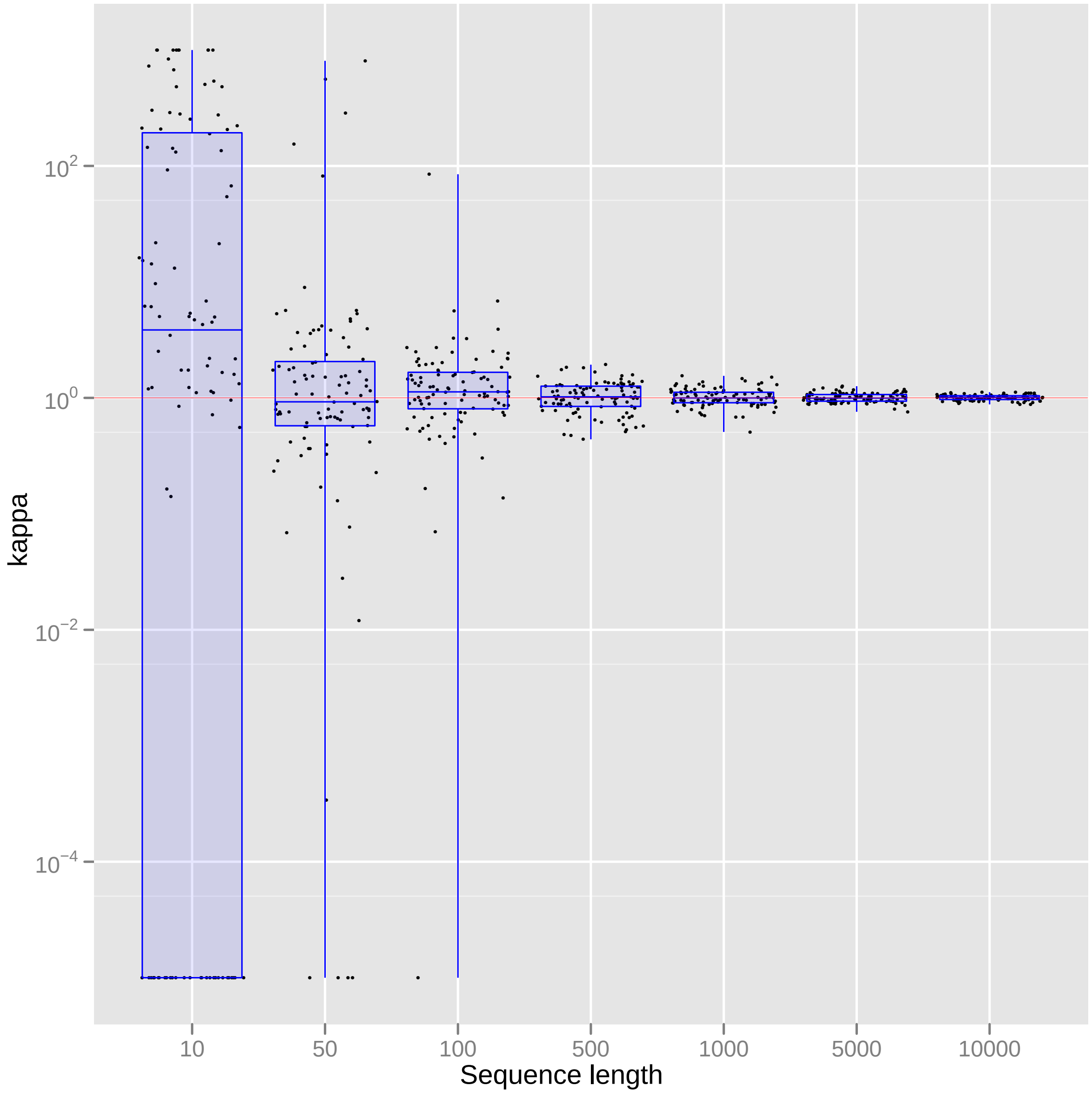

10^2
10^0
10^{-2}
10^{-4}
kappa
10
50
100
500
1000
5000
10000
Sequence length

Alpha

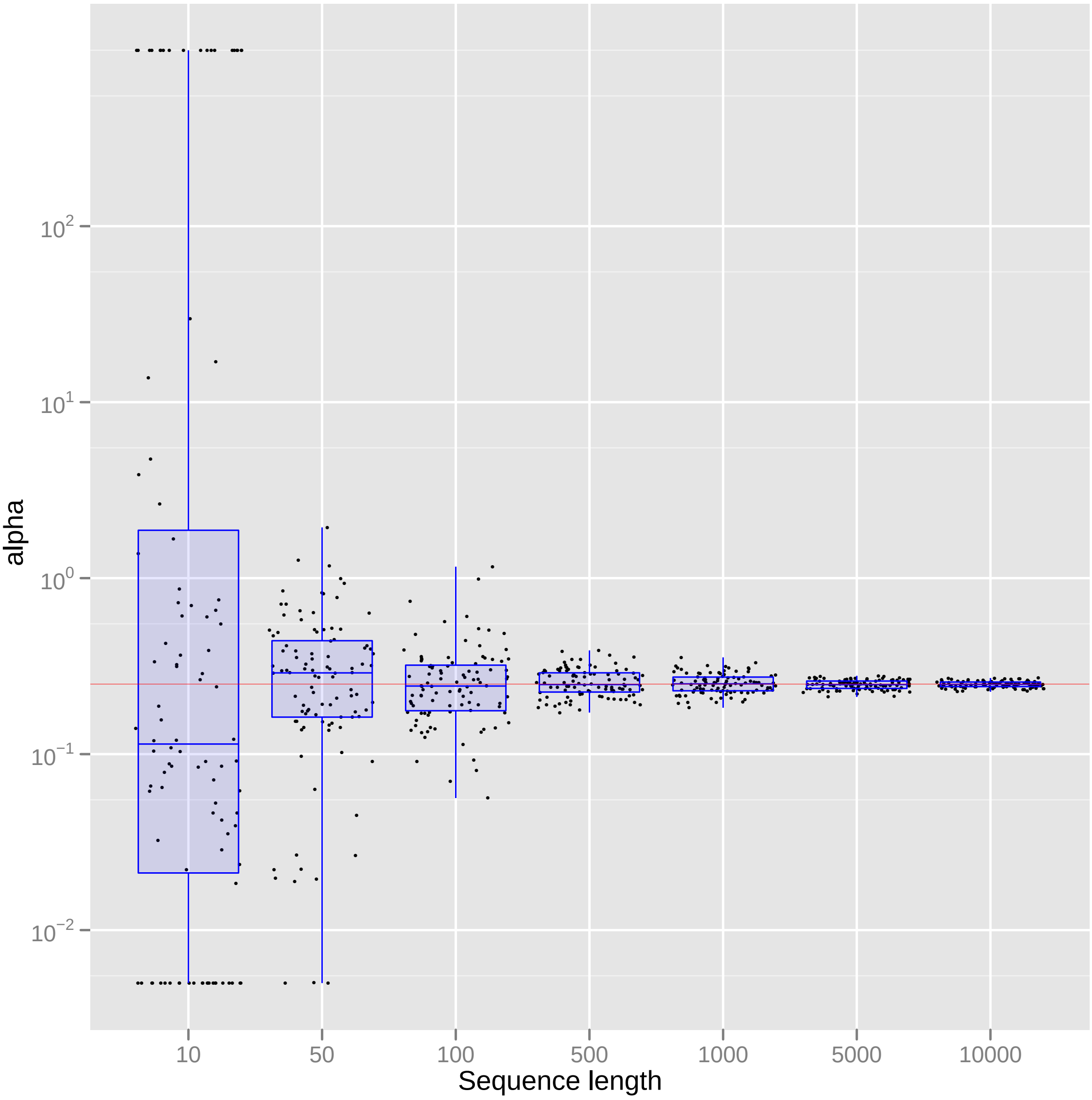

alpha
10^2
10^1
10^0
10^{-1}
10^{-2}
10
50
100
500
1000
5000
10000
Sequence length

Length of branch 1

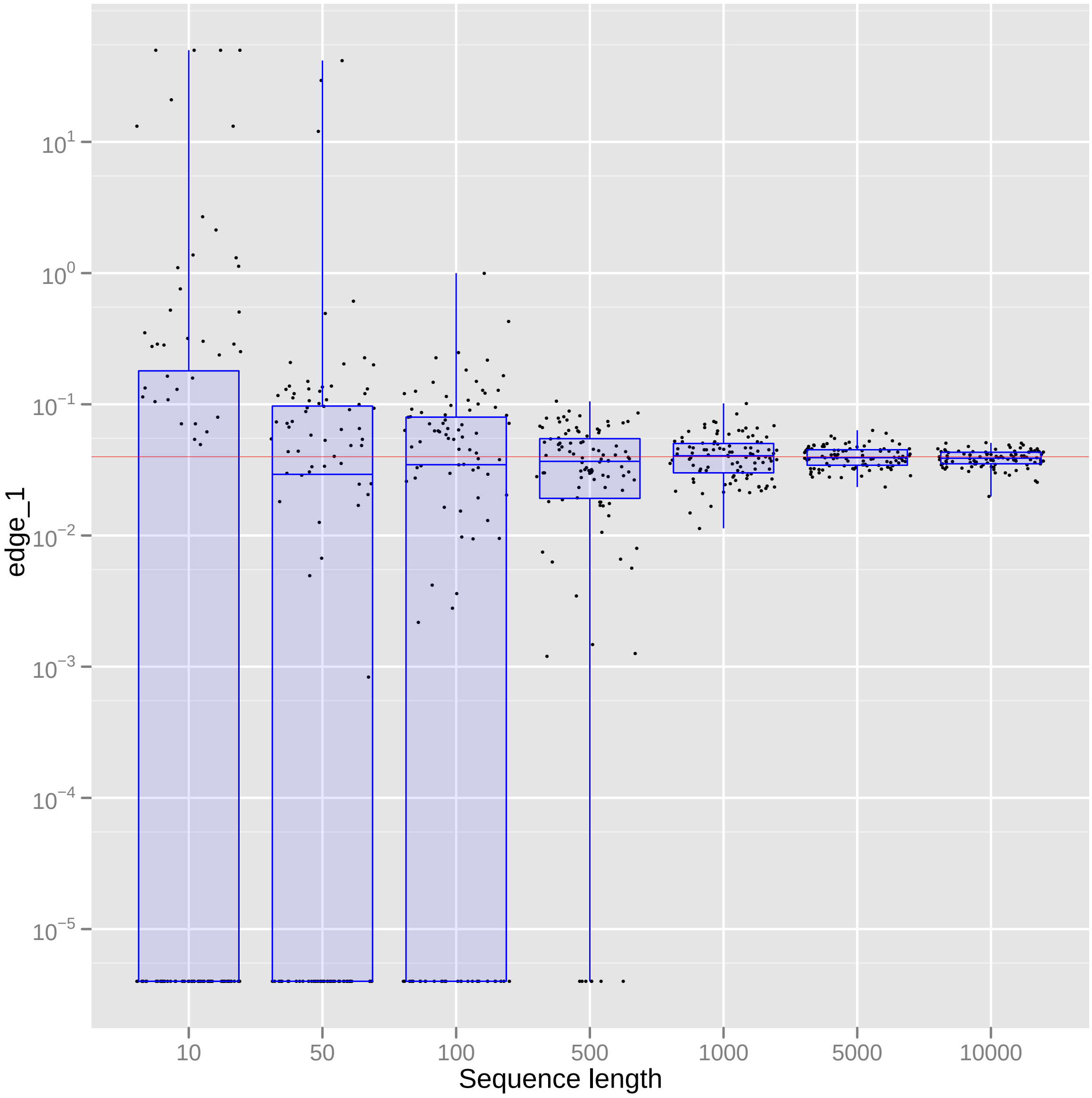

Length of branch 2

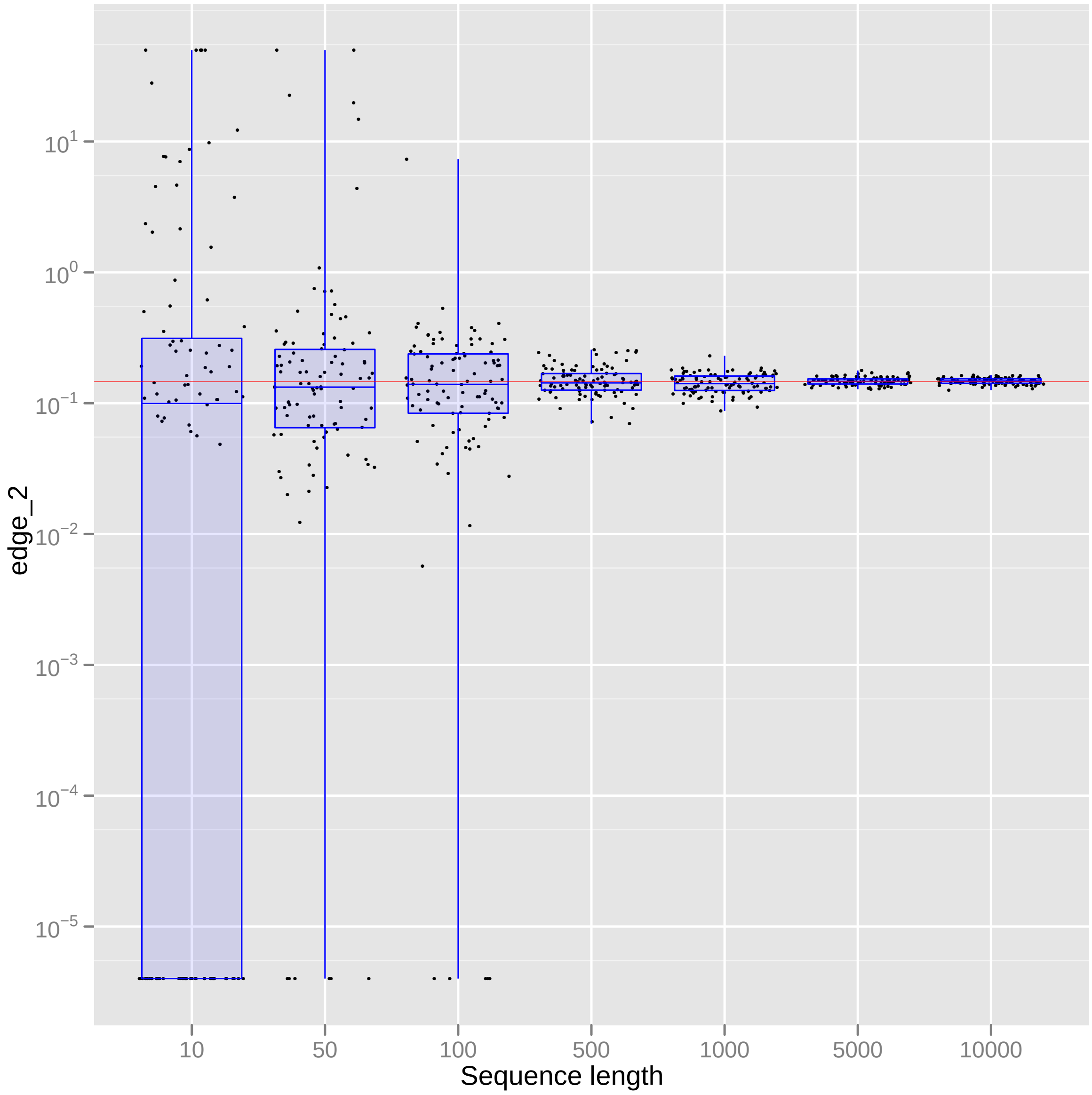

Length of branch 3

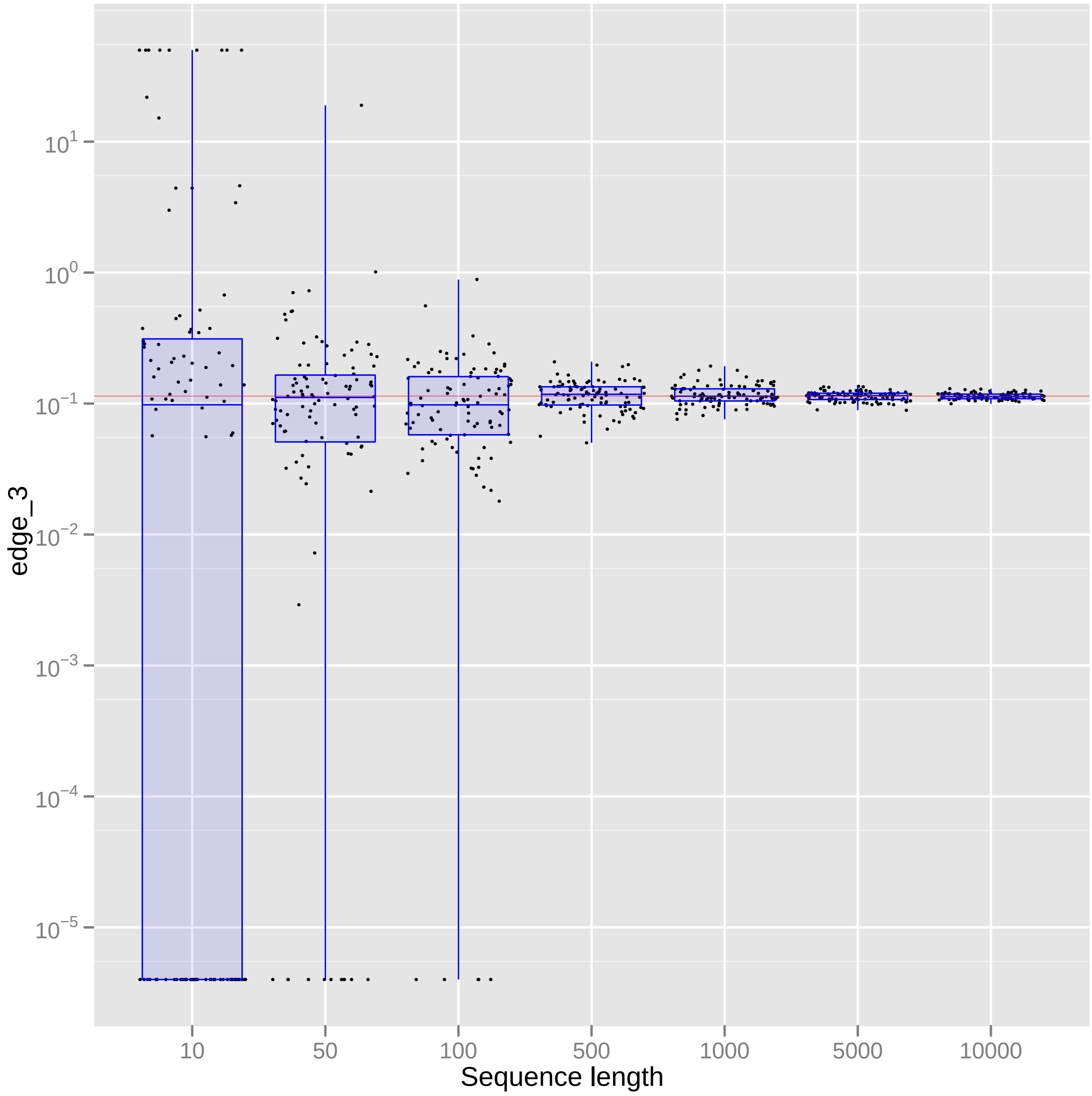

Length of branch 4

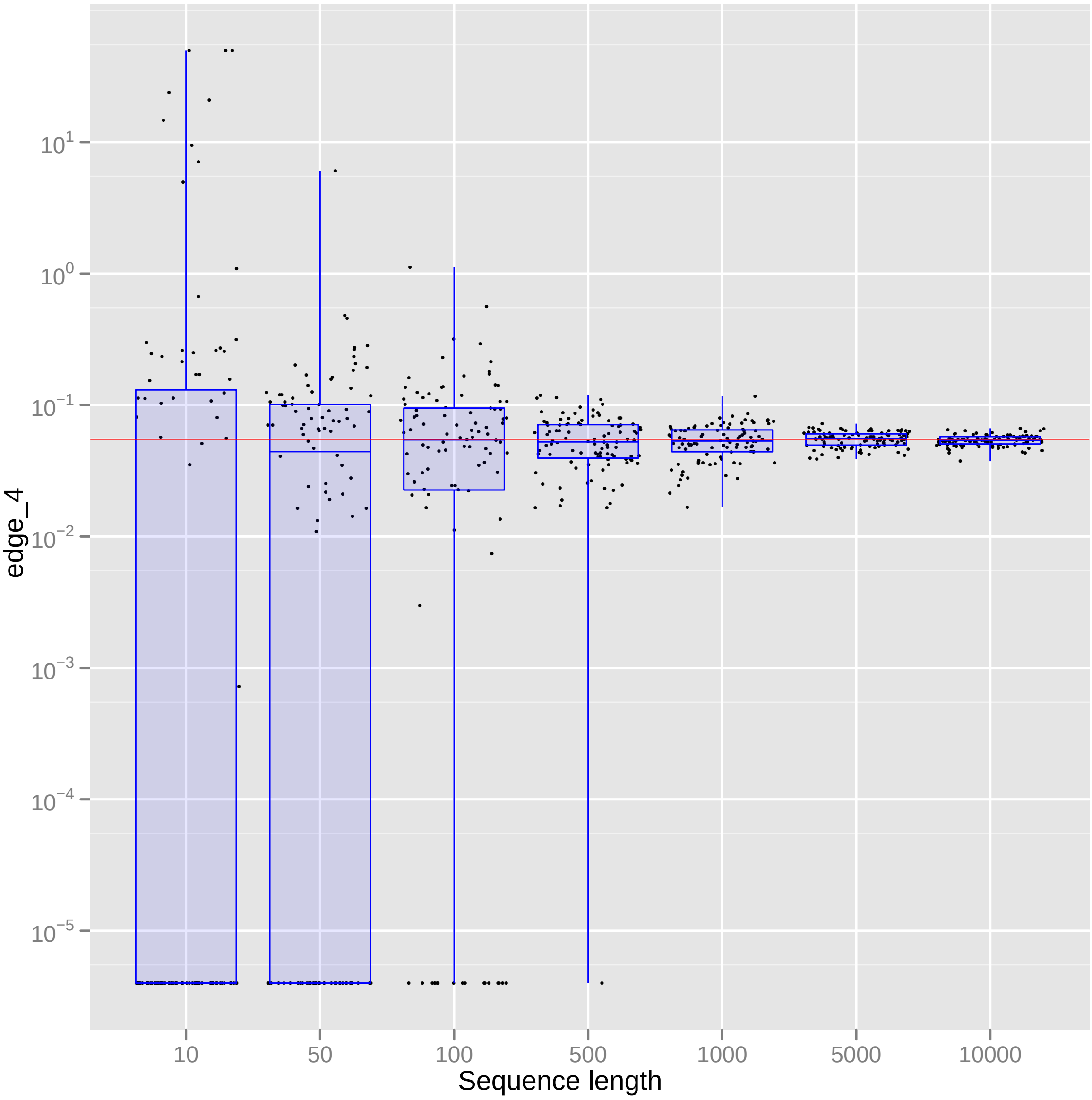

Length of branch 5

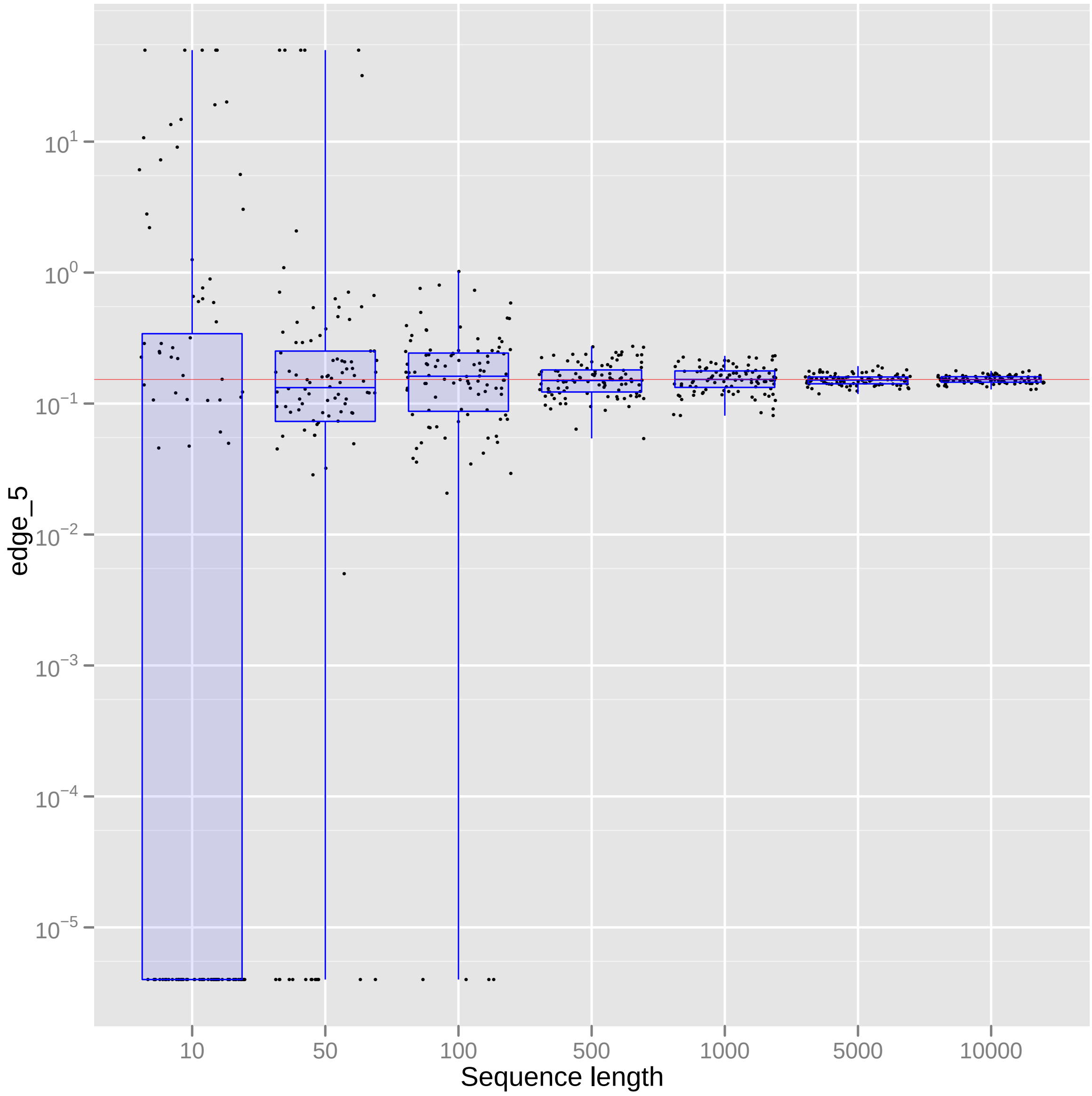

Length of branch 6

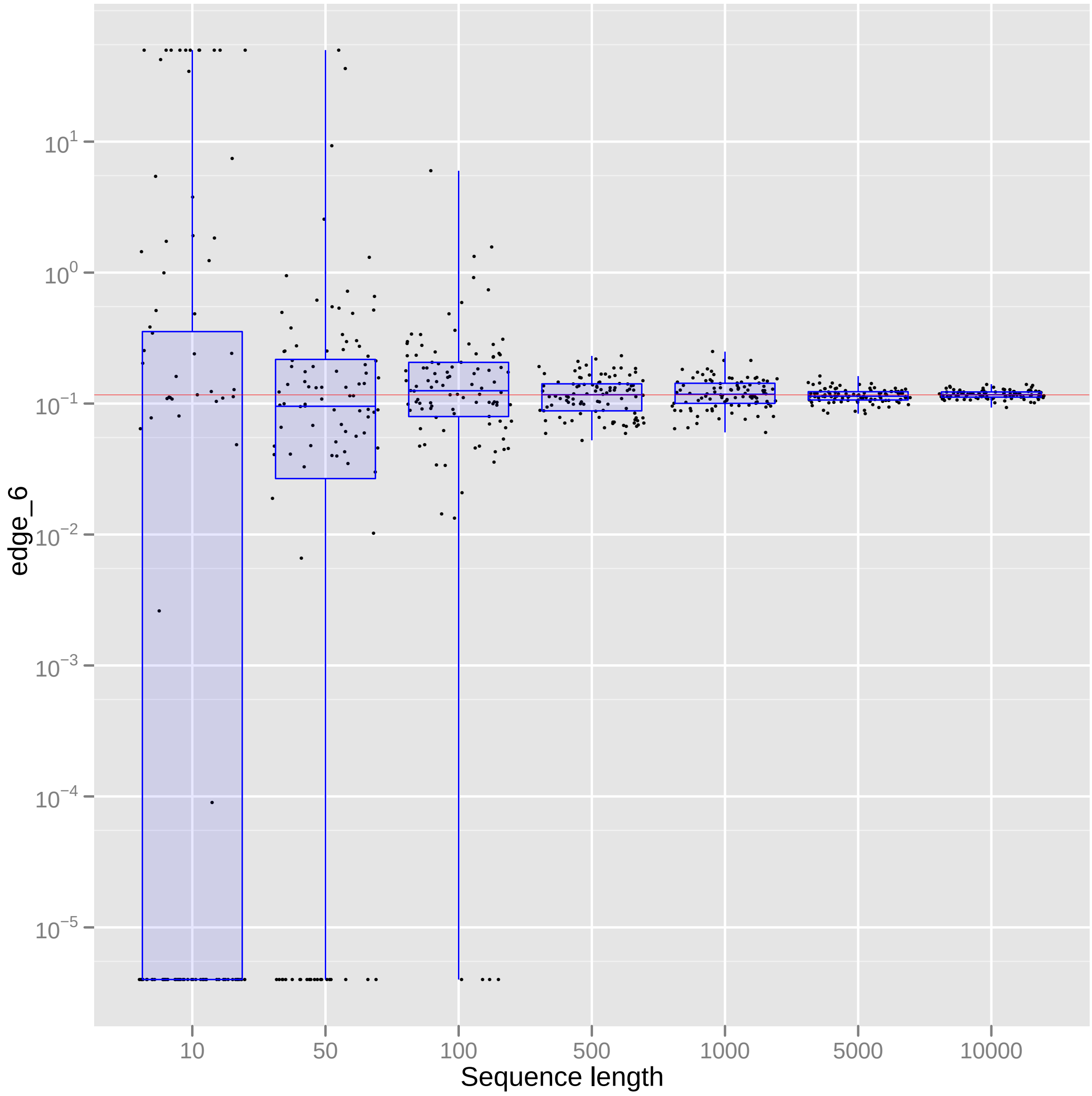

Length of branch 7

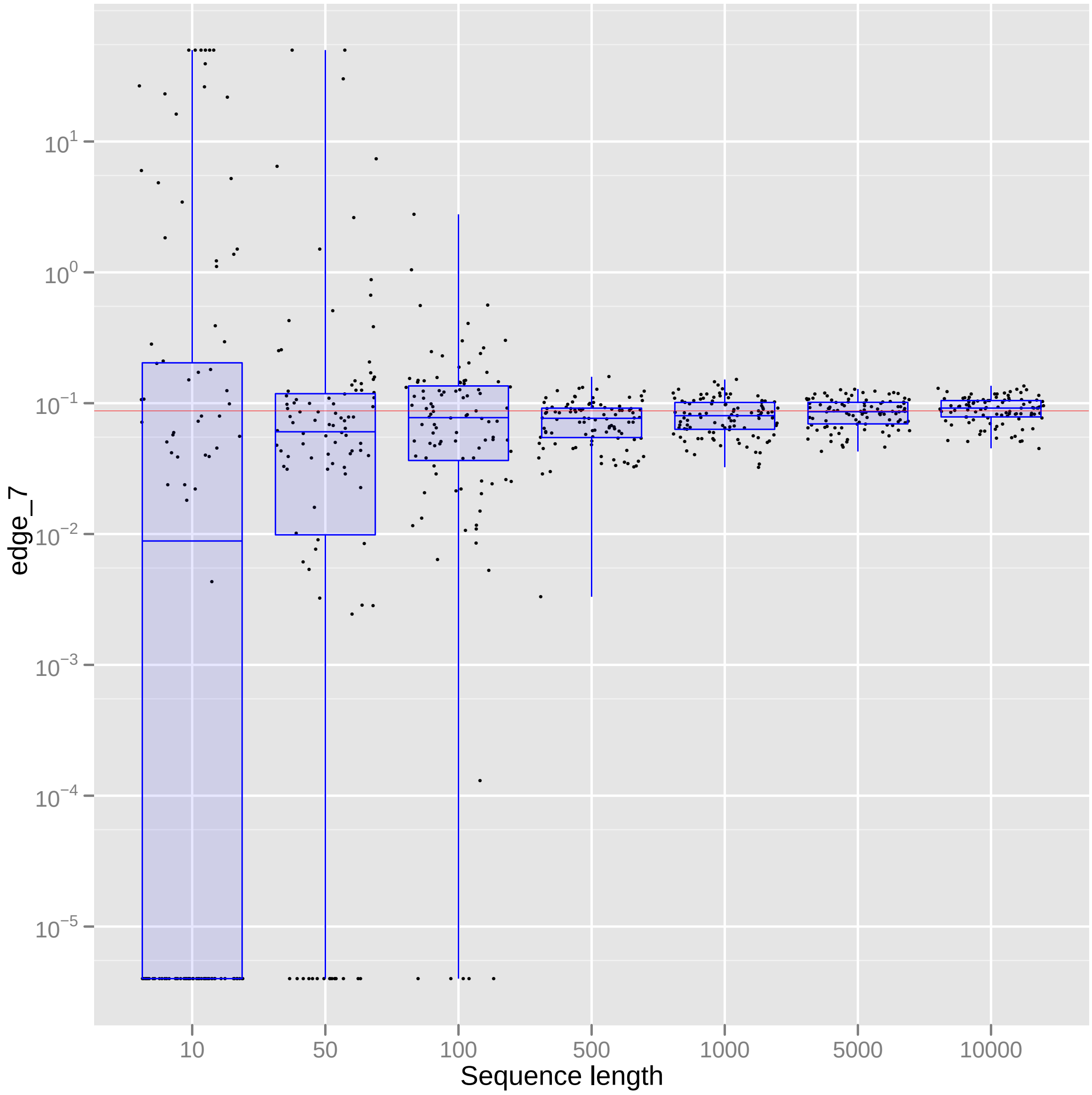

Length of branch 8

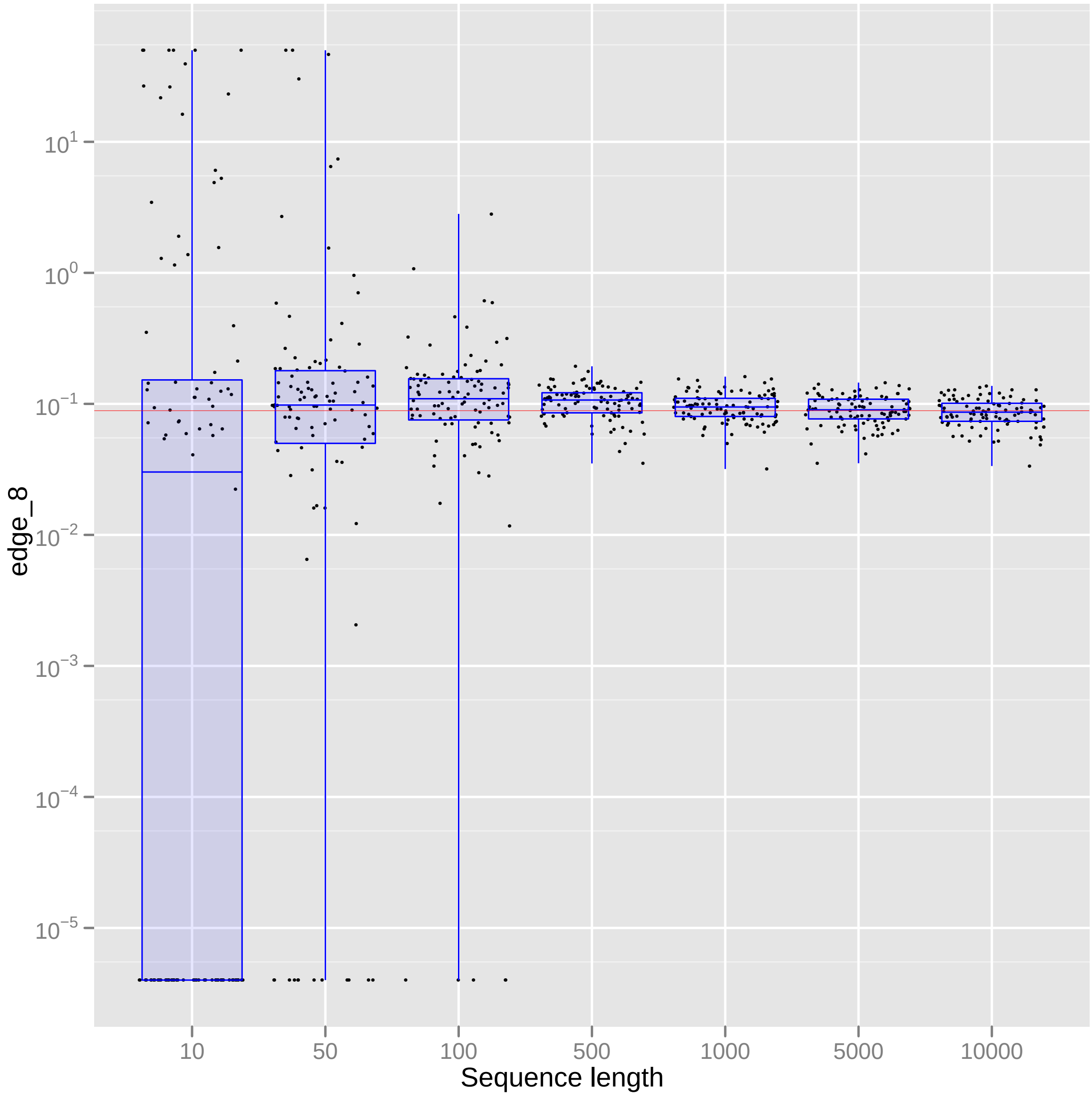

2.2 Amino acid sequences

We simulated the evolution of amino acid sequences under the WAG+dΓ+F model (discrete gamma, 8 categories), with a gamma shape parameter $\alpha = 1$. We used the amino acid frequencies published by Whelan and Goldman (2001, *Mol Biol Evol* 18:691–699).

We estimated the gamma shape parameter (α) and the branch lengths from the simulated alignments by using the `codeml` program from the `PAML` package (version 4.4c). See the control file (codeml_aa.ctl) for details.

The following plots summarize the results of the simulations. The x axis represents the sequence length , while the logarithmically transformed y axis shows the estimated value of the parameters (α and the branch lengths). The x axis values are jittered for better visiblity. The horizontal red line represents the true value of the parameter.

The overlayed box-and-whisker plots indicate the smallest value, the lower quartile (Q1), the median (Q2), the upper quartile (Q3) and the maximum value.

Alpha

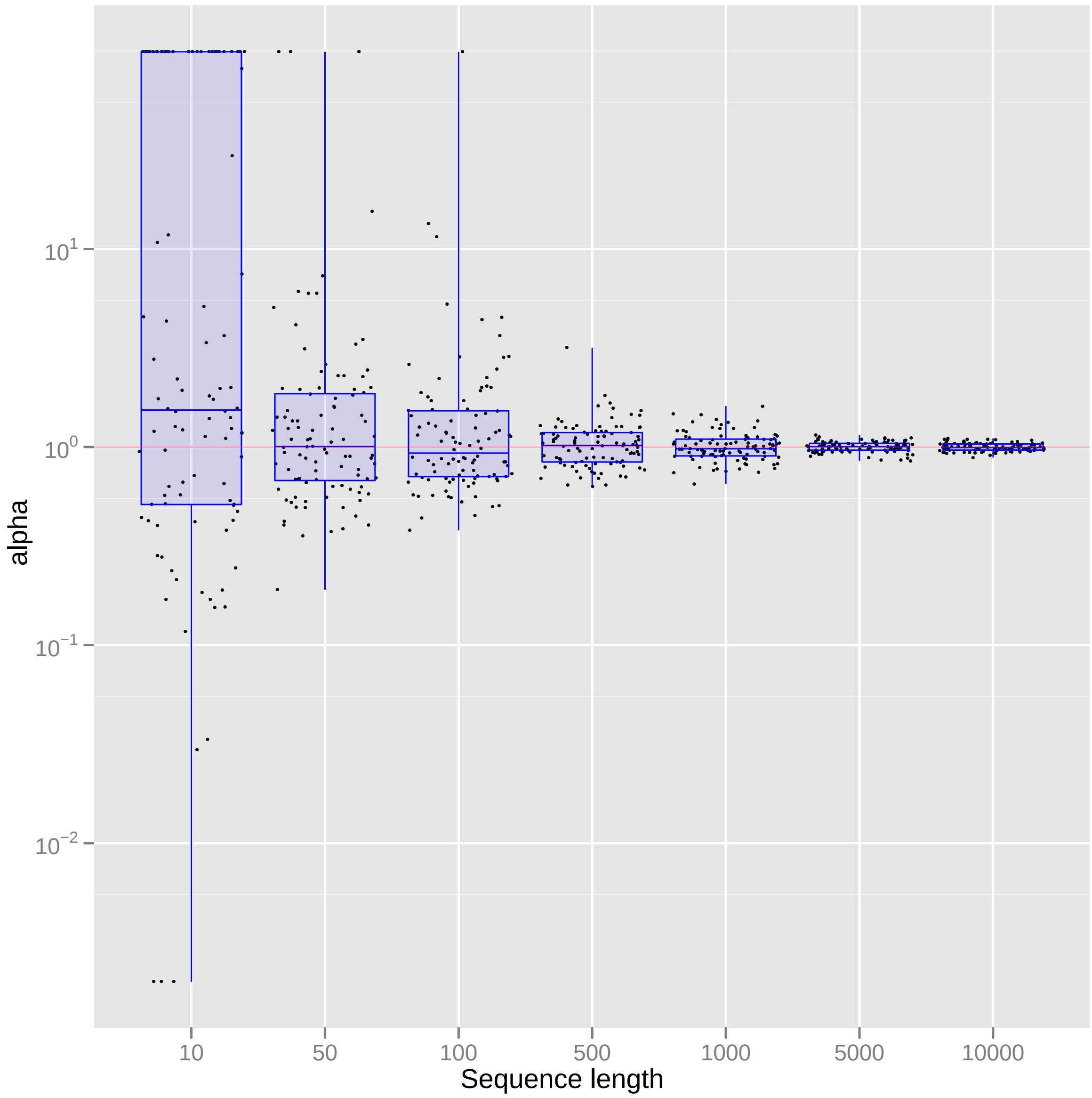

Length of branch 1

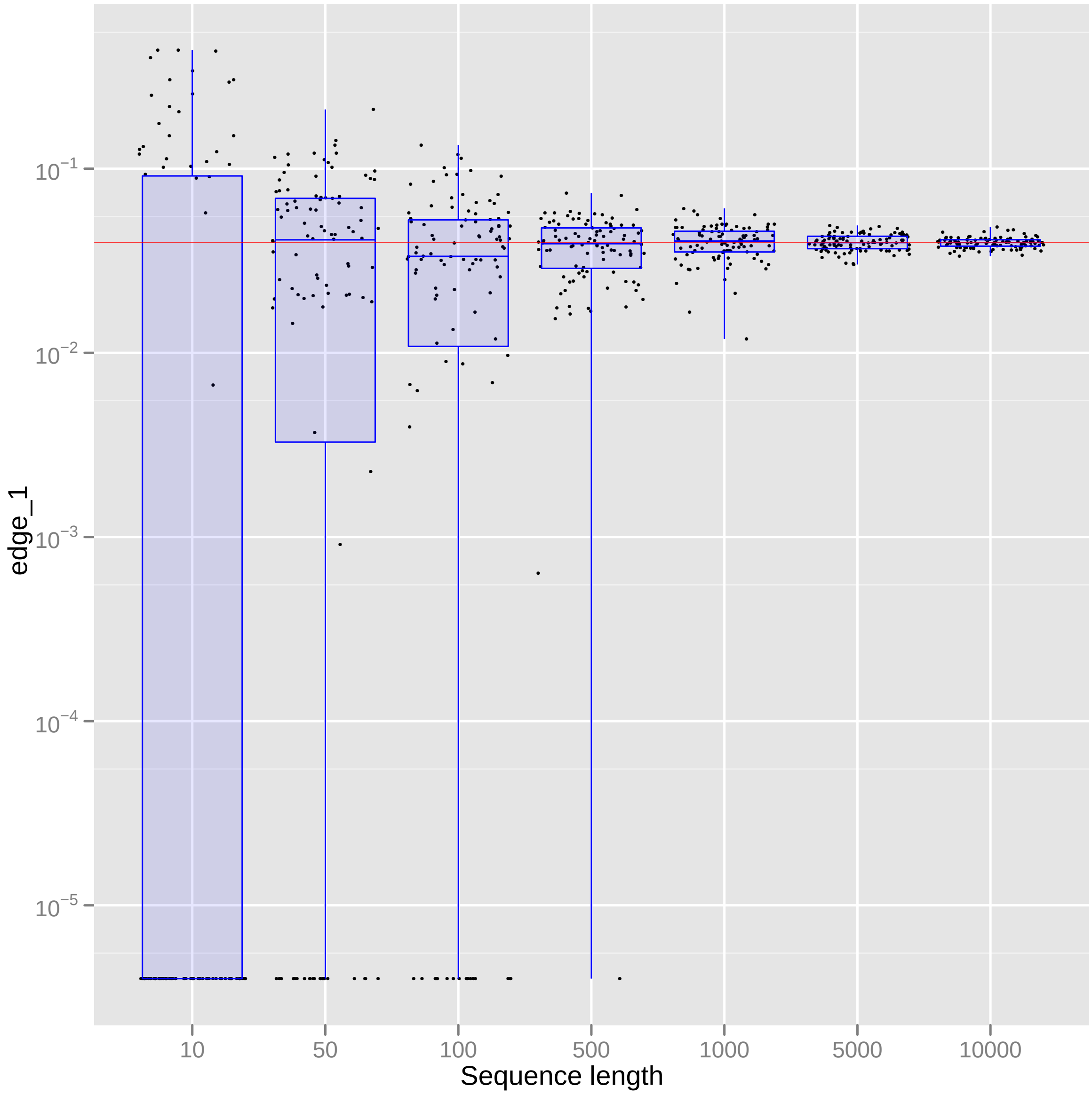

Length of branch 2

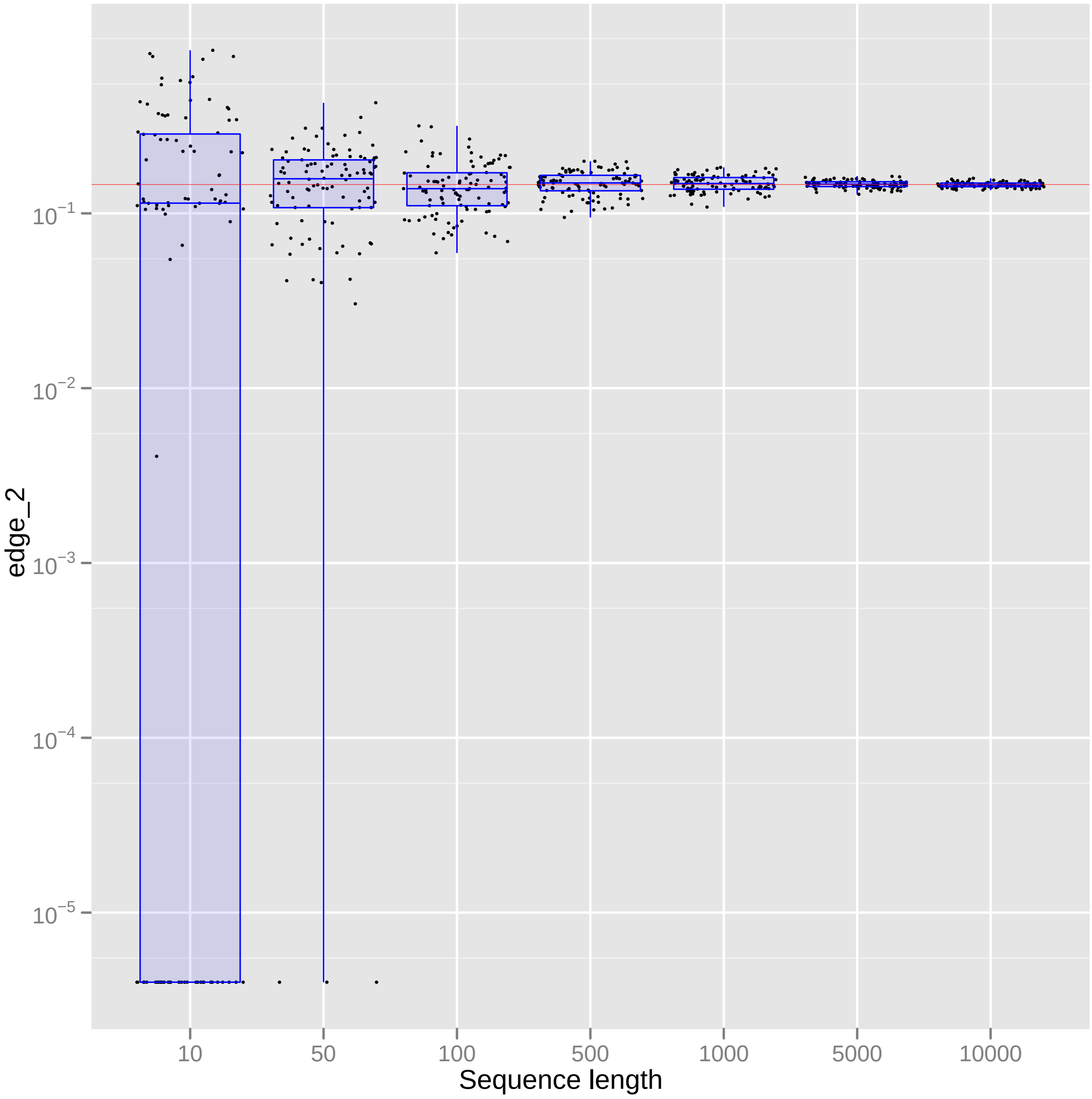

Length of branch 3

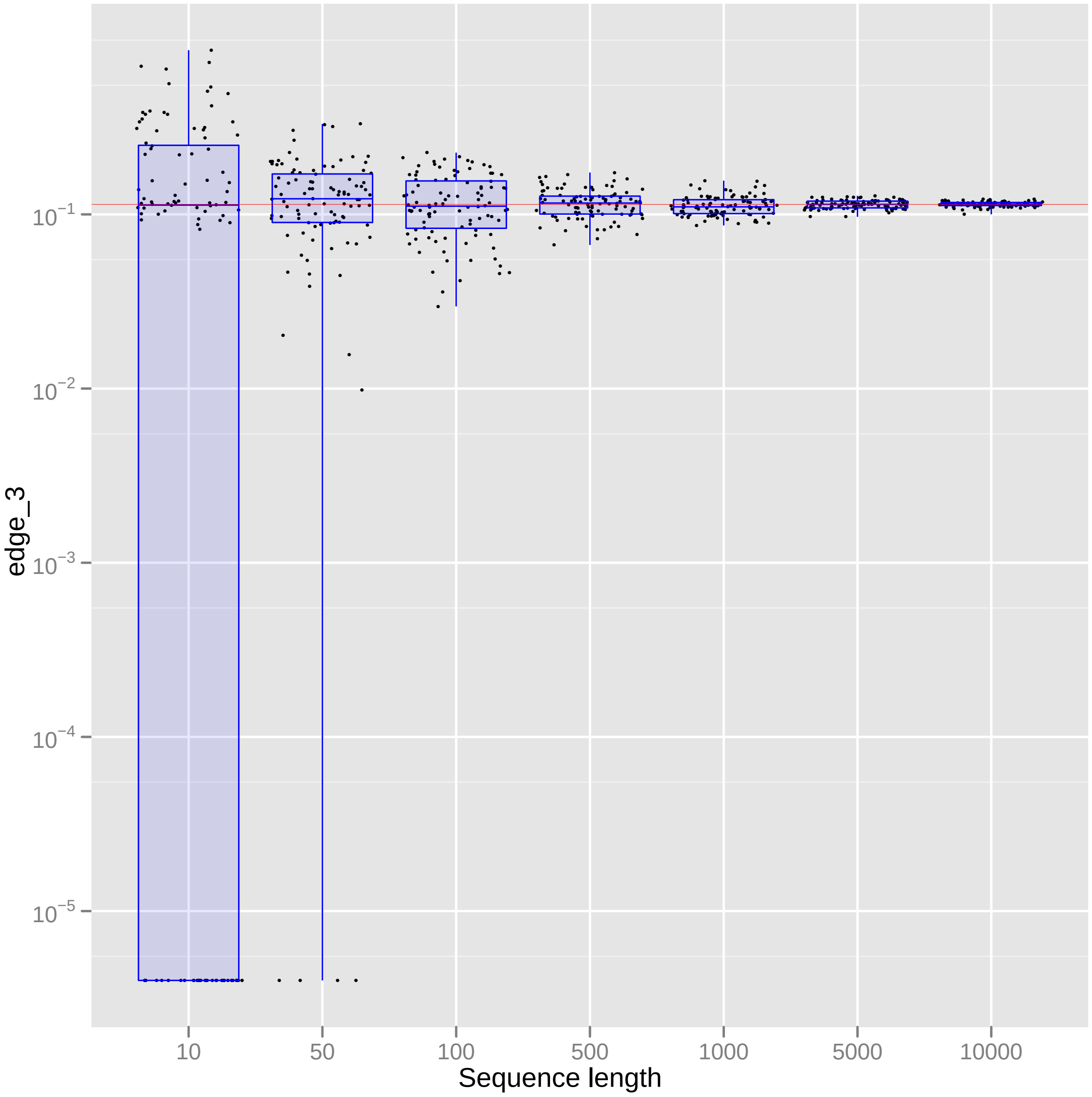

Length of branch 4

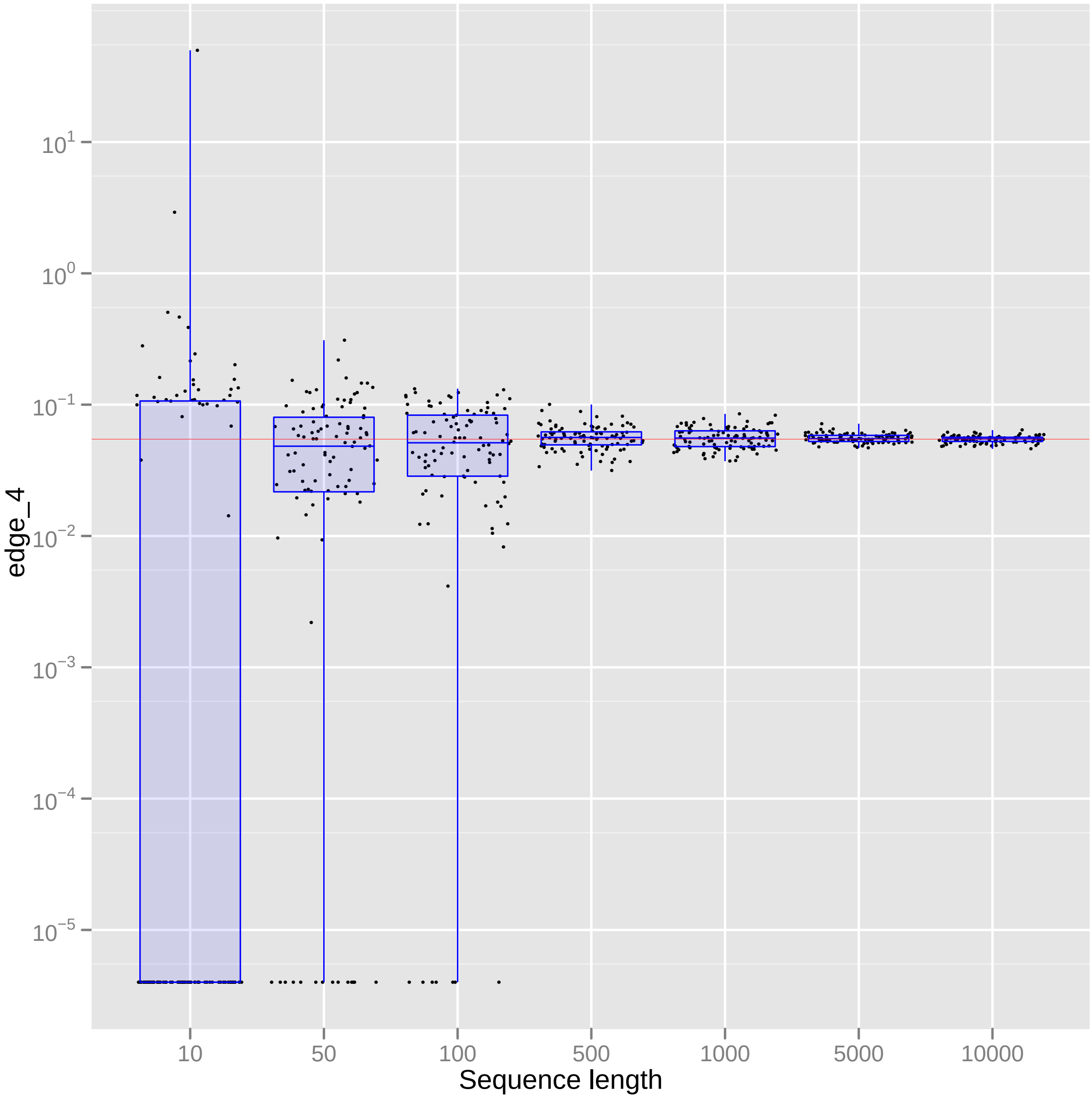

Length of branch 5

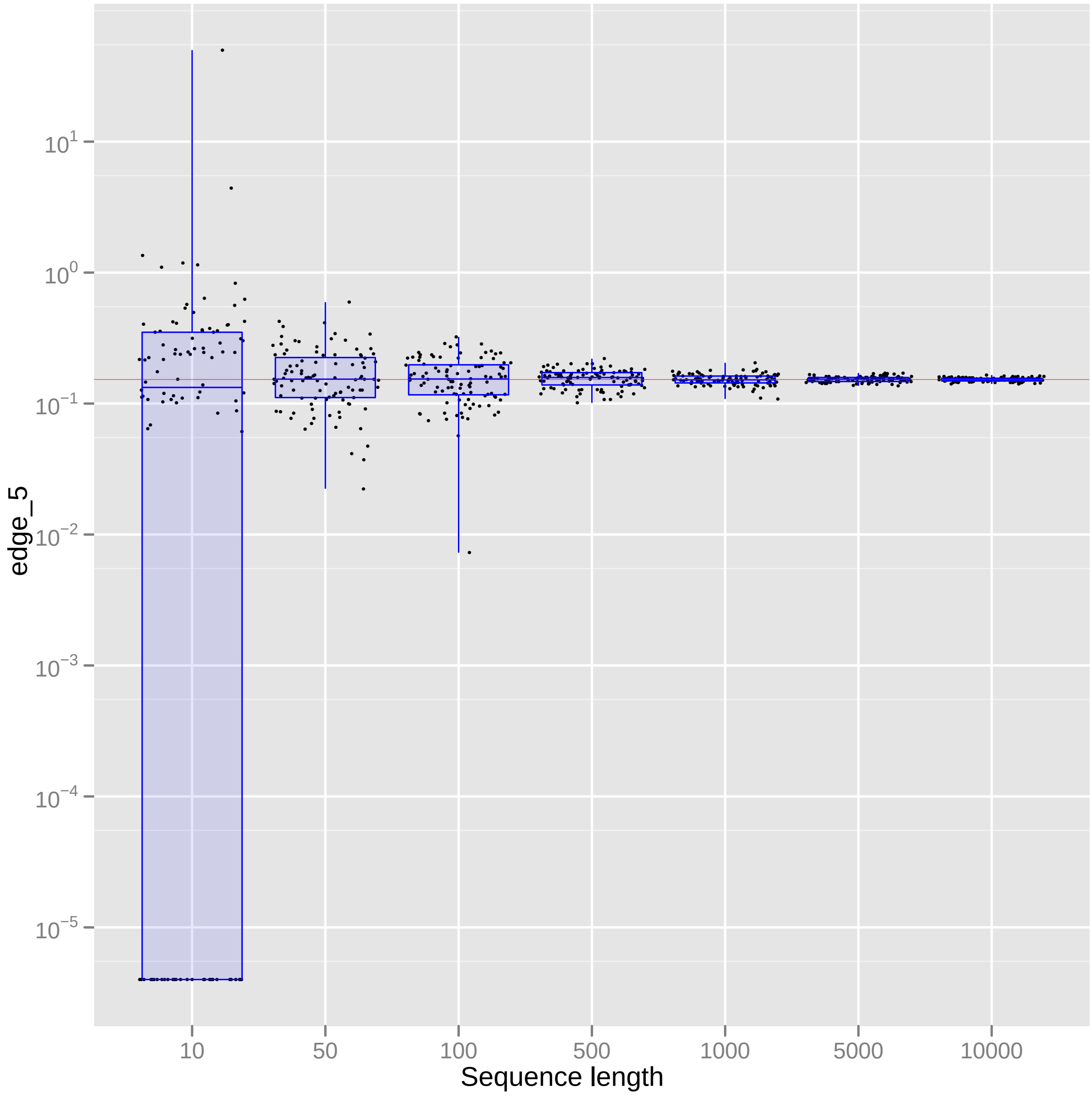

Length of branch 6

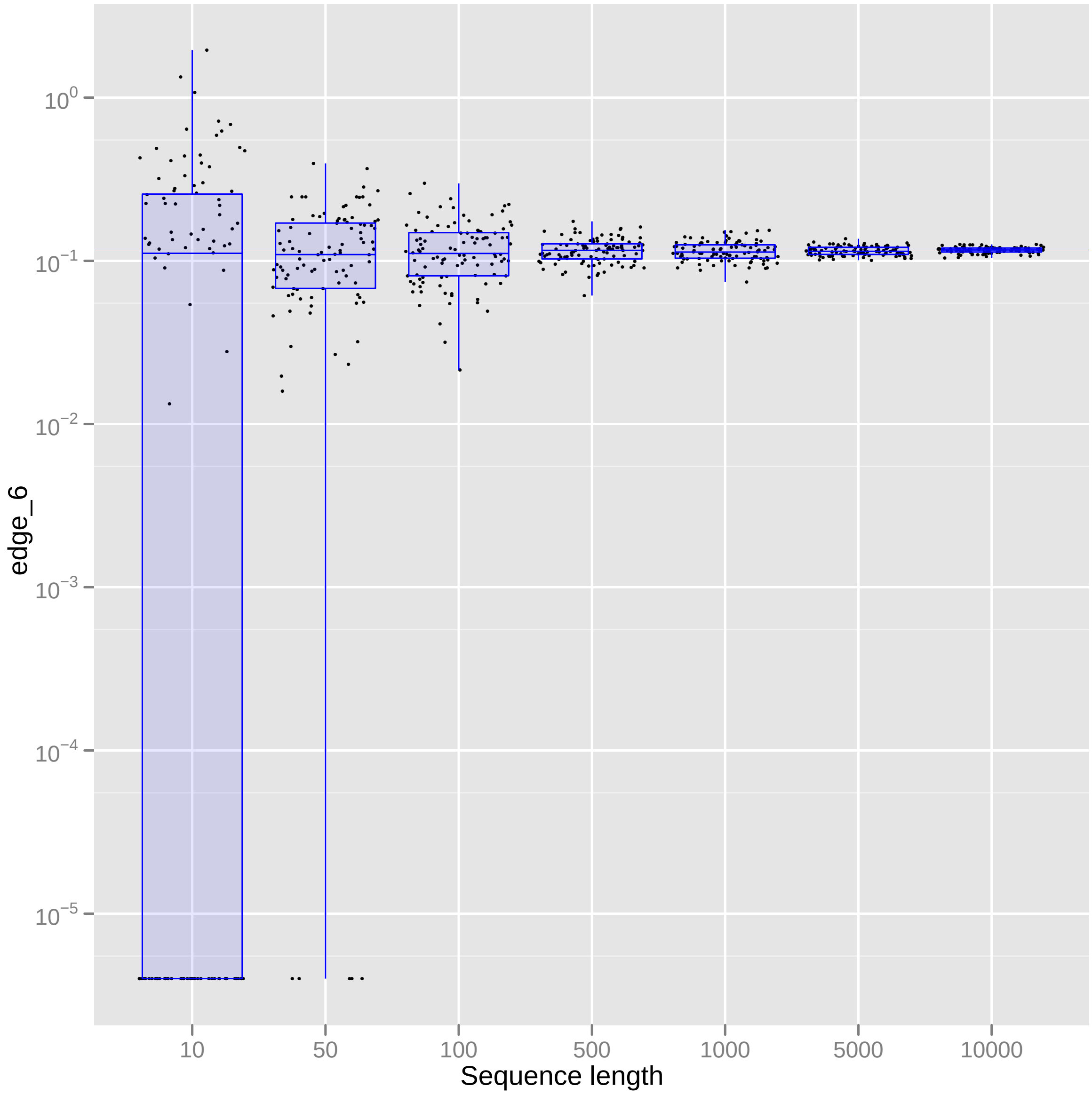

Length of branch 7

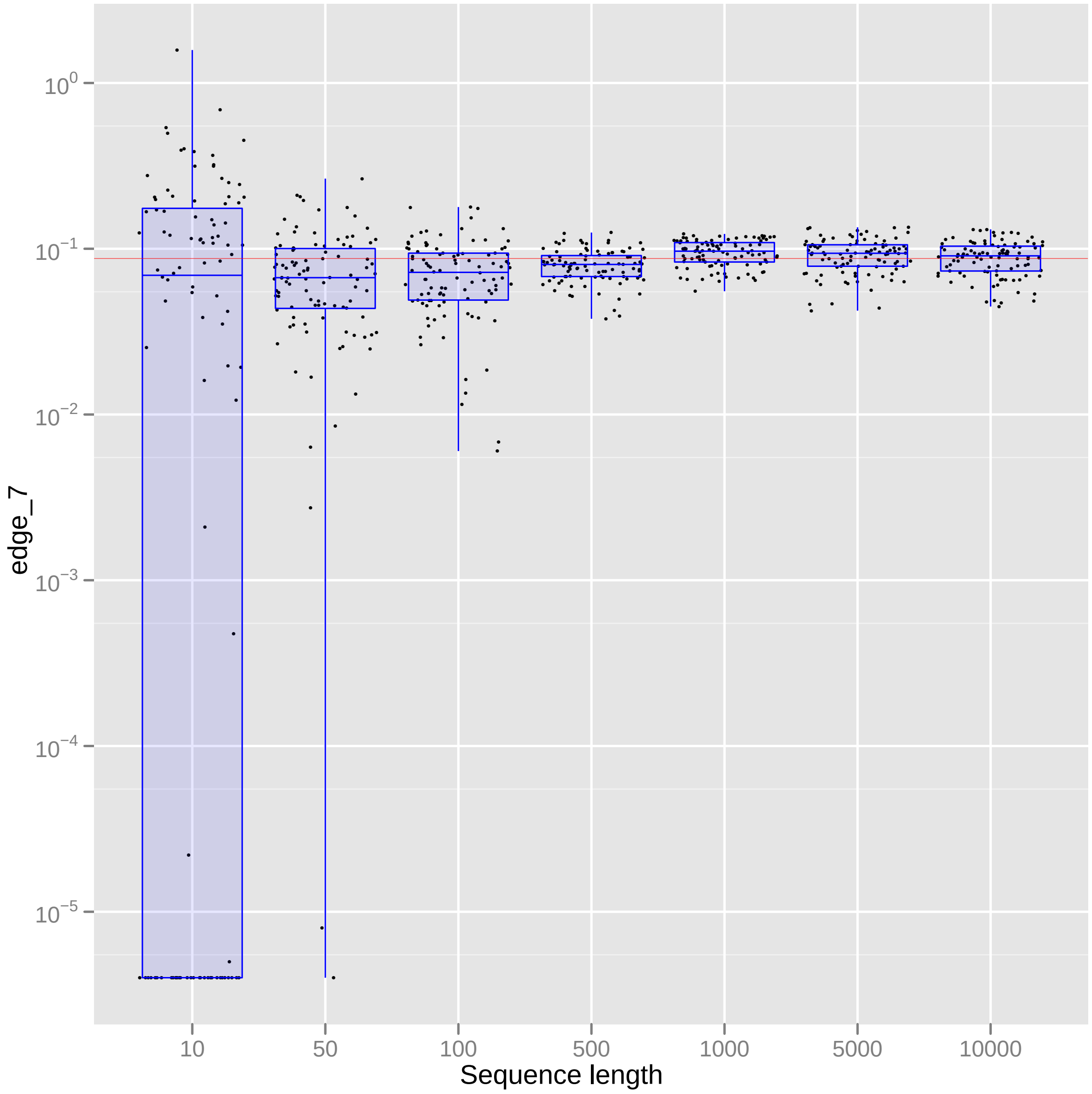

Length of branch 8

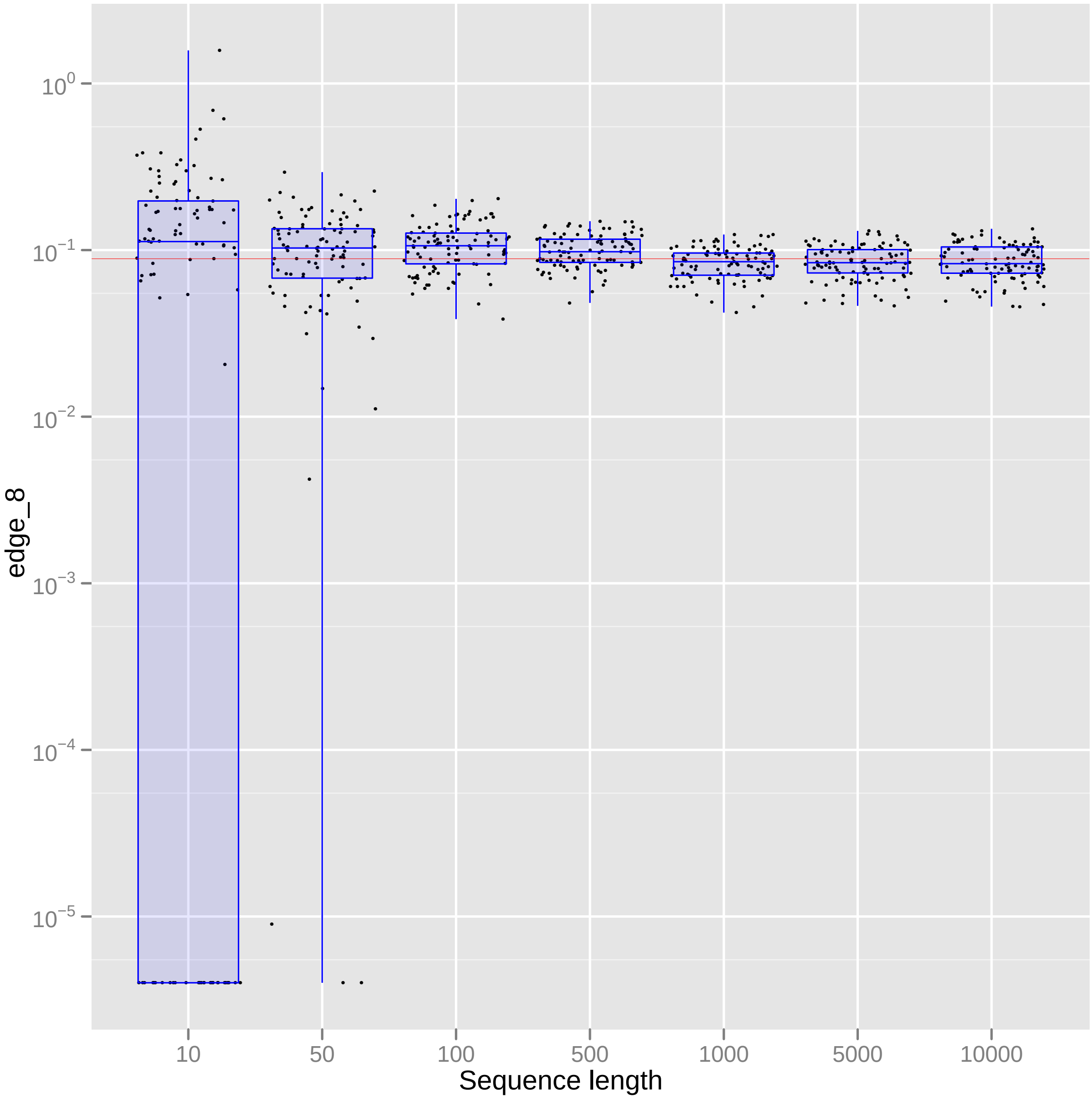

2.3 Codon sequences

We simulated the evolution of codon sequences under the GY94+M0 (a single ω) model, with a transition/transversion rate ratio $\kappa = 4$, nonsynoymous/synonymous rate ratio $\omega = 0.3$, and equal codon frequencies. We scaled the branch lengths to comply with the `PAML`-style scaling of substitution processes (one expected accepted mutation per codon site).

We estimated the model parameters (κ, ω) and the branch lengths from the simulated alignments by using the `codeml` program from the `PAML` package (version 4.4c). We used `F3X4` empirical codon frequencies during the estimation. See the control file (codeml_codon.ctl) for details.

The following plots summarize the results of the simulations. The x axis represents the sequence length , while the logarithmically transformed y axis shows the estimated value of the

parameters (κ, ω and the branch lengths). The x axis values are jittered for better visiblity. The horizontal red line represents the true value of the parameter.

The overlayed box-and-whisker plots indicate the smallest value, the lower quartile (Q1), the median (Q2), the upper quartile (Q3) and the maximum value.

Kappa

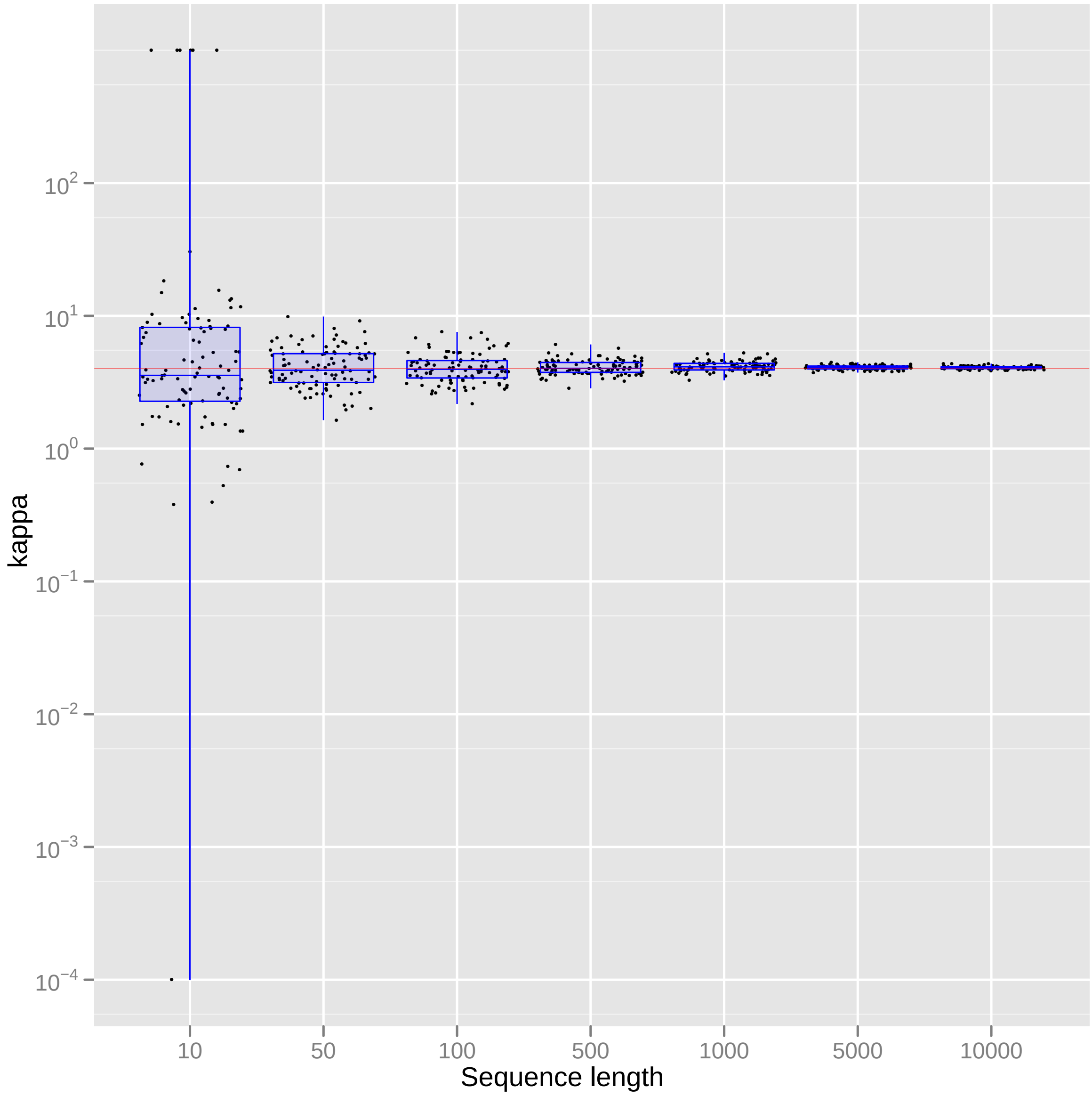

Omega

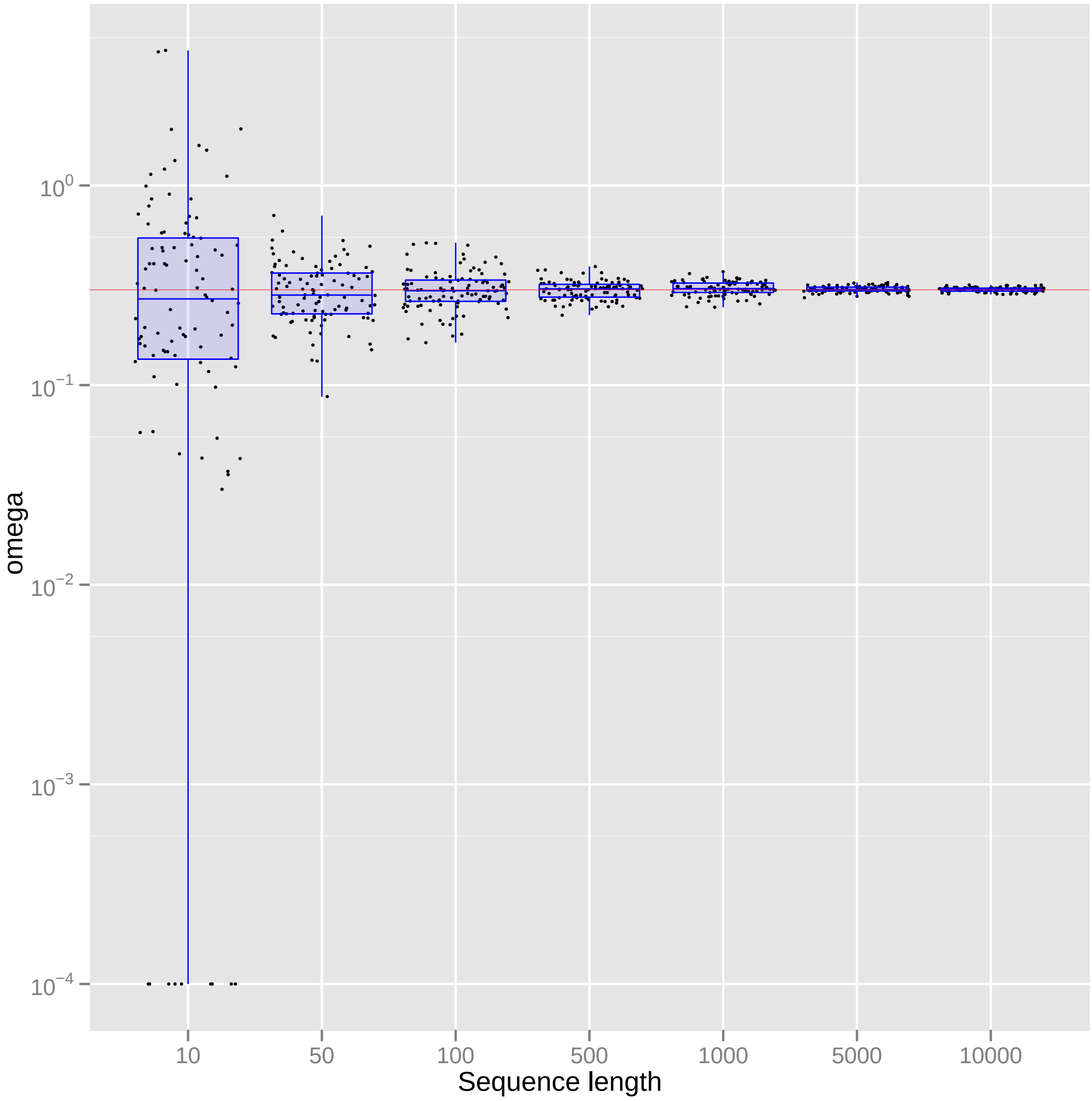

omega
10^0
10^{-1}
10^{-2}
10^{-3}
10^{-4}
10
50
100
500
1000
5000
10000
Sequence length

Length of branch 1

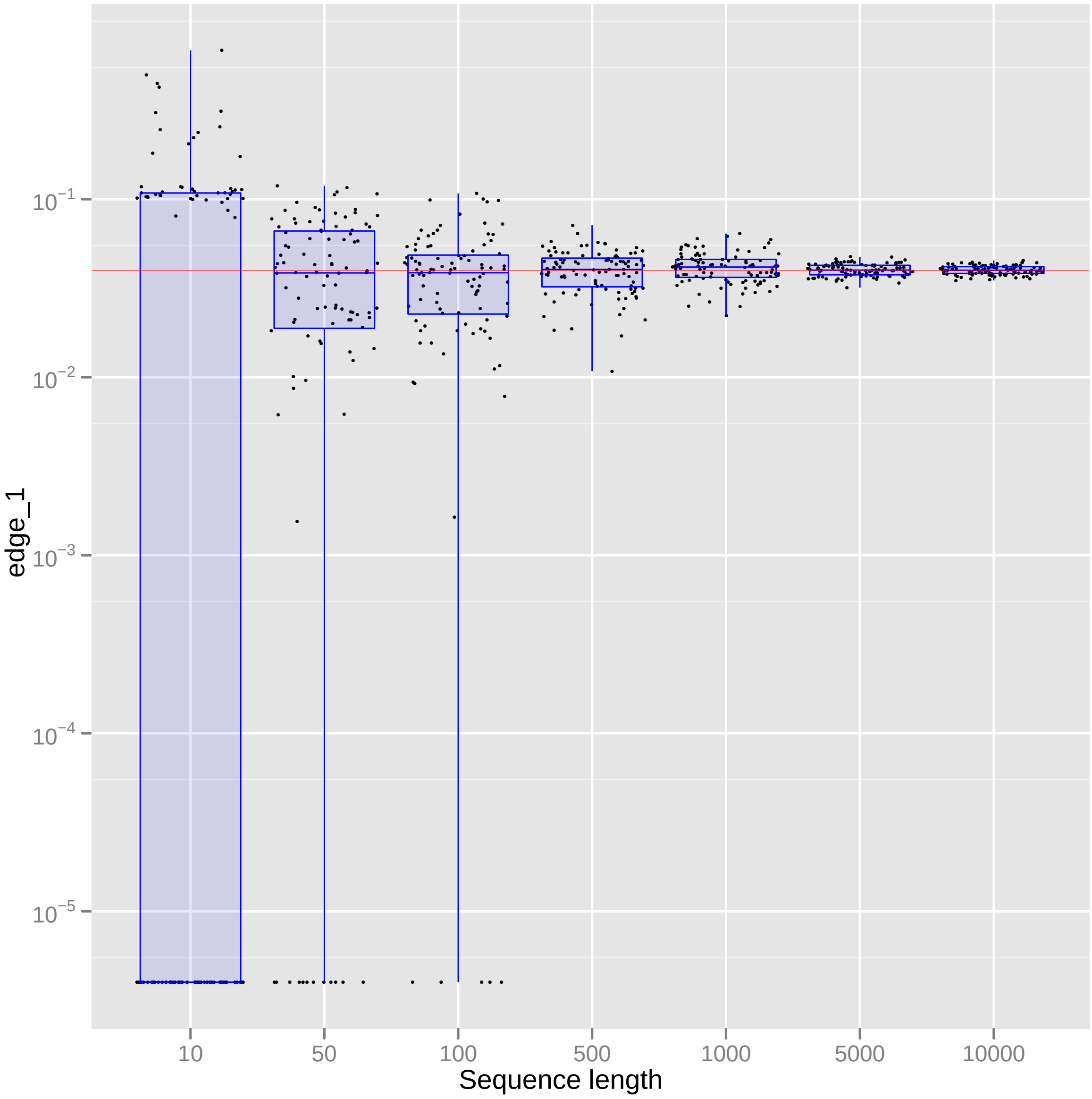

Length of branch 2

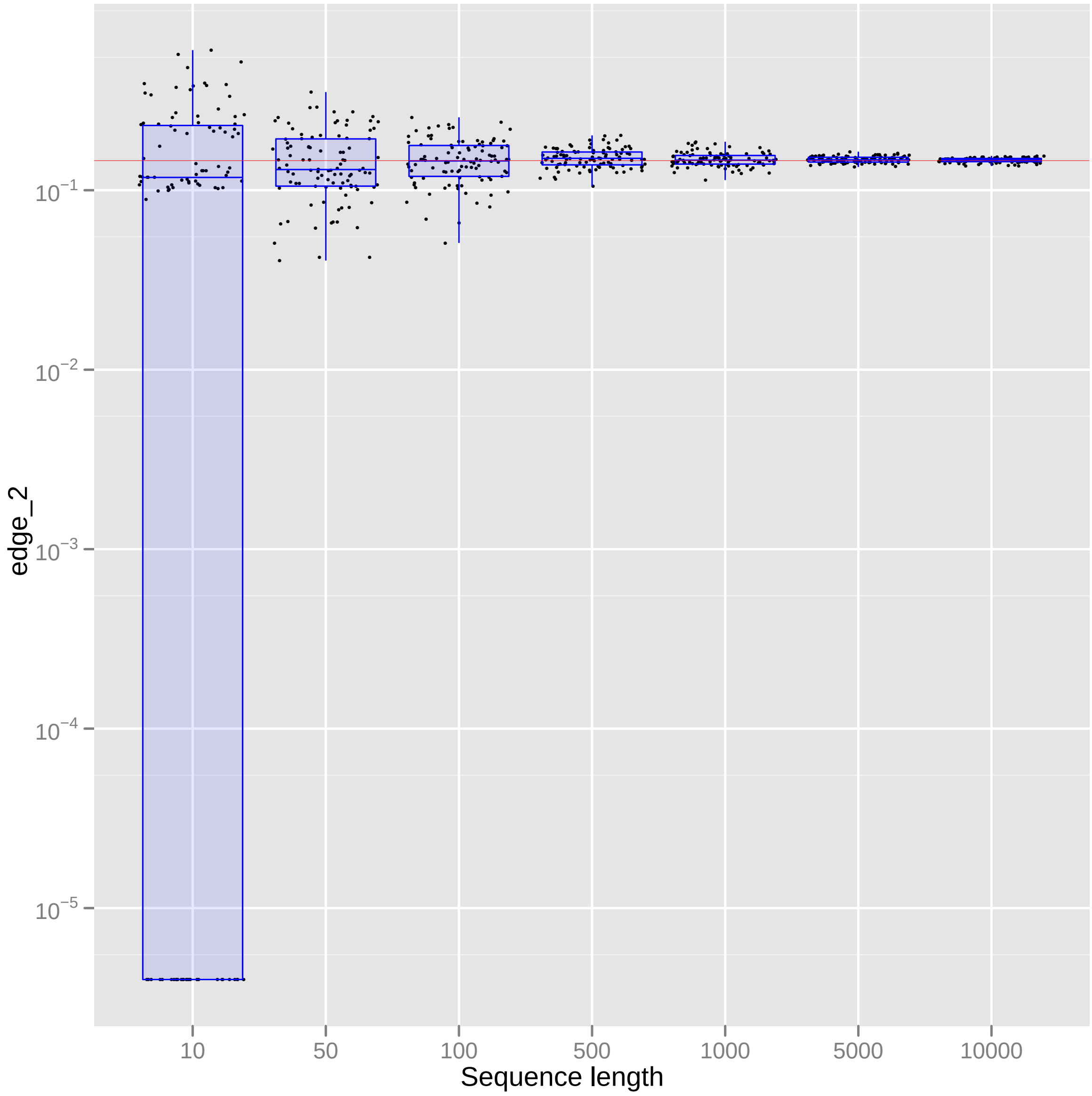

Length of branch 3

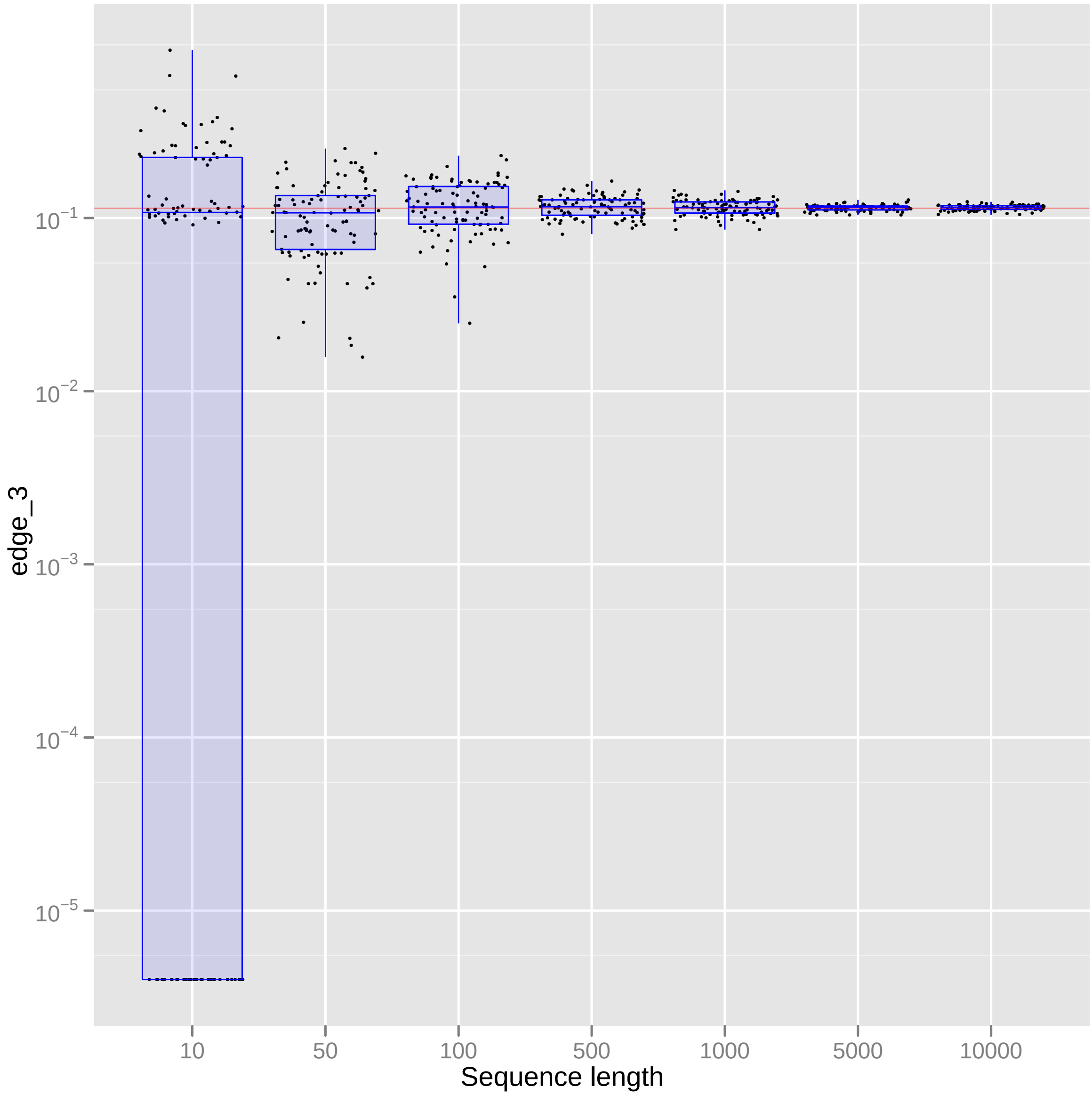

Length of branch 4

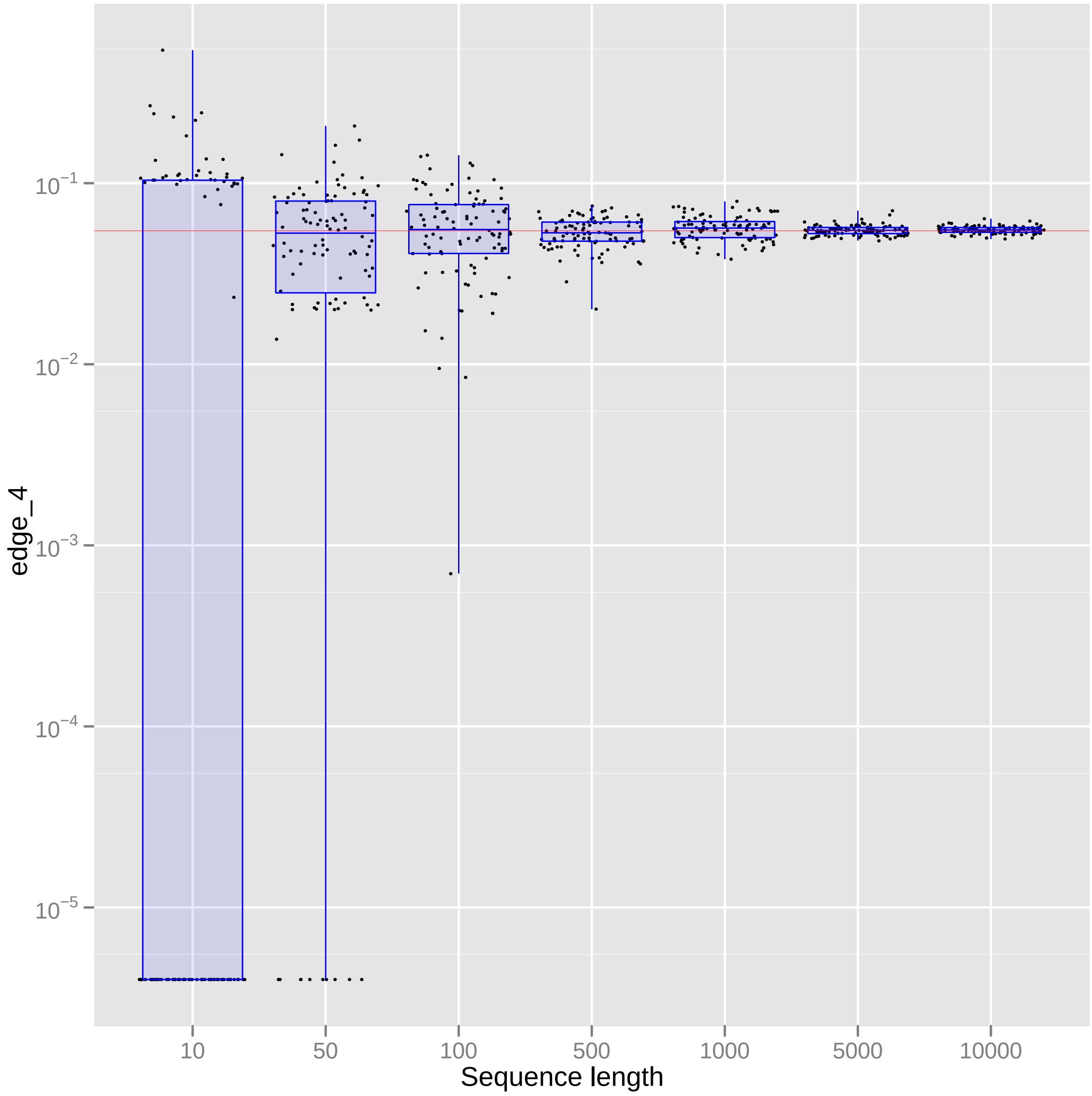

Length of branch 5

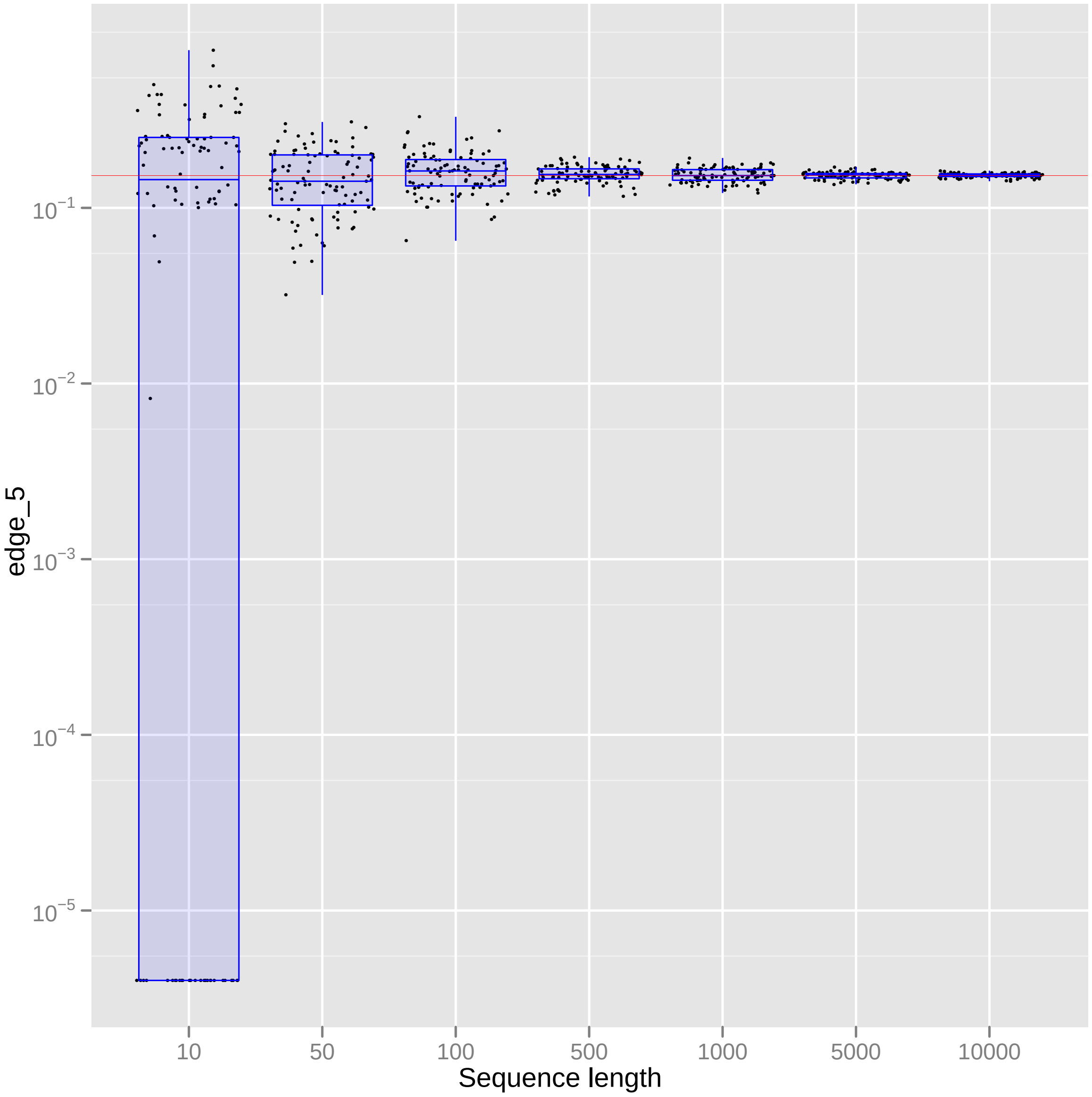

Length of branch 6

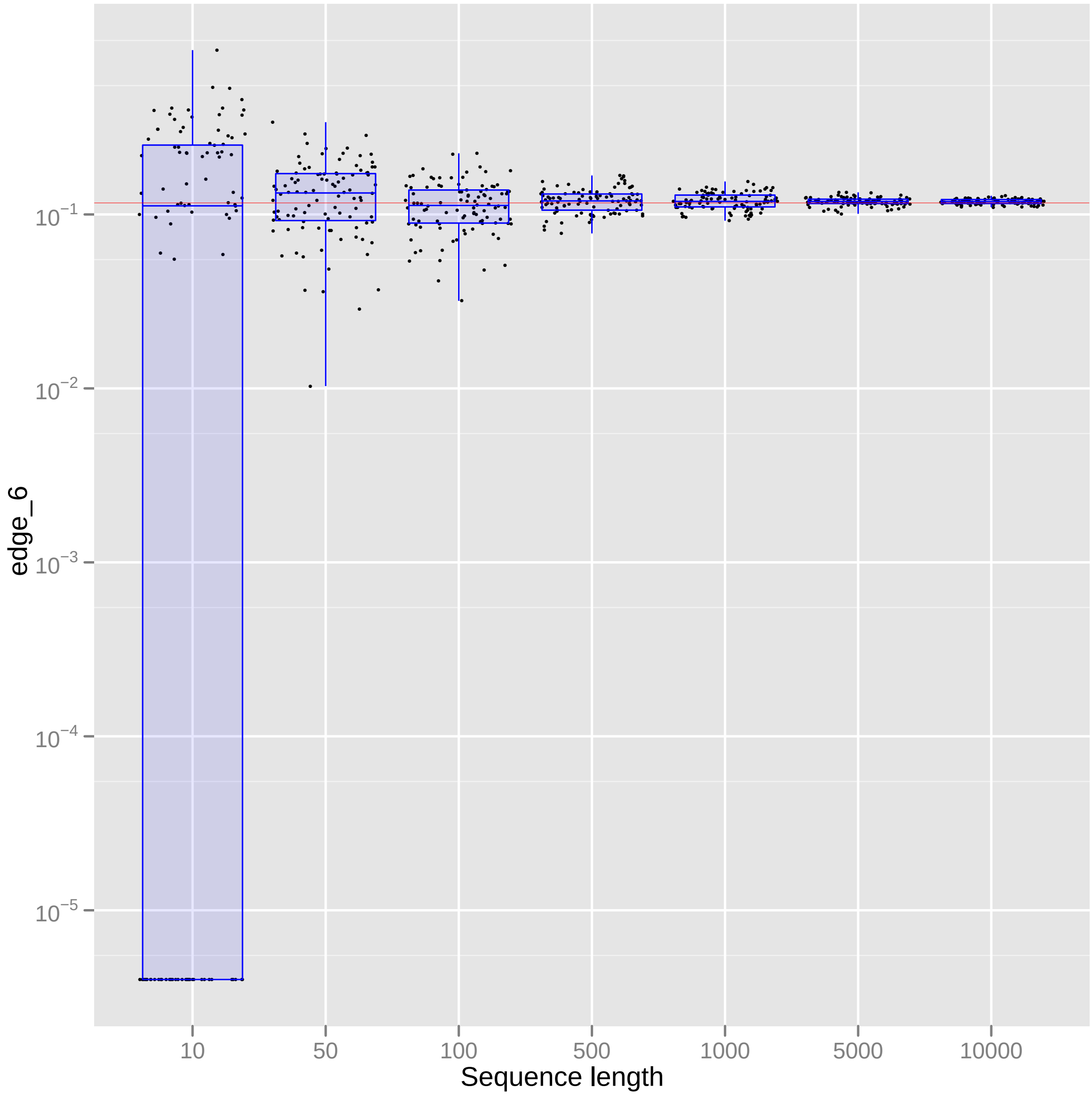

Length of branch 7

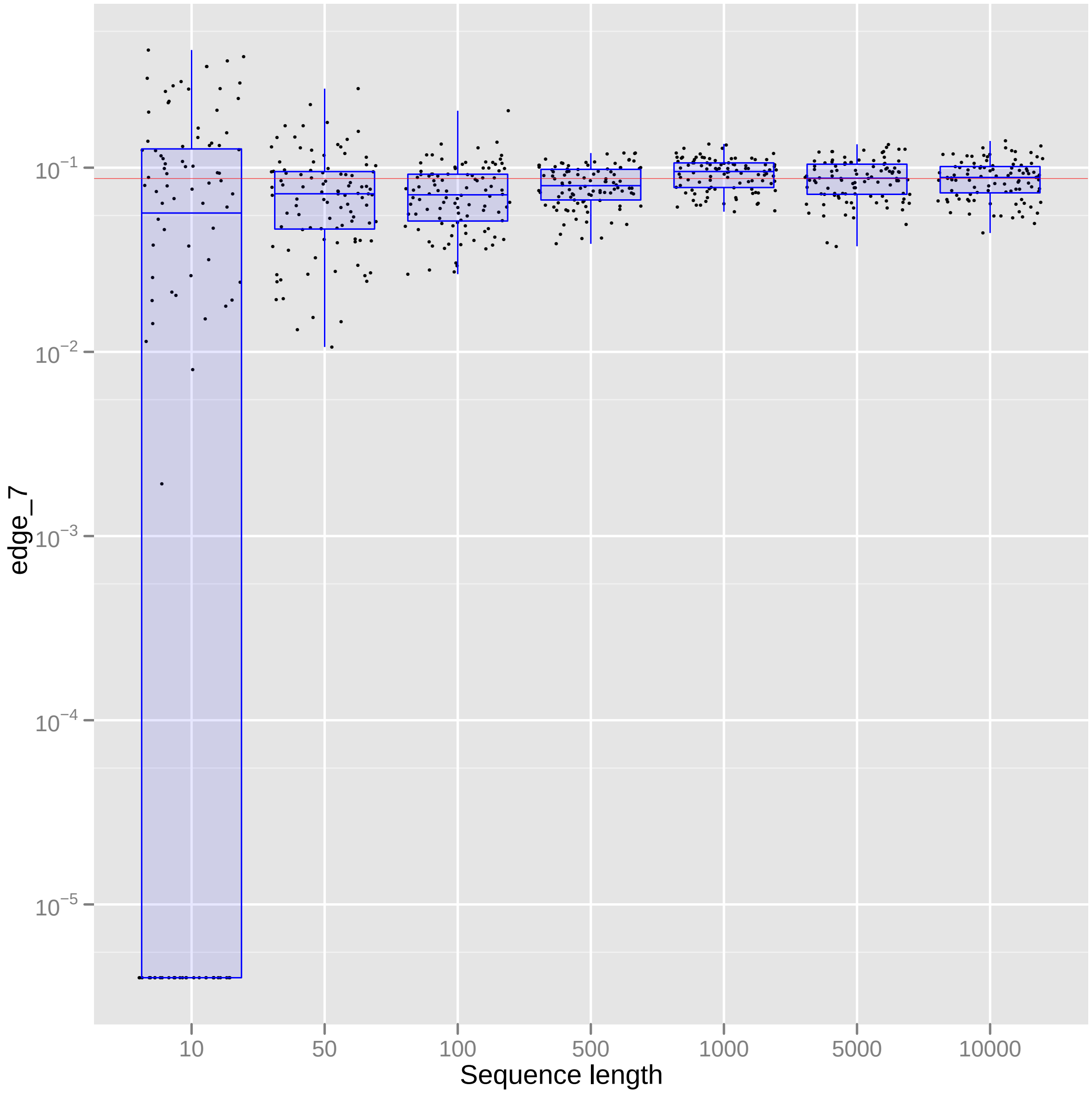

Length of branch 8

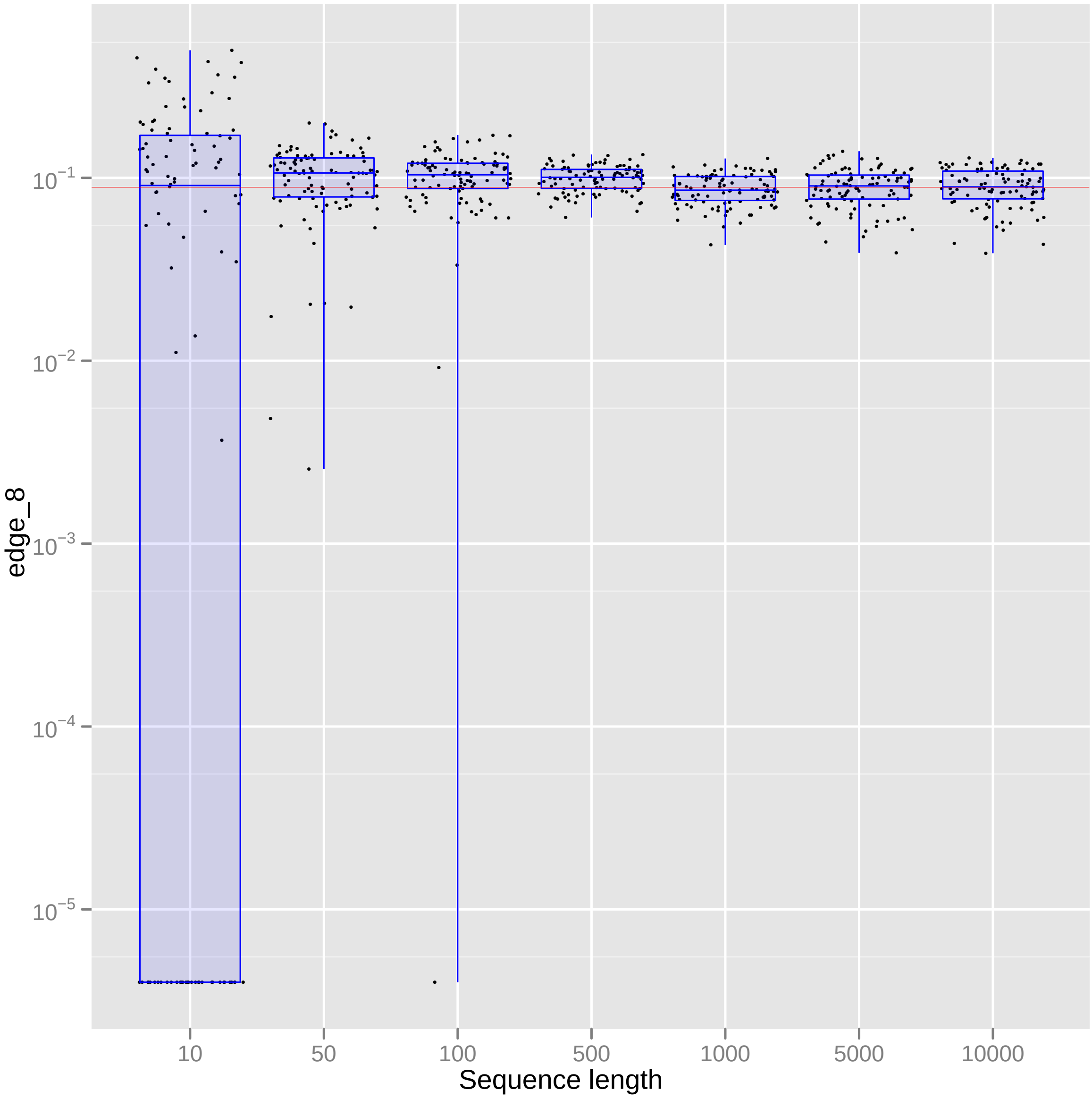

3 Computing time required for the simulations

The following tables give the average number of CPU seconds (CPU: `Intel Xeon 5000 series`) needed to simulate one alignment and to estimate the parameters under the settings described above as the function of the sequence length. Estimation includes the parsing of `PAML` result files in `R`. Please note the the simulations have been run with the `PSIM_FAST` mode enabled.

Nucleotide sequences

Sequence length	10	50	100	500	1000	5000	10000
Simulation	0.733	1.834	3.447	14.773	29.843	168.511	385.268
Estimation	0.013	0.013	0.015	0.021	0.023	0.064	0.123

Amino acid sequences

Sequence length	10	50	100	500	1000	5000	10000
Simulation	1.193	3.652	6.555	28.991	58.582	320.386	693.500
Estimation	0.011	0.012	0.012	0.017	0.024	0.075	0.127

Codon sequences

Sequence length	10	50	100	500	1000	5000	10000
Simulation	0.919	2.581	4.815	21.745	44.224	245.116	550.855
Estimation	0.022	0.023	0.025	0.034	0.041	0.092	0.164
